# Chesapeake Bay Dissolved Oxygen Criterion Attainment Deficit: Three Decades of Temporal and Spatial Patterns

**DOI:** 10.3389/fmars.2018.00422

**Published:** 2018

**Authors:** Qian Zhang, Peter J. Tango, Rebecca R. Murphy, Melinda K. Forsyth, Richard Tian, Jennifer Keisman, Emily M. Trentacoste

**Affiliations:** 1 Chesapeake Bay Program Office, University of Maryland Center for Environmental Science, Annapolis, MD, United States; 2 Chesapeake Bay Program Office, U.S. Geological Survey, Annapolis, MD, United States; 3 Chesapeake Biologicai Laboratory, University of Maryland Center for Environmental Science, Solomons, MD, United States; 4 Maryland-Delaware-District of Columbia Water Science Center, U.S. Geological Survey, Catonsville, MD, United States; 5 Chesapeake Bay Program Office, U.S. Environmental Protection Agency, Annapolis, MD, United States

**Keywords:** water quality standards, dissolved oxygen, criteria attainment, monitoring and assessment, Chesapeake Bay, Mann-Kendall test, ecosystem management, spatial aggregation

## Abstract

Low dissolved oxygen (DO) conditions are a recurring issue in waters of Chesapeake Bay, with detrimental effects on aquatic living resources. The Chesapeake Bay Program partnership has developed criteria guidance supporting the definition of state water quality standards and associated assessment procedures for DO and other parameters, which provides a binary classification of attainment or impairment. Evaluating time series of these two outcomes alone, however, provides limited information on water quality change over time or space. Here we introduce an extension of the existing Chesapeake Bay water quality criterion assessment framework to quantify the amount of impairment shown by space-time exceedance of DO criterion (“attainment deficit”) for a specific tidal management unit (i.e., segment). We demonstrate the usefulness of this extended framework by applying it to Bay segments for each 3-year assessment period between 1985 and 2016. In general, the attainment deficit for the most recent period assessed (i.e., 2014–2016) is considerably worse for deep channel (DC; n = 10) segments than open water (OW; n = 92) and deep water (DW; n = 18) segments. Most subgroups - classified by designated uses, salinity zones, or tidal systems - show better (or similar) attainment status in 2014–2016 than their initial status (1985–1987). Some significant temporal trends (p < 0.1) were detected, presenting evidence on the recovery for portions of Chesapeake Bay with respect to DO criterion attainment. Significant, improving trends were observed in seven OW segments, four DW segments, and one DC segment over the 30 3-year assessment periods (1985–2016). Likewise, significant, improving trends were observed in 15 OW, five DW, and four DC segments over the recent 15 assessment periods (2000–2016). Subgroups showed mixed trends, with the Patuxent, Nanticoke, and Choptank Rivers experiencing significant, improving short-term (2000–2016) trends while Elizabeth experiencing a significant, degrading short-term trend. The general lack of significantly improving trends across the Bay suggests that further actions will be necessary to achieve full attainment of DO criterion. Insights revealed in this work are critical for understanding the dynamics of the Bay ecosystem and for further assessing the effectiveness of management initiatives aimed toward Bay restoration.

## INTRODUCTION

**FIGURE 1 |:**
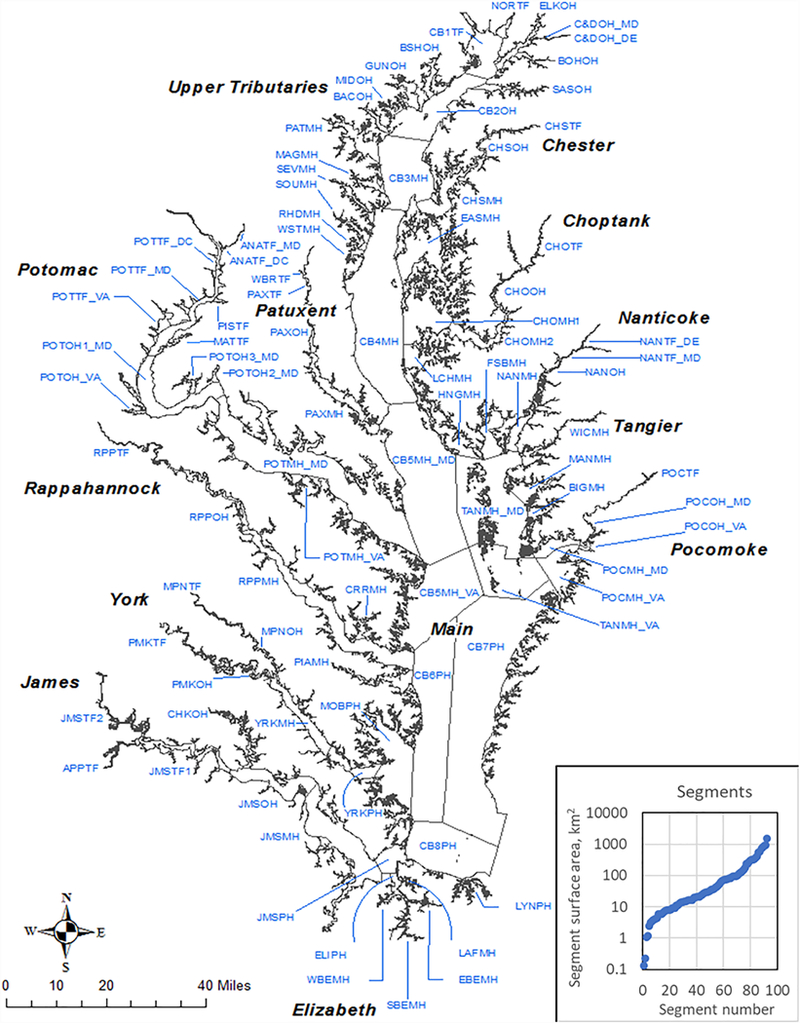
Segmentation scheme used in the assessment of Chesapeake Bay dissolved oxygen criterion attainment (U.S. Environmental Protection Agency [USEPA], 2004a,b).

Chesapeake Bay, the largest estuary in the United States, is an incredibly complex and productive ecosystem that provides habitats, food, and protection for thousands of species of animals and plants (Figure 1). This national treasure, however, has suffered cultural eutrophication for decades, largely due to anthropogenic inputs of nutrient and sediment from its multi-jurisdiction watershed (Boynton et al., 1995; Kemp et al., 2005; Hirsch et al., 2010; Zhang et al., 2013, 2015; Zhang and Blomquist, 2018). Consequently, Chesapeake Bay (“the Bay”) has shown ecological degradation with symptoms such as excessive algal growth, decreased submerged aquatic vegetation acreage, reduced water clarity, and low dissolved oxygen (DO) concentrations (Hagy et al., 2004; Kemp et al., 2005; Murphy et al., 2011; Testa et al., 2017, 2018; Lefcheck et al., 2018).

To support healthy and sustainable living resources in the Bay, the Chesapeake Bay Program (CBP) partnership – which consists of the U.S. Environmental Protection Agency (USEPA), other federal agencies, local and state jurisdictions, and academic and non-governmental organizations – has been committed to the protection of water quality and habitat conditions in the Bay and its tidal tributaries. In 2003, the CBP partnership put forth a guidance framework to establish water quality criteria for DO, water clarity, and chlorophyll-a for the Bay (U.S. Environmental Protection Agency [USEPA], 2003a), which were subsequently adopted into the tidal states’ water quality standards to define which waters are impaired under the Clean Water Act (Supplementary Table S1 in Appendix A). In addition, this guidance framework (U.S. Environmental Protection Agency [USEPA], 2003a) has set the foundation for criteria attainment assessment procedures, which have been periodically refined as new knowledge has become available (U.S. Environmental Protection Agency [USEPA], 2003a, 2004a, 2007a,b, 2008, 2010a, 2017).

**FIGURE 2 |:**
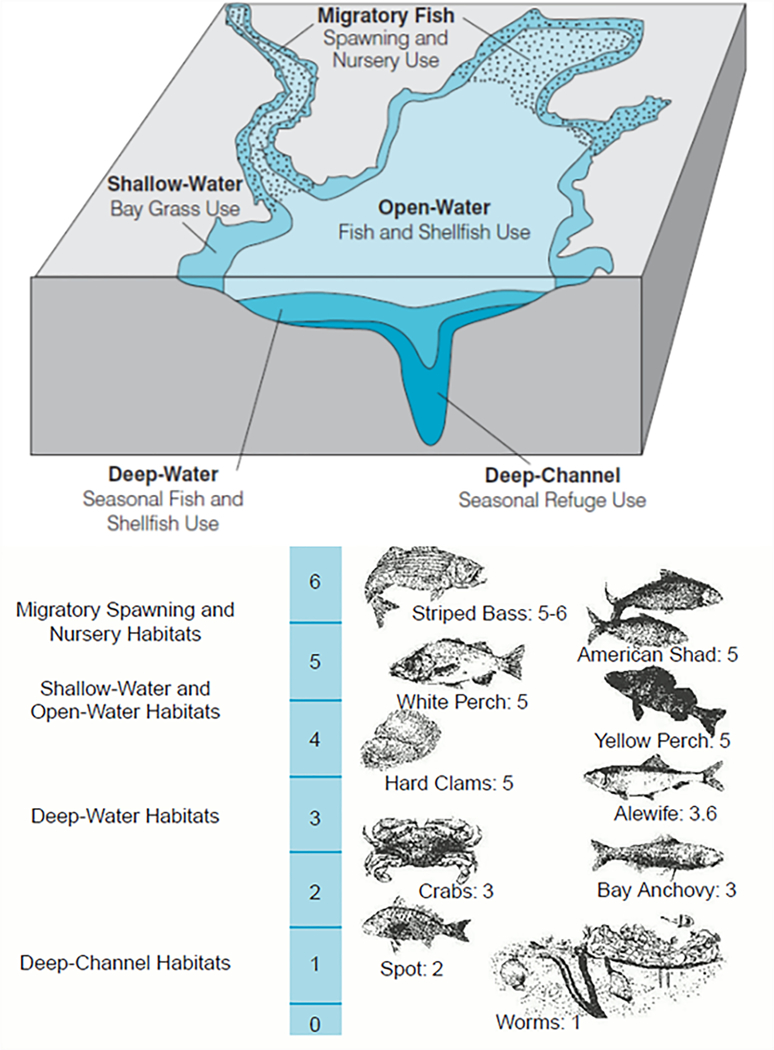
The five designated uses in Chesapeake Bay dissolved oxygen criterion attainment assessment. **(Top)** Conceptual illustration. **(Bottom)** Dissolved oxygen (mg L^‒1^) concentrations required by different Chesapeake Bay species and communities (U.S. Environmental Protection Agency [USEPA], 2003a,b, 2004b).

Water quality criteria are applied for five different designated uses (DUs) of aquatic habitats, namely, open water (OW), deep water (DW), deep channel (DC), migratory spawning and nursery (MSN), and shallow water (SW). These DUs reflect the nature of water column structure and the life history needs of living resources, which vary seasonally (Figure 2 and Supplementary Table S1; U.S. Environmental Protection Agency [USEPA], 2003b, 2004b). In particular, the OW criterion protects diverse populations of sport fish, including striped bass, bluefish, mackerel and sea trout, as well as important bait fish such as menhaden and silversides. The DW criterion protects animals inhabiting the deeper transitional water-column and bottom habitats between the well-mixed surface waters and the deep channels, including many bottom-feeding fish, crabs, and oysters. The DC criterion protects bottom sediment dwelling worms and small clams that bottom-feeding fish and crabs consume. The MSN criterion protects migratory and resident tidal freshwater fish during the spawning and nursery season in low-salinity habitats. Lastly, the SW protects the many species that depend on vegetated shallow-water habitats. SW is part of the OW and uses the same DO criterion as the OW, although it has separate criteria on submerged aquatic vegetation/water clarity.

We recently published results for the Chesapeake Bay water quality standards attainment indicator (Zhang et al.,2018 which aggregates the estimated condition of all 92 Chesapeake Bay management segments (Figure 1) for DO, submerged aquatic vegetation/water clarity, and chlorophyll-a criteria that are evaluated for addressing the goal of meeting the requirements of the Chesapeake Bay Total Maximum Daily Load (U.S. Environmental Protection Agency [USEPA], 2010b). Our current work expands upon that effort by delving into the attainment results of each individual segment. Specifically, we extend the utility of the existing assessment framework beyond the binary pass/fail classification to quantify the actual amount of space-time criterion exceedance, which we call “attainment deficit.” This was motivated by our observations that segments may have drastically different status and trends in the extent of their impairment while showing no state change with respect to attainment status. Thus, tracking spatial and temporal patterns of the attainment deficit has the potential to reveal further information on water quality dynamics, as compared with our prior effort employing a binary pass/fail classification.

In this work, we demonstrate the usefulness of this extended framework by applying it to all applicable Bay segments for each of the 30 3-year periods between 1985 and 2016 (i.e., 1985–1987, 1986–1988 … 2014–2016). This comprehensive assessment of DO criterion attainment include (1) a synthesis of DO criterion attainment deficit for three DUs (i.e., OW, DW, and DC) for the 92 segments listed in the Chesapeake Bay Total Maximum Daily Load document (the MSN DU is excluded due to data insufficiency; the SW DU is also excluded because it is part of the OW DU with respect to DO); and (2) a synthesis of DO criterion attainment deficit for aggregated subgroups in the Chesapeake Bay ecosystem. Subgroups are defined as the aggregation of all segments that belong to a specific DU (*n* = 3), salinity zone (*n* = 4), or tidal system (*n* = 13). These results provide essential information to the Bay management and research community for (1) understanding the conditions and dynamics of the Chesapeake Bay ecosystem and (2) further assessing the effectiveness of management initiatives aimed toward Bay restoration under the influences of climatic and hydrological variability. This work also features Chesapeake Bay as a prime example where long-term monitoring network and science-based criterion assessment methods can be combined to evaluate the status and trends of complex ecosystems, which might be relevant to other coastal and inland ecosystems that are facing ecological degradation (Borja et al., 2008; Bricker et al., 2008; Patricio et al., 2016; Schiff et al., 2016; Sherwood et al., 2016; Trowbridge et al., 2016).

## THE CRITERION ASSESSMENT FRAMEWORK

### The Existing Framework

**FIGURE 3 |:**
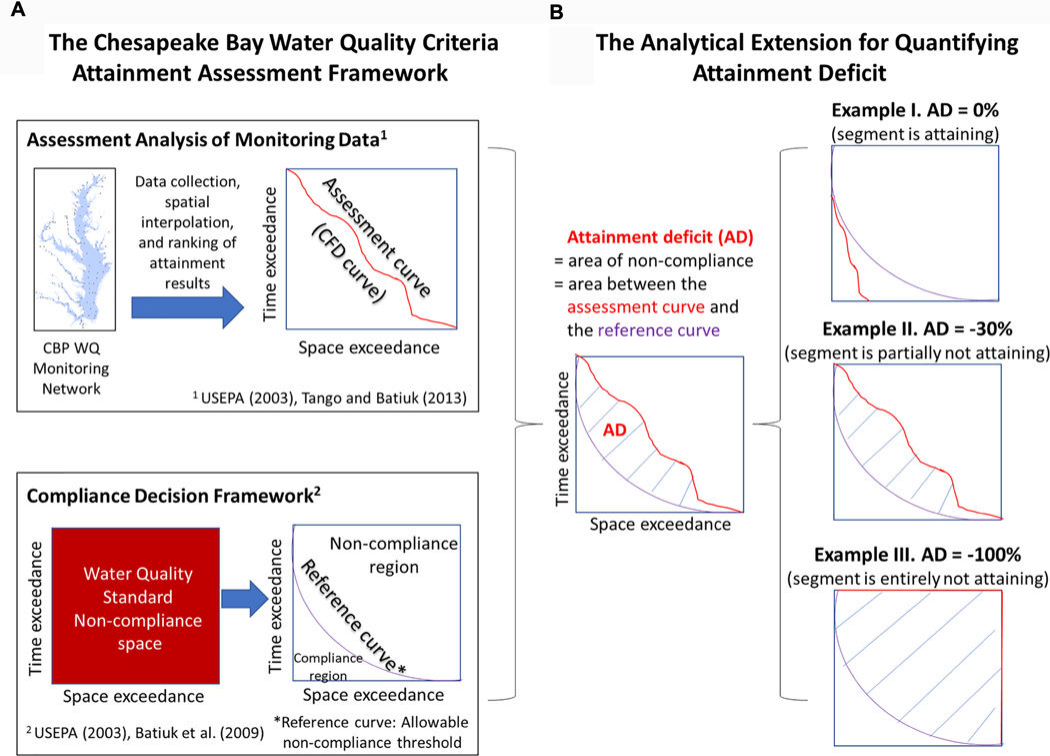
Illustration of **(A)** the existing Chesapeake Bay water quality criteria attainment assessment framework and **(B)** the analytical extension for quantifying attainment deficit.

The existing Chesapeake Bay water quality criteria attainment assessment framework is centered on the development of cumulative frequency distribution (CFD) curves that allow or the evaluation of criteria exceedance (U.S. Environmental Protection Agency [USEPA], 2003a; Batiuk et al., 2009; Tango and Batiuk, 2013). As illustrated in Figure 3A, this assessment framework involves two key components, namely, “Assessment Analysis of Monitoring Data” and “Compliance Decision Framework.”

For the “Assessment Analysis of Monitoring Data,” the framework requires the collection of tidal monitoring data, including DO concentrations, water temperature, and salinity. These data are interpolated using the CBP’s spatial-interpolation software (or “CBP interpolator”) for each spatial unit (U.S. Environmental Protection Agency [USEPA], 2003a). The spatial units are defined by the intersection of Bay segments (Figure 1) and tidal-water DUs (Figure 2). In this regard, water temperature and salinity observations are used to compute the vertical density structure of the water column and delineate boundaries between the OW, DW, and DC layers, which can vary temporally due to freshwater inputs, tides, and other physical conditions. For each spatial unit, DO concentration data are horizontally and vertically interpolated and then compared with appropriate season-specific criterion values (Supplementary Table S1) to quantify the spatial extent of criteria exceedance for each sampling event. For each spatial unit, the estimated spatial exceedance for each sampling event is ranked from the lowest to the highest to construct a CFD curve (also called “attainment curve”), the area below which represents the cumulative amount of space and time in which the criterion value is exceeded.

For the “Compliance Decision Framework,” reference curves have been developed by the CBP Partnership to provide a scientifically based, direct measure of the allowable criteria exceedance, i.e., the amount of criteria exceedance that can occur without causing significant ecological degradation. Readers are referred to U.S. Environmental Protection Agency [USEPA] (2003a) for more details. In the non-compliance space-time assessment space, the reference curve defines the boundary of compliance and impairment. Specifically, the area below the reference curve represents the allowable criteria exceedance (U.S. Environmental Protection Agency [USEPA], 2003a; Batiuk et al., 2009).

The CFD (attainment) curve is compared with the reference curve to determine the status of the spatial unit with respect to criterion attainment. If the CFD curve is not entirely below the reference curve, then the spatial unit is considered “not attaining” the DO criterion.

### The Analytical Extension

Here we introduce an analytical extension to the existing assessment framework (Figure 3B). This extension allows for further exploration of the CFD curve to quantify attainment deficit in a spatial unit, i.e., the intersection area between the attainment curve and the reference curve. This intersection area, also termed the “non-allowable criteria exceedance,” is scaled by the total area of the assessment space to convert to a value in the range of 0 and 100%, which is then converted to attainment deficit by adding a minus sign. In other words, a criteria exceedance of 0% corresponds to an attainment deficit of 0%, whereas a criteria exceedance of 100% corresponds to an attainment deficit of –100%.

Attainment deficit is always in the range of 0 and –100%; see three representative examples in Figure 3B. An attainment deficit of 0%, which is the best possible condition, implies that the minimum water quality requirements are met for providing protection to aquatic life in the defined zones. An attainment deficit of –100%, which is the worst possible condition, implies complete non-compliance. Any other values also indicate non-compliance, with values closer to –100% implying more severe conditions that have substantial negative effects on living resources’ survival, growth, and reproduction.

**FIGURE 4 |:**
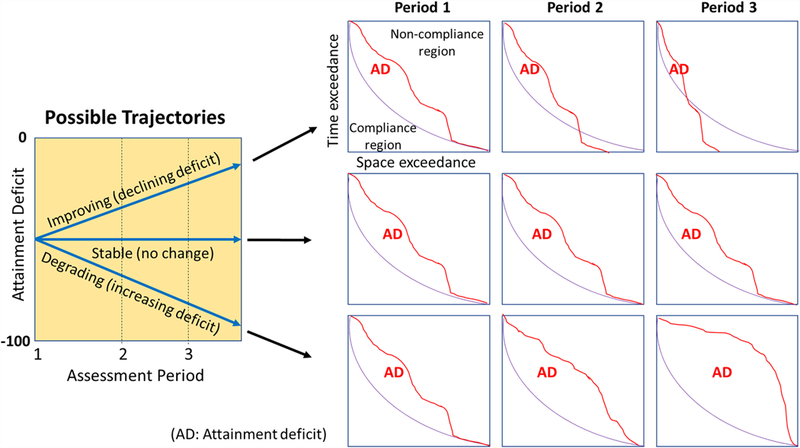
Three possible trajectories of attainment deficit over time, representing three types of temporal change in criterion attainment - i.e., improving, stable, and degrading conditions.

One major benefit of quantifying attainment deficit is to enhance our analytical capability to detect temporal changes. Many segment-DUs may not have experienced a state change using the binary pass/fail attainment classification, but they may have experienced drastically different trends in the extent of their non-compliance (or attainment deficit). This is illustrated in Figure 4 with three simplified trajectories, which show an improving condition (i.e., declining attainment deficit), a stable condition (i.e., no significant change in attainment deficit), and a degrading condition (i.e., increasing attainment deficit), respectively. This evolution of the extent of attainment deficit is further illustrated by the intersection area between the attainment curve and the reference curve for three timesteps. These examples clearly demonstrate the utility of attainment deficit derived from the extended assessment framework (Figure 3B). By contrast, under the binary pass/fail approach, these three cases would be considered equal in terms of status and trends. In other words, they are always out of attainment and they all have a zero trend over time.

## APPLICATION OF THE EXTENDED FRAMEWORK

### Monitoring Data

Tidal monitoring data of DO, salinity, and temperature were obtained from the CBP Water Quality Database for the period between 1985 and 2016 (Chesapeake Bay Program, 2017). These data were collected by the Maryland (MD) Department of Natural Resources, the Virginia (VA) Department of Environmental Quality, and partners at more than 140 stations distributed across the Bay’s middle channel, tidal tributaries, and embayments. Most of these stations have been sampled consistently since 1985, at a frequency of 12–20 times per year with limited additional synoptic sampling (U.S. Environmental Protection Agency [USEPA], 2010b; Tango and Batiuk, 2013). The sampling was done using consistent sampling and analysis protocols and complemented by a rigorous quality assurance program (U.S. Environmental Protection Agency [USEPA], 2010b; Tango and Batiuk, 2013). Most of the 92 segments contain 1–3 long-term monitoring stations and some segments contain additional stations from supplemental monitoring programs such as shallow water monitoring and citizen volunteer monitoring.

### Attainment Deficit

The extended assessment framework was applied to the Chesapeake Bay segments (Figure 1) for three DO-related DUs, i.e., OW, DW, and DC (Figure 2), which resulted in estimates of attainment deficit for each applicable segment and DU for each running 3-year assessment period from 1985–1987 to 2014–2016. For this work, we focused on summer results (June-September). As previously described, estimated attainment deficit falls between 0% (i.e., all space and time are in attainment for the assessment period) and –100% (i.e., all space and time are out of attainment for the assessment period).

The segment-level estimates of attainment deficit were further aggregated for each 3-year period to investigate the status and trends with different types of subgrouping. These subgroups include three different DUs (i.e., OW, DW, and DC), four salinity zones [i.e., tidal fresh (TF), oligohaline (OH), mesohaline (MH), and polyhaline (PH)], and thirteen tidal systems. For each subgroup, all applicable segments were selected and their attainment deficit values in each assessment period were averaged through surface-area weighting:

(1) $$AD_{subgroup\;J}=\frac{\sum_{j}^{\;all\;segments\;\in \;J}AD_{j}*A_{j}}{\sum_{j}^{all\;segments\;\in \;J}A_{j}}$$

where *AD*_*j*_ is the estimated attainment deficit value and *A*_*j*_ is segment surface area for segment j within subgroup *J*. This weighting scheme was adopted for two reasons: (a) segments vary in size over four orders of magnitude (0.13–1,521 km^2^; sum = 11,600 km^2^) - see Figure 1, and (b) surface area of each segment does not change with time or DU, unlike seasonally variable bottom water area or water volume. For certain segments in a 3-year period, monitoring data might not be available to produce attainment deficit values; those segments were excluded from the summation operations in Equation (1) to minimize bias in the aggregated result of *AD* for that period and correspondingly, estimated trends in *AD*.

### Trend Analysis

Trend analysis was conducted on the estimated attainment deficit values to determine whether DO conditions have improved over time. To do this, we used a modified version of the Mann- Kendall (MK) test that can account for autocorrelation in the time series (Hamed and Rao, 1998). This non-parametric test was chosen because the attainment deficit time series is not expected to follow any specific distribution and the values are bounded between −100 and 0%. An autocorrelation correction was needed because the assessment was conducted on monitoring data in running 3-year periods. The Sen slope was computed as well to generate an estimate of change over time (Sen, 1968). The modified Mann-Kendall and the Sen slope tests were implemented through the “mkTrend” function in the R-package “fume” (Santander Meteorology Group, 2012) to calculate the significance and slope for both a long-term trend (1985–2016) and a short-term trend (2000–2016). Following Hirsch et al. (2015), the significance level of a MK trend was not restricted to 0.05 to enhance the chance of detecting appreciable changes that are worthy of management considerations. Multiple alpha levels were considered, i.e., 0.05, 0.1, and 0.25. In addition, change-point analysis was conducted to test for a shift in the central tendency of the attainment deficit time series. The non-parametric Pettitt test was adopted (Pettitt, 1979), which was implemented using the “pettitt.test” function in the R-package “trend” (Pohlert, 2018).

### Data Availability

For the convenience of readers and end users, our results of attainment deficit are provided in the online Supplementary Material, including:

(1) A table for subgroup-level attainment deficit time series for the three DUs, four salinity zones, and thirteen tidal systems - see Appendix A (Supplementary Table S2).
(2) A spreadsheet file for segment-level attainment deficit time series for each of the 92 segments for applicable DUs - see Appendix B.
(3) A PDF package for segment-level attainment deficit for each of the 92 segments for applicable DUs, accompanied by long-term and short-term MK trends - see Appendix C.

## RESULTS AND DISCUSSION

### Current Status (2014–2016) of Chesapeake Bay DO Attainment Deficit

#### Segment Patterns

**FIGURE 5 |:**
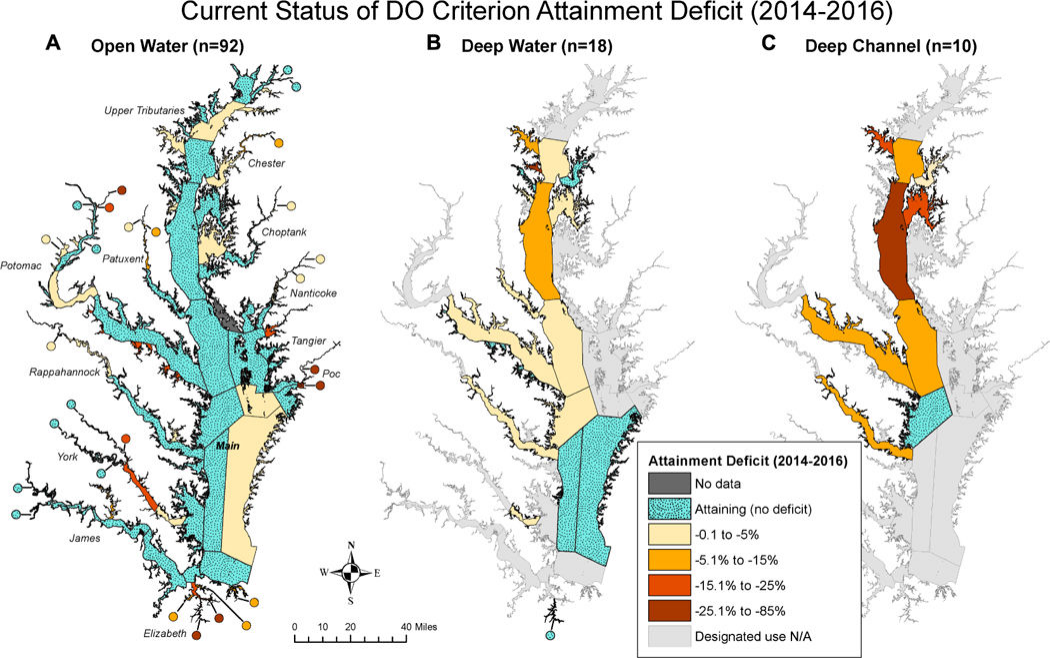
Maps showing the current status (i.e., 2014–2016 period) of estimated attainment deficit for Chesapeake Bay dissolved oxygen criterion for applicable segments for **(A)** open water, **(B)** deep water, and **(C)** deep channel.

The most recent (i.e., the 2014–2016 assessment period, hereafter referred to as “current”) status of attainment deficit for each Chesapeake Bay segment is presented in Figure 5. This result and elaborations below highlight the usefulness of the attainment deficit quantification for identifying places where patterns are different and where further evaluations are needed. Overall, there is a clear progression among the three DUs - i.e., in general, attainment status gets worse with depth as the DU goes from OW and DW to DC, which is consistent with the expectation that bottom water habitats of the tidal waters are not as healthy as surface areas in terms of DO conditions.

For OW (Figure 5A), 89 of the 92 applicable segments had data in the 2014–2016 assessment period. More than half of these segments (*n* = 48) were in full attainment in this period, including segments in the mainstem Bay and many tributaries. The status of attainment deficit was better than −4.8% for 75% of the applicable OW segments and better than −20.8% for 90% of the applicable OW segments. Overall, OW segments were dominated by zero or relatively small attainment deficit values in 2014–2016.

For DW (Figure 5B), all the 18 applicable segments had data in the 2014–2016 period. One third of these segments (*n* = 6) were in full attainment in this period, including segments in the polyhaline region of the Bay’s mainstem. The status of attainment deficit was better than −3.6% for 75% of the applicable DW segments and better than −9.7% for 90% of the applicable DW segments. The largest deficit (−28.4%) was observed within segment MAGMH *(Magothy River),* an upper western shore tributary in MD. Notably, the mainstem segment CB4MH *(Middle Central Bay)* had the second largest attainment deficit (−14.6%) among all the DW segments in this assessment period. Like OW segments, DW segments were dominated by zero or minimal attainment deficit values in 2014–2016.

For DC (Figure 5C), 9 of the 10 applicable segments had data in the 2014–2016 period. Only one segment was in full attainment in this period - i.e., CB5MH_VA *(Lower Central Bay, VA).* The status of attainment deficit was better than –15.4% for 75% of the applicable DC segments and better than –22.8% for 90% of the applicable DC segments. The largest deficit was observed with segment CB4MH, which was −40.5%. This is not surprising, since CB4MH is the region of the Bay where annual summer hypoxia develops first and lasts the longest (Testa and Kemp, 2014; Testa et al., 2018). Overall, DC segments had more occurrences of moderate or large attainment deficit in 2014–2016, as compared with OW and DW.

#### Subgroup Patterns

The segment-level attainment deficit was aggregated into different subgroups using Equation 1 based on the segments’ DU, salinity zone, or tidal systems. The initial and most recent attainment deficit values calculated for each of these subgroups are provided in Table 1 (For the complete time series, see Supplementary Table S2). For the three DUs, OW, DW, and DC had aggregated attainment deficit values of −0.8%, −3.2%, and −15.2%, respectively, in the 2014–2016 assessment period. This is consistent with the expectation that DC segments had generally poorer conditions than OW and DW segments.

For the four salinity zones, the 2014–2016 attainment deficit results exhibited the following ranking: PH (−0.2%) > TF (−1.4%) > OH (−1.6%) > MH (−6.0%). MH segments are generally subject to strong interactions between landward and seaward flows, which result in strong summer stratification that can prevent replenishment of oxygen from the water surface, exacerbating eutrophication effects. By contrast, TF and OH segments are generally more dominated by freshwater flow and hence less susceptible to stratification and more frequently replenished with DO-rich fresh waters. PH segments are closer to relatively DO-rich oceanic waters and tend to mix vertically in the late summer earlier than MH segments, resulting in their near-attainment status.

For the thirteen tidal systems, near-attainment status was achieved by Nanticoke (−0.3%), James (−0.3%), Choptank (−0.5%), and Tangier (−0.7%). Attainment deficit was better than −5% in Chester, Patuxent, Potomac, Rappahannock, Pocomoke Rivers, and the mainstem Bay. Attainment deficit was between −6 and −10% in upper mainstem Bay tributaries and York River. Elizabeth River is the only tidal system with a deficit worse than −10% in 2014–2016 (−22.6%).

**FIGURE 6 |:**
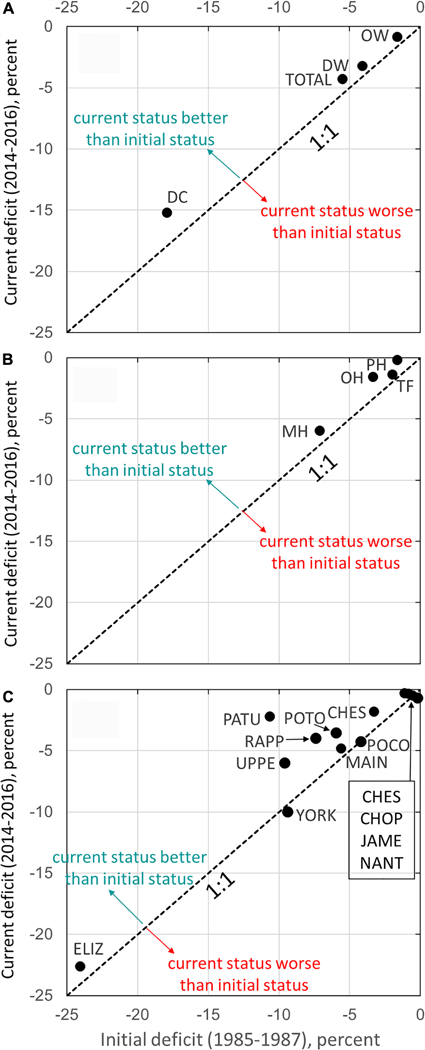
Estimated attainment deficit results for Chesapeake Bay dissolved oxygen criterion by **(A)** designated use, **(B)** salinity zone, and **(C)** tidal system, comparing the current status (i.e., 2014–2016) and initial status (i.e., 1985–1987).

How has the status of Chesapeake Bay’s DO criterion attainment changed over time? For brevity, this question was addressed by aggregating individual segments into groups by designated use, by salinity zone, and by tidal system. Each subgroup’s current aggregated attainment deficit (2014–2016) was then plotted against its initial attainment deficit (1985–1987) (Figure 6). The current status of each DU (Figure 6A) is better than its initial condition, with moderate improvements ranging between 0.8 and 2.8%. Similarly, the 2014–2016 condition of each salinity zone (Figure 6B) is better than its initial status, with moderate improvements ranging between 0.6 and 1.8%. The majority of tidal systems (Figure 6C) have better or similar current status compared to initial status. Notably, Patuxent River showed a substantial improvement of 8.4%. The Rappahannock, upper mainstem Bay tributaries, Potomac, Chester, and Elizabeth Rivers had moderate improvements in the range of 1.5~3.6%. The mainstem Bay, Nanticoke, James, and Choptank rivers showed improvements of <1%. The York, Tangier, and Pocomoke systems were the only subgroups that showed degradation in DO attainment from 1985–1987 to 2014–2016, but these differences were almost negligible (within <1%).

### Decadal Trends in Chesapeake Bay DO Attainment Deficit

#### Segment Patterns

**FIGURE 7 |:**
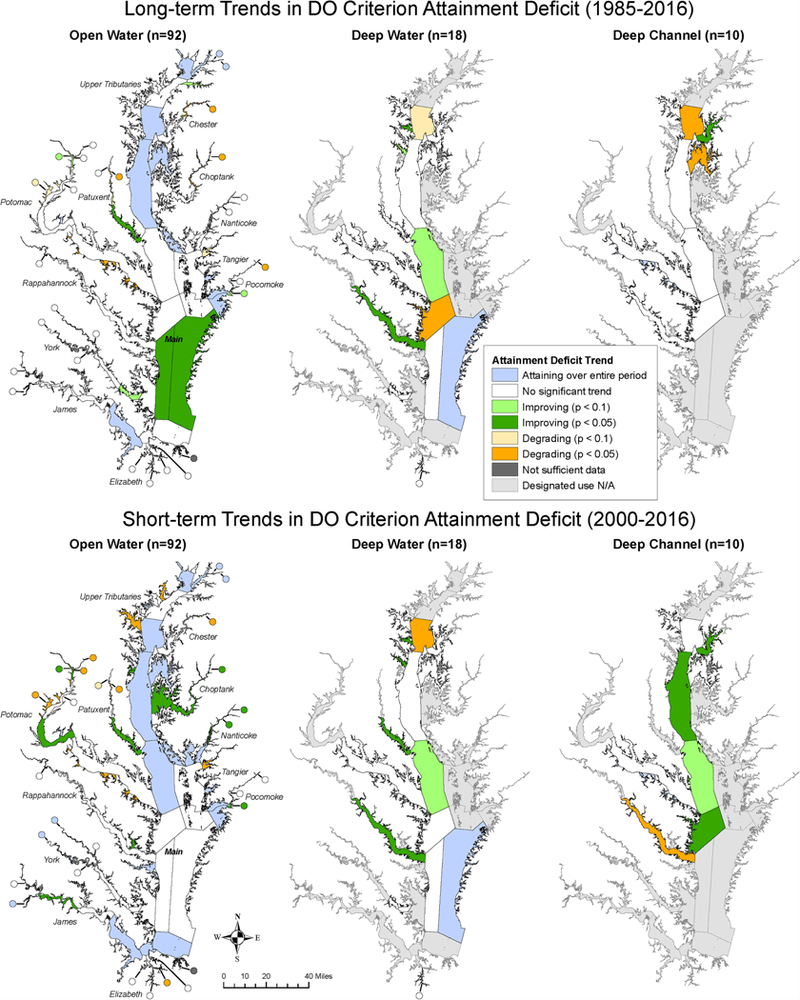
Maps showing long-term **(Top)** and short-term **(bottom)** trends in estimated attainment deficit for Chesapeake Bay dissolved oxygen criterion for applicable segments for open water, deep water, and deep channel.

The long-term (1985–2016) and short-term (2000–2016) trends in attainment deficit for Chesapeake Bay segments show strong spatial variations (Figure 7). The number of segments with improving and degrading trends are summarized in Table 2. Below, we elaborate on these trends and for brevity we focus on trends with *p <* 0.1. These results highlight the effectiveness of using attainment deficit for identifying places that are associated with improving (or degrading) trends. Such information can help guide targeting of management strategies and research to explain trend trajectories.

Among OW segments, seven had improving long-term trends and 15 segments showed improving short-term trends. However, only three segments showed consistently improving trajectories for both long-term and short-term trends, which are PAXMH *(Lower Patuxent River),* POCOH_VA *(Middle Pocomoke River, VA),* and POTTF_DC *(Upper Potomac River, DC).* The remaining four segments with long-term improving trends – CB6PH *(Western Lower Bay),* CB7PH *(Eastern Lower Bay),* SASOH *(Sassafras River),* and YRKPH *(Lower York River)* - showed no significant short-term trend. Of the remaining 12 segments with improving short-term OW trends, 10 showed no significant long-term OW trend. These included the lower portion of the Choptank river (CHOMH1 and CHOMH2), the Corrotoman River (CRRMH), one tidal-fresh segment of the James River (JMSTF1), one oligohaline segment of the Potomac River (POTOH1_MD), all but the lowest portion of the Nanticoke River (NANOH, NANTF_DE, NANTF_MD), as well as the mesohaline portions of the Rhode and West Rivers (RHDMH and WSTMH). Two segments (CHOOH and CHOTF; both in the Choptank river) with recent improving OW trends still had degrading long-term OW trend, indicating that in spite of recent improvements, conditions are still more degraded than they were in the mid-1980s. Eight of the remaining segments showed long-term degrading OW trends. Five of these segments, namely, CHSTF *(Upper Chester River),* PAXTF *(Upper Patuxent River),* POTMH_VA *(Lower Potomac River, VA*), POTTF_VA *(Upper Potomac River, VA*), and WICMH *(Wicomico River*), showed also degrading trends in the short-term period. Moreover, additional six segments with no significant long-term OW trend showed recent degradation (ANATF_DC, ANATF_MD, BSHOH, EBEMH, PATMH, and WBRTF).

Among DW segments, four had improving long-term trends, namely, CB5MH_MD *(Lower Central Bay, MD),* MAGMH *(Magothy River),* RPPMH *(Lower Rappahannock River),* and SOUMH *(South River*). For the short-term trend, improving conditions were also observed in these four segments, in addition to PAXMH *(Lower Patuxent River).* By contrast, degrading trends were associated with two segments (CB3MH, *Upper Central Bay*; CB5MH_VA, *Lower Central Bay, VA*) for the long-term period and one segment (CB3MH) for the short-term period, both of which are located in the mainstem of the Bay.

Among DC segments, only one had improving long-term trend, i.e., CHSMH *(Lower Chester River*). For the short-term trend, three mainstem segments in addition to CHSMH showed improving conditions, namely, CB4MH *(Middle Central Bay),* CB5MH_MD *(Lower Central Bay, MD),* and CB5MH_VA *(Lower Central Bay, VA).* By contrast, degrading trends were associated with two segments (CB3MH; EASMH, *Eastern Bay)* for the long-term period and one segment (RPPMH; *Lower Rappahannock River*) for the short-term period.

Overall, the results show that many segment-DU pairs did not have significant, improving trends, suggesting that continued implementation of pollution management practices will be necessary to attain DO criterion. Further evaluation of the DO observations outside of the attainment assessment framework could very likely uncover additional trends, especially if the space-time exceedance of the DO criterion has changed in such a way that the overall attainment deficit has not changed (e.g., improvements in one part of the summer and not another). In addition, greater data resolution in space and time may provide more robust details of spatial conditions that can reduce uncertainty in assigning status and enhance the power to detect trends through time. Nonetheless, some significant trends were detected based on the metric of attainment deficit. Particularly included are some mainstem DC segments for the short-term period - i.e., CB4MH, CB5MH_MD, and CB5MH_VA, which are in the region of historically low summer DO (Hagy et al., 2004) and present promising evidence on the ecosystem recovery for portions of Chesapeake Bay with respect to DO criterion attainment.

#### Subgroup Patterns

**FIGURE 8 |:**
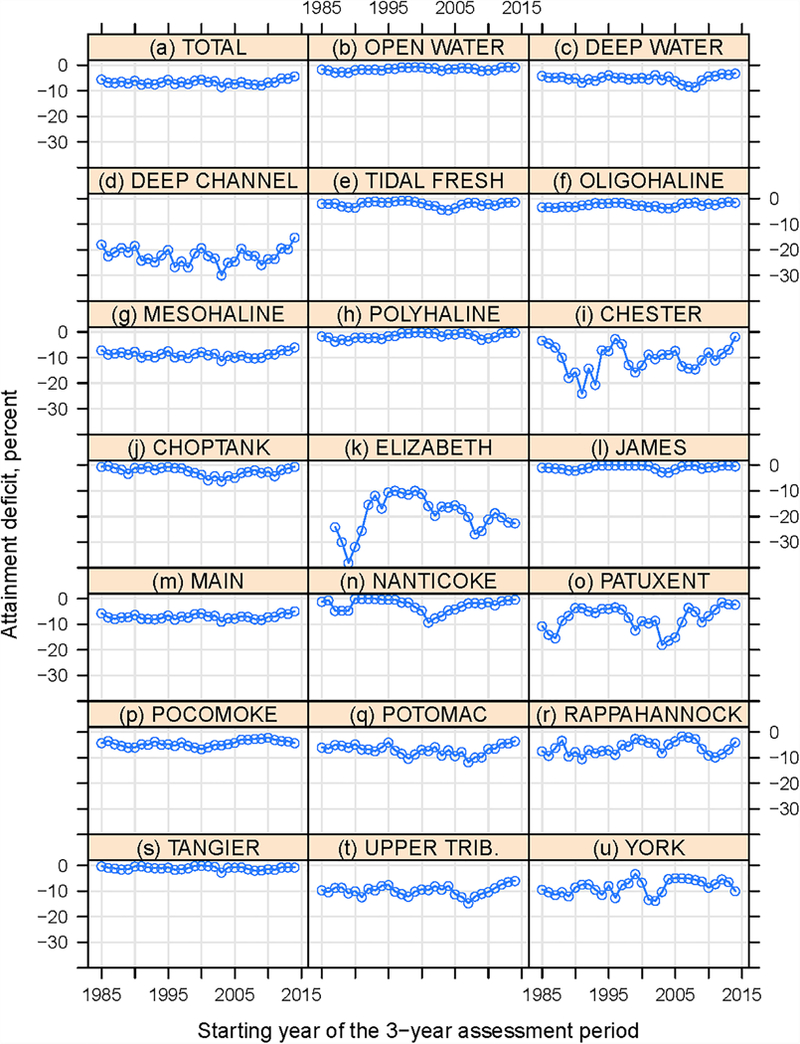
Estimated attainment deficit results for Chesapeake Bay dissolved oxygen criterion by **(a-d)** designated use, **(e-h)** salinity zone, and **(i—u)** tidal system. Refer to Supplementary Table S2 for annual values.

Time series of estimated attainment deficit for the subgroups are plotted in Figure 8. Trend results are summarized in Table 1. Among the three DUs (Figures 8b-d), only OW showed a statistically significant long-term trend with a slope of 0.04 percent/year. It was detected to have a change point at the 3-year period of 1994–1996, which is consistent with the previously identified shift in Chesapeake Bay water quality attainment indicator (Zhang et al., 2018). For DW and DC, neither the long-term nor short-term trend was statistically significant. However, their short-term trends were notable in magnitude – i.e., 0.13 and 0.24 percent/year, respectively. Also notable is the consistent and steady improvements in conditions since around 2009–2011 in OW and especially DW and DC.

Among the four salinity zones (Figures 8e-h), the TF zone had a negligible long-term trend and a positive short-term trend, but neither was statistically significant. MH trends behaved similarly to those in the TF zone in terms of slope and significance. By contrast, the OH zone had positive and statistically significant trends for both the long-term and short-term periods. More research is needed to test whether these improvements might be related to reductions of nutrient loads from tributaries or related to more short-term variations in hydrology. OH segments with improving trends are in the Choptank, Nanticoke, Pocomoke, Potomac, and Sassafras rivers (Figure 1). Finally, the PH zone had a positive and statistically significant long-term trend but a negligible short-term trend.

Among the 13 tidal systems (Figures 8i-u), only York had a statistically significant long-term trend, i.e., 0.15 percent/year, although it is one of the systems that showed degradation when just the 1985–1987 period was compared to the most recent period (Figure 6C). This disconnect appears to be due to a dip in the attainment deficit value in the last period (Figure 8u). An examination of the segment-level trends for York revealed that this long-term overall improvement was driven by an improvement in the OW attainment condition of the YRKPH segment *(Lower York River),* which has a long-term trend of 0.26 percent/yr (*p* < 0.1) (see Appendix C). More subgroups showed statistically significant short-term trends, including Choptank, James, Nanticoke, Patuxent, and Pocomoke (positive trends) and Elizabeth (negative trend), which can be attributed to specific segment-DU combinations shown in Appendix C. Of these tidal systems, the Patuxent had the largest short-term improvement – its aggregated attainment condition has improved with a slope of 0.98 percent/year over the short-term period. This pattern was driven by improvements in the PAXMH (mesohaline) and PAXOH (oligohaline) segments, although attainment conditions of the two tidal fresh segments (PAXTF and WBRTF) actually degraded. Another interesting case is the Nanticoke, where the short-term improvement was driven by rapidly improving conditions *(p* < 0.05) in the three OH and TF segments (i.e., NANOH, NANTF_DE, and NANTF_MD) but with no trends in the MH segment (NANMH). While attainment trends were different among salinity zones for the two systems above, the Choptank presents an example where the aggregated attainment condition represented improvements in segments distributed across all salinity zones, including CHOMH1, CHOMH2, CHOOH, and CHOTF. The Elizabeth presents a sharp contrast to the above tidal systems; attainment has degraded here in the last short-term period with a slope of - 0.53 percent/year. This pattern was driven by downward trends in the OW attainment condition of EBEMH *(Eastern Branch Elizabeth River*), ELIPH *(Mouth to mid-Elizabeth River*), and WBEMH *(Western Branch Elizabeth River*), although only the EBEMH trend was statistically significant.

Overall, these subgroup trend results corroborate the segment trend results discussed above. Several significant, improving trends present promising evidence on the recovery for portions of Chesapeake Bay with respect to DO criterion attainment. However, these improvements are generally limited in magnitude (see Table 1). Overall, the general lack of significantly improving trends across the Bay over the long-term (1985–2016) and short-term (2000–2016) periods suggests that further actions will be necessary to achieve full attainment of DO criterion.

## CONCLUSION

We have introduced an analytical extension of the Chesapeake Bay water quality criterion assessment framework for quantifying the amount of space-time exceedance of DO criterion for a specific segment (“attainment deficit”) and have demonstrated the usefulness of this framework by applying it to evaluate water-quality changes in the Chesapeake Bay ecosystem. With this approach, a comprehensive assessment of DO criterion attainment was conducted for Bay segments for each running 3-year period in 1985–2016. In general, the current status of attainment deficit (i.e., 2014–2016) is considerably worse for DC segments than OW and DW segments. Most subgroups show better (or similar) attainment status in 2014–2016 than their initial status (1985–1987). In terms of decadal trends, some significant trends (*p* < 0.1) were detected, presenting evidence on the recovery for portions of Chesapeake Bay with respect to DO criterion attainment. Over the 30 3-year periods in 1985–2016, significant, improving trends were observed in seven OW segments, four DW segments, and one DC segment. Over the recent 15 3-year periods (2000–2016), significant, improving trends were observed in 15 OW segments, five DW segments, and four DC segments. Subgroups showed mixed trends, with Patuxent, Nanticoke, and Choptank Rivers experiencing significant, improving short-term trends while Elizabeth experiencing a significant, degrading short-term trend. The general lack of significantly improving trends across the Bay suggests that further actions will be necessary to achieve full attainment of DO criterion. Overall, these attainment deficit results provided detailed information regarding the status and trends of DO criterion attainment in Chesapeake Bay that can help target areas for further evaluation or refined management plans. Enhanced details for changes in habitat conditions are critical to the management and research community for understanding the conditions and dynamics of the Bay ecosystem and for further assessing the effectiveness of management initiatives aimed toward Bay restoration. More broadly, this work features Chesapeake Bay as an example where long-term monitoring data and science-based criterion assessment methods can be combined to evaluate complex ecosystems.

There are several directions for future research. First, the assessment can benefit from continued water quality monitoring as well as the promotion of new monitoring initiatives, such as volunteer monitoring and non-traditional partner contributions to increase station data densities as well as *in situ,* high resolution DO measurements. Second, the assessment approach is subject to limitations of data availability and key assumptions made to accommodate those limitations. Future work should incorporate new methods and further validate such types of assumptions to better understand short-term variability and evaluate the sensitivity of the results (particularly decadal trends) to such limitations. Third, new research should be done to tease apart the space and time aspects of the attainment deficit, so that improving or degrading trends can be more properly understood and communicated. Fourth, the segment-based attainment assessment results can be compared with station-level DO trends. Researchers in the CBP partnership have implemented a generalized additive model (GAM) statistical approach to assess tidal-station trends. Comparison between the attainment deficit results and GAM trends to look for similarity (or dissimilarity) may provide new insights into the attainment deficit patterns as well as a deeper understanding of how and when water-quality improvements result in criteria attainment. Last but not least, clear, significant linkages between attainment status in the various segments and drivers, such as management actions (e.g., reduction of nutrient loads), internal hydrodynamic characteristics, trophic interactions, and climatic and hydrological variability, remain elusive. The relation of temporal and spatial patterns of these drivers (among others) to DO criteria attainment warrants further investigation.

## Supplementary Material

Supplementary A

Supplementary B

Supplementary C

## Figures and Tables

**TABLE 1 |:** Estimated attainment deficit (initial and current status) and associated statistical results^a^ for Chesapeake Bay dissolved oxygen criterion for the three designated uses, four salinity zones, and thirteen tidal systems.

Subgroup	Initial deficit (1985–1987), percent	Current deficit (2014–2016), percent	Change point (3-year period)	30-cycle trend, percent/year	15-cycle trend, percent/year

All segments and DUs (TOTAL)	−5.5	−4.3	2010–2012 -	0.01 -	0.10 -
***Designated use***					
Open water (OW)	−1.6	−0.8	1994–1996 ***	0.04 *	0.02 -
Deep water (DW)	−4.1	−3.2	2009–2011 -	0.00 -	0.13 -
Deep channel (DC)	−17.9	−15.2	1990–1992 -	−0.03 -	0.24 -
***Salinity zone***					
Tidal fresh (TF)	−2.0	−1.4	2000–2002 -	−0.01 -	0.13 -
Oligohaline (OH)	−3.3	−1.6	2005–2007 *	0.04 *	0.15 ***
Mesohaline (MH)	−7.1	−6.0	2009–2011 -	−0.01 -	0.14 -
Polyhaline (PH)	−1.6	−0.2	1995–1997 ***	0.07 *	−0.03 -
***Tidal system***					
Chester (CHES)	−3.3	−1.8	1987–1989 -	0.06 -	0.16 -
Choptank (CHOP)	−0.5	−0.5	1997–1999 ***	−0.07 -	0.31 ***
Elizabeth (ELIZ)^b^	−24.1	−22.6	1991–1993 *	−0.24 -	−0.53 ***
James (JAME)	−0.8	−0.3	1992–1994 -	0.00 -	0.07 *
Mainstem Bay (MAIN)	−5.6	−4.8	2009–2011 -	0.02 -	0.09 -
Nanticoke (NANT)	−1.1	−0.3	1998–2000 *	−0.01 -	0.55 ***
Patuxent (PATU)	−10.7	−2.2	2006–2008 -	0.08 -	0.98 ***
Pocomoke (POCO)	−4.2	−4.2	2003–2005 ***	0.07 -	0.23 *
Potomac (POTO)	−6.0	−3.5	1995–1997 -	−0.07 -	0.19 -
Rappahannock (RAPP)	−7.4	−3.9	1996–1998 *	0.10 -	−0.29 -
Tangier (TANG)	−0.2	−0.7	2002–2004 -	−0.01 -	−0.05 -
Upper mainstem Bay tributaries (UPPE)	−9.6	−6.0	2010–2012 -	0.03 -	0.17 -
York (YORK)	−9.4	−10.0	2003–2005 ***	0.15 ***	0.04 -

a Significance levels are provided next to each estimate:

(***) *p* < 0.05

(*) 0.05 < *p* < 0.1

(-) *p* > 0.1.

b Elizabeth does not have data in 1985–1987 or 1986–1988, so the earliest period with data (i.e., 1987–1989) was used to represent its initial deficit status.

**TABLE 2 |:** Summary of segments with improving and degrading trends in estimated attainment deficit for Chesapeake Bay dissolved oxygen criterion in the long-term period (1985–2016) and short-term period (2000–2016).

DU	Trend	Period	*p* < 0.05	*p* < 0.1	*p* < 0.25	*p* < 1.0	Segments with *p* < 0.1

OW (*n* = 92)	Improving	1985–2016	4	7	12	18	CB6PH; CB7PH; PAXMH; POCOH_VA; POTTF_DC; SASOH; YRKPH
	Improving	2000–2016	15	15	18	25	CHOMH1; CHOMH2; CHOOH; CHOTF; CRRMH; JMSTF1; NANOH; NANTF_DE; NANTF_MD; PAXMH; POCOH_VA; POTOH1_MD; POTTF_DC; RHDMH; WSTMH
	Degrading	1985–2016	8	10	11	23	CHOOH; CHOTF; CHSOH; CHSTF; PAXOH; PAXTF; POCTF; POTMH_VA; POTTF_VA; WICMH
	Degrading	2000–2016	10	11	12	17	ANATF_DC; ANATF_MD; BSHOH; CHSTF; EBEMH; PATMH; PAXTF; POTMH_VA; POTTF_VA; WBRTF; WICMH
DW (*n* = 18)	Improving	1985–2016	2	4	5	7	CB5MH_MD; MAGMH; RPPMH; SOUMH
	Improving	2000–2016	4	5	6	10	CB5MH_MD; MAGMH; PAXMH; RPPMH; SOUMH
	Degrading	1985–2016	1	2	2	7	CB3MH; CB5MH_VA
	Degrading	2000–2016	1	1	2	3	CB3MH
DC *(n* = 10)	Improving	1985–2016	1	1	2	4	CHSMH
	Improving	2000–2016	3	4	4	7	CB4MH; CB5MH_MD; CB5MH_VA; CHSMH
	Degrading	1985–2016	2	2	2	5	CB3MH; EASMH
	Degrading	2000–2016	1	1	1	2	RPPMH

## Footnotes

SUPPLEMENTARY MATERIAL
