# Supplementary material for: Chesapeake Bay Dissolved Oxygen Criterion Attainment Deficit: Three Decades of Temporal and Spatial Patterns
Source: Front Mar Sci. Author manuscript; Available in PMC 2019 Sep 18. (PMC6750769; doi:10.3389/fmars.2018.00422)
Supplement: Supplementary A [file NIHMS1042326-supplement-Supplementary_A.docx]

Supplementary Material

Chesapeake Bay Dissolved Oxygen Criterion Attainment Deficit: Three Decades of Temporal and Spatial Patterns

Qian Zhang^1*^, Peter J. Tango^2^, Rebecca R. Murphy^1^, Melinda K. Forsyth^3^, Richard Tian^1^, Jennifer Keisman^4^, and Emily M. Trentacoste^5^

^1^ Chesapeake Bay Program Office, University of Maryland Center for Environmental Science, Annapolis, MD, USA

^2^ Chesapeake Bay Program Office, U.S. Geological Survey, Annapolis, MD, USA

^3^ Chesapeake Biological Laboratory, University of Maryland Center for Environmental Science, Solomons, MD, USA

^4^ Maryland-Delaware-District of Columbia Water Science Center, U.S. Geological Survey, Catonsville, MD, USA

^5^ Chesapeake Bay Program Office, U.S. Environmental Protection Agency, Annapolis, MD, USA

*** Correspondence:** Qian Zhang ([qzhang@chesapeakebay.net](mailto:qzhang@chesapeakebay.net))

| **Table S1.** Water quality criteria for dissolved oxygen, water clarity/underwater bay grasses, and chlorophyll-a and the designated uses (USEPA, 2003b; USEPA, 2003a; USEPA, 2004b). Criterion thresholds that were used in the dissolved oxygen criterion attainment assessment are shown in blue color. Red color highlights the three designated uses considered in this study. | | | | | | |
| --- | --- | --- | --- | --- | --- | --- |
| Criterion | Designated Use | Season | Threshold | Critical Value | Applicable Segments | Designation in this paper |
| Dissolved Oxygen (DO) | Migratory fish spawning and nursery (MSN) | February 1 - May 31 | 7-day mean | 6 mg L^-1^ | 73 | Not Applicable |
|  |  | February 1 - May 31 | Instantaneous | 5 mg L^-1^ |  |  |
|  |  | June 1 - January 31 | *OW criteria apply* | |  |  |
|  | Open-water fish and shellfish (OW) | Year-round | 30-day mean ^b^ | 5.5 mg L^-1^ in very low salinity; 5 mg L^-1^ otherwise ^c^ | 92 | OW |
|  |  |  | 7-day mean | 4 mg L^-1^ |  |  |
|  |  |  | Instantaneous | 3.2 mg L^-1^ |  |  |
|  | Deep-water seasonal fish and shellfish (DW) | June 1 - September 30 | 30-day mean | 3 mg L^-1^ | 18 | DW |
|  |  | June 1 - September 30 | 1-day mean | 2.3 mg L^-1^ |  |  |
|  |  | June 1 - September 30 | Instantaneous | 1.7 mg L^-1^ |  |  |
|  |  | October 1 - May 31 | *OW criteria apply* | |  |  |
|  | Deep-channel seasonal refuge (DC) | June 1 - September 30 | Instantaneous | 1 mg L^-1^ | 10 | DC |
|  |  | October 1 - May 31 | *OW criteria apply* | |  |  |
|  | Shallow-water bay grass (SW) | June 1 - September 30 | *Dependent upon OW attainment assessment* | | 79 | Not Applicable |
| Chlorophyll-a | Open-water fish and shellfish (OW) | Spring  (March 1 - May 31) | 10 to 15 μg L^-1^ (salinity based) | | 7 | Not Applicable |
|  |  | Summer  (July 1 - September 30) | 10 to 25 μg L^-1^ (salinity based) | |  | Not Applicable |
| SAV and/or Water Clarity | Shallow-water bay grass (SW) | SAV season | Segment specific water clarity and bay grass acreage goals | | 79 | Not Applicable |
| ^a^ USEPA (2003) does not have a 30-day mean February-May threshold for MSN. The decision for attainment assessment used a 30-day mean of 6 mg L^-1^ as February-May threshold, same as the 7-day mean threshold.  ^b^ June-September (as opposed to the entire year) is evaluated for the 30-day mean criterion for OW in the attainment assessment procedures.  ^c^ Critical value has been adjusted from 5 mg L^-1^ to 4 mg L^-1^ for segments MPNTF, MPNOH, PMKTF, and PMKOH to account for water quality criterion variances. | | | | | | |

| **Table S2**. Estimated attainment deficit results (in percent) for Chesapeake Bay dissolved oxygen criterion (“Total”) and associated results for each designated use, salinity zone, and tidal system. Refer to Table 1 for abbreviation details. | | | | | | | | | | | | |
| --- | --- | --- | --- | --- | --- | --- | --- | --- | --- | --- | --- | --- |
| Period | TOTAL | Designated Use | | | Salinity Zone | | | | Tidal System | | |  |
|  |  | OW | DW | DC | TF | OH | MH | PH | CHES | CHOP | ELIZ | |
| 1985-1987 | -5.51 | -1.63 | -4.12 | -17.93 | -1.97 | -3.34 | -7.12 | -1.65 | -3.29 | -0.53 | NA | |
| 1986-1988 | -6.75 | -1.96 | -4.77 | -22.46 | -2.02 | -3.34 | -8.77 | -2.02 | -4.45 | -0.13 | NA | |
| 1987-1989 | -6.91 | -2.85 | -4.81 | -20.95 | -1.86 | -3.58 | -8.40 | -3.68 | -5.98 | -1.08 | -24.05 | |
| 1988-1990 | -6.40 | -2.64 | -4.56 | -19.23 | -2.98 | -3.12 | -7.93 | -2.93 | -9.95 | -1.59 | -29.90 | |
| 1989-1991 | -7.10 | -2.88 | -5.38 | -20.92 | -3.36 | -3.16 | -8.74 | -3.42 | -17.91 | -3.25 | -38.08 | |
| 1990-1992 | -6.00 | -1.85 | -5.06 | -18.43 | -3.52 | -3.23 | -7.61 | -2.13 | -15.72 | -0.96 | -31.75 | |
| 1991-1993 | -7.55 | -1.63 | -6.87 | -24.18 | -1.52 | -2.55 | -9.93 | -2.13 | -24.06 | -1.32 | -25.47 | |
| 1992-1994 | -7.04 | -1.77 | -5.46 | -23.43 | -1.37 | -2.44 | -9.14 | -2.36 | -14.25 | -0.56 | -15.35 | |
| 1993-1995 | -7.44 | -1.67 | -6.04 | -24.85 | -1.03 | -1.69 | -9.85 | -2.12 | -20.59 | -1.72 | -11.82 | |
| 1994-1996 | -6.67 | -2.01 | -4.67 | -22.09 | -1.48 | -1.99 | -8.52 | -2.64 | -7.09 | -0.90 | -16.81 | |
| 1995-1997 | -5.65 | -1.26 | -3.87 | -20.02 | -1.48 | -1.81 | -7.46 | -1.51 | -7.41 | -0.53 | -10.51 | |
| 1996-1998 | -7.30 | -1.38 | -4.90 | -26.68 | -0.99 | -1.54 | -9.88 | -1.48 | -2.69 | -0.98 | -9.84 | |
| 1997-1999 | -6.51 | -0.73 | -4.77 | -24.48 | -0.75 | -1.65 | -9.13 | -0.39 | -4.62 | -1.04 | -10.78 | |
| 1998-2000 | -7.27 | -0.91 | -5.47 | -26.86 | -0.79 | -2.04 | -10.15 | -0.53 | -12.78 | -2.26 | -11.38 | |
| 1999-2001 | -5.99 | -0.70 | -5.11 | -21.33 | -1.21 | -2.70 | -8.42 | -0.08 | -15.73 | -2.85 | -9.85 | |
| 2000-2002 | -5.59 | -0.77 | -4.94 | -19.30 | -1.76 | -2.70 | -7.79 | -0.17 | -12.98 | -3.56 | -11.06 | |
| 2001-2003 | -6.54 | -1.18 | -5.46 | -22.34 | -2.57 | -3.25 | -9.00 | -0.45 | -8.77 | -5.77 | -15.80 | |
| 2002-2004 | -6.13 | -1.11 | -3.79 | -23.29 | -2.82 | -2.89 | -8.42 | -0.46 | -10.52 | -4.15 | -19.68 | |
| 2003-2005 | -8.47 | -2.11 | -5.69 | -29.93 | -4.33 | -3.65 | -11.27 | -1.67 | -8.75 | -6.18 | -16.04 | |
| 2004-2006 | -6.88 | -1.46 | -4.39 | -25.02 | -4.49 | -3.79 | -9.25 | -0.82 | -8.85 | -4.06 | -16.36 | |
| 2005-2007 | -7.35 | -1.49 | -6.23 | -24.53 | -3.74 | -3.39 | -9.96 | -0.90 | -7.32 | -4.84 | -15.53 | |
| 2006-2008 | -6.53 | -0.96 | -7.65 | -19.53 | -2.32 | -1.92 | -9.10 | -0.31 | -13.29 | -2.89 | -17.08 | |
| 2007-2009 | -7.31 | -1.12 | -8.23 | -22.22 | -1.63 | -1.83 | -10.09 | -0.73 | -14.18 | -2.52 | -20.08 | |
| 2008-2010 | -7.55 | -1.38 | -8.53 | -22.33 | -1.50 | -1.48 | -10.25 | -1.37 | -14.53 | -1.85 | -26.83 | |
| 2009-2011 | -7.84 | -2.22 | -5.80 | -25.91 | -2.70 | -2.71 | -9.99 | -2.97 | -10.99 | -2.90 | -25.59 | |
| 2010-2012 | -6.73 | -1.90 | -4.32 | -23.55 | -2.08 | -1.97 | -8.68 | -2.38 | -7.98 | -2.49 | -21.08 | |
| 2011-2013 | -6.67 | -1.86 | -4.16 | -23.56 | -2.63 | -2.44 | -8.70 | -1.98 | -11.05 | -4.15 | -18.65 | |
| 2012-2014 | -5.09 | -0.78 | -3.33 | -19.39 | -1.71 | -1.62 | -7.02 | -0.46 | -8.45 | -1.66 | -20.25 | |
| 2013-2015 | -5.22 | -0.70 | -3.71 | -19.73 | -1.50 | -1.24 | -7.32 | -0.27 | -6.86 | -1.23 | -22.24 | |
| 2014-2016 | -4.27 | -0.83 | -3.21 | -15.18 | -1.37 | -1.58 | -5.96 | -0.20 | -1.82 | -0.51 | -22.61 | |
| Period | Tidal System (continued from above) | | | | | | | | | |  | |
|  | JAME | MAIN | NANT | PATU | POCO | POTO | RAPP | TANG | UPPE | YORK |  | |
| 1985-1987 | -0.85 | -5.63 | -1.13 | -10.66 | -4.23 | -5.98 | -7.41 | -0.22 | -9.60 | -9.39 |  | |
| 1986-1988 | -0.87 | -7.20 | -0.55 | -14.02 | -3.39 | -6.42 | -9.21 | -0.84 | -10.39 | -10.35 |  | |
| 1987-1989 | -1.17 | -7.89 | -4.69 | -15.38 | -4.67 | -4.94 | -6.09 | -1.08 | -8.63 | -11.44 |  | |
| 1988-1990 | -1.29 | -7.18 | -4.59 | -8.54 | -5.31 | -5.21 | -3.29 | -1.48 | -8.59 | -10.34 |  | |
| 1989-1991 | -1.88 | -7.15 | -4.59 | -6.59 | -5.96 | -5.96 | -9.45 | -1.43 | -10.80 | -12.00 |  | |
| 1990-1992 | -2.08 | -6.21 | 0.00 | -3.53 | -5.96 | -4.73 | -7.68 | -0.27 | -9.95 | -8.52 |  | |
| 1991-1993 | -1.35 | -7.75 | 0.00 | -3.56 | -4.67 | -6.73 | -10.50 | -0.19 | -12.32 | -7.39 |  | |
| 1992-1994 | -0.96 | -7.82 | 0.00 | -4.83 | -4.89 | -6.68 | -6.92 | -0.74 | -9.00 | -7.33 |  | |
| 1993-1995 | 0.00 | -7.96 | 0.00 | -5.42 | -3.60 | -7.38 | -8.17 | -0.97 | -9.69 | -9.41 |  | |
| 1994-1996 | 0.00 | -7.56 | -0.32 | -3.86 | -4.89 | -5.96 | -7.29 | -1.06 | -7.88 | -11.44 |  | |
| 1995-1997 | 0.00 | -6.48 | -0.32 | -3.90 | -4.67 | -3.99 | -7.02 | -0.73 | -7.55 | -7.89 |  | |
| 1996-1998 | 0.00 | -8.08 | -0.32 | -3.28 | -5.31 | -7.17 | -8.76 | -1.62 | -10.20 | -12.60 |  | |
| 1997-1999 | 0.00 | -6.85 | -1.39 | -4.14 | -4.02 | -8.35 | -4.93 | -1.38 | -11.07 | -7.52 |  | |
| 1998-2000 | 0.00 | -7.25 | -1.39 | -7.28 | -5.31 | -10.35 | -5.49 | -1.01 | -12.13 | -6.86 |  | |
| 1999-2001 | 0.00 | -5.89 | -3.34 | -12.32 | -5.96 | -8.62 | -2.54 | -0.13 | -10.11 | -3.26 |  | |
| 2000-2002 | 0.00 | -5.67 | -4.61 | -8.62 | -6.62 | -6.83 | -2.97 | 0.00 | -9.31 | -6.61 |  | |
| 2001-2003 | -0.09 | -6.85 | -9.21 | -9.53 | -5.90 | -7.29 | -4.10 | -0.16 | -9.54 | -13.34 |  | |
| 2002-2004 | -1.23 | -6.57 | -7.57 | -8.54 | -4.94 | -5.79 | -4.47 | -0.37 | -8.03 | -13.76 |  | |
| 2003-2005 | -2.64 | -8.93 | -6.70 | -18.02 | -5.13 | -9.09 | -8.17 | -2.71 | -9.40 | -10.25 |  | |
| 2004-2006 | -2.77 | -7.53 | -4.45 | -16.31 | -4.62 | -7.03 | -4.69 | -0.75 | -7.90 | -5.22 |  | |
| 2005-2007 | -1.48 | -7.70 | -4.06 | -15.04 | -4.11 | -9.28 | -3.52 | -0.76 | -11.19 | -4.84 |  | |
| 2006-2008 | -0.41 | -6.86 | -2.98 | -9.04 | -2.84 | -7.66 | -1.55 | -0.62 | -12.27 | -4.90 |  | |
| 2007-2009 | -0.05 | -7.02 | -1.74 | -3.47 | -2.91 | -11.70 | -1.95 | -1.39 | -14.69 | -5.09 |  | |
| 2008-2010 | 0.00 | -7.95 | -1.62 | -5.01 | -2.59 | -9.87 | -2.57 | -1.91 | -12.05 | -5.58 |  | |
| 2009-2011 | -1.19 | -8.20 | -1.96 | -9.08 | -2.50 | -9.76 | -6.50 | -1.84 | -11.01 | -6.16 |  | |
| 2010-2012 | -0.74 | -7.09 | -1.30 | -6.68 | -2.11 | -6.47 | -9.05 | -1.39 | -10.33 | -8.60 |  | |
| 2011-2013 | -0.62 | -7.01 | -2.49 | -4.14 | -2.98 | -6.42 | -9.77 | -1.62 | -8.77 | -7.21 |  | |
| 2012-2014 | 0.00 | -5.43 | -0.84 | -1.35 | -3.39 | -4.33 | -8.49 | -0.71 | -7.34 | -5.32 |  | |
| 2013-2015 | -0.04 | -5.88 | -0.56 | -2.10 | -3.57 | -4.36 | -6.73 | -0.65 | -6.35 | -6.42 |  | |
| 2014-2016 | -0.33 | -4.82 | -0.31 | -2.22 | -4.25 | -3.51 | -3.94 | -0.69 | -5.96 | -9.97 |  | |
