# Supplementary material for: Chesapeake Bay Dissolved Oxygen Criterion Attainment Deficit: Three Decades of Temporal and Spatial Patterns
Source: Front Mar Sci. Author manuscript; Available in PMC 2019 Sep 18. (PMC6750769; doi:10.3389/fmars.2018.00422)
Supplement: Supplementary C [file NIHMS1042326-supplement-Supplementary_C.pdf]

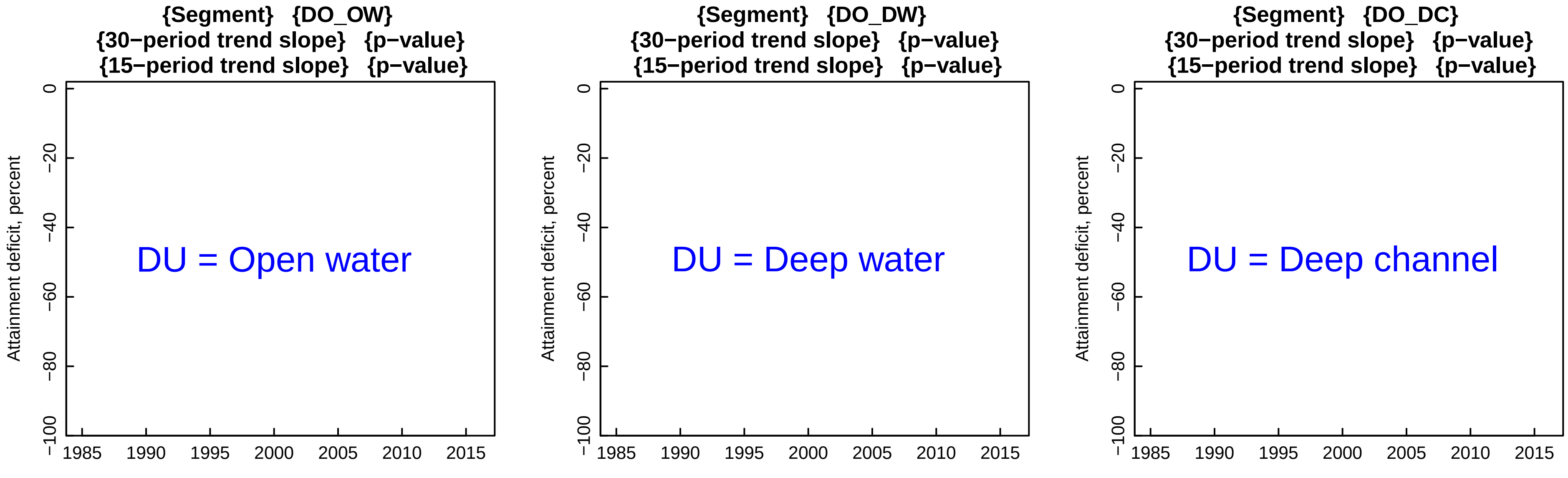
{Segment} {DO_OW}
{30−period trend slope} {p−value}
{15−period trend slope} {p−value}
DU = Open water
Attainment deficit, percent
0
−20
−40
−60
−80
−100
1985
1990
1995
2000
2005
2010
2015
{Segment} {DO_DW}
{30−period trend slope} {p−value}
{15−period trend slope} {p−value}
DU = Deep water
Attainment deficit, percent
{Segment} {DO_DC}
{30−period trend slope} {p−value}
{15−period trend slope} {p−value}
DU = Deep channel
Attainment deficit, percent

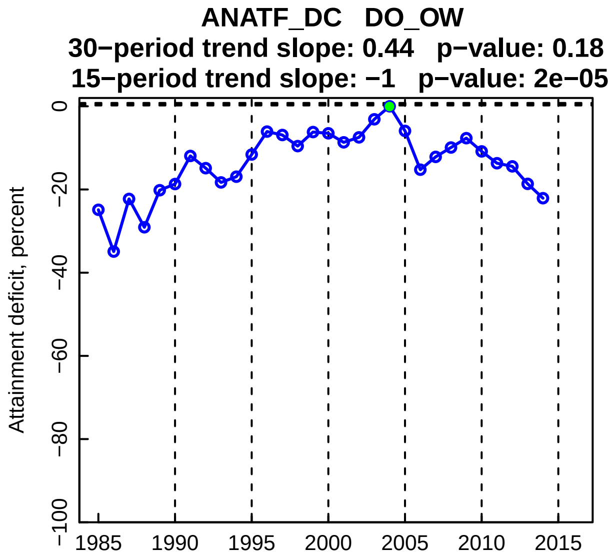
ANATF_DC DO_OW
30−period trend slope: 0.44 p−value: 0.18
15−period trend slope: −1 p−value: 2e−05
Attainment deficit, percent
0
−20
−40
−60
−80
−100
1985
1990
1995
2000
2005
2010
2015

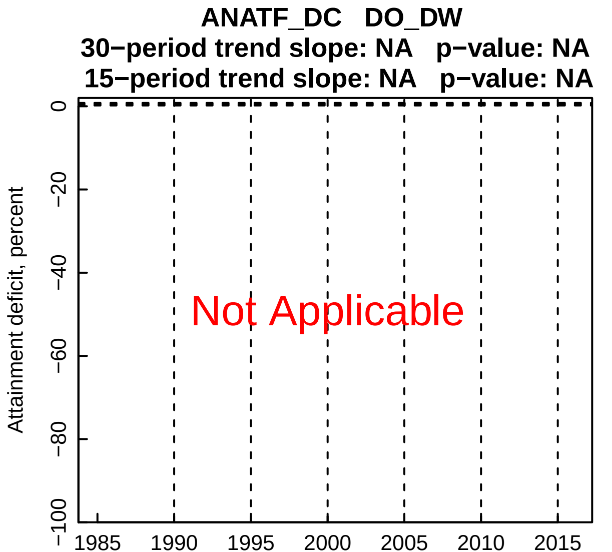
ANATF_DC DO_DW
30−period trend slope: NA p−value: NA
15−period trend slope: NA p−value: NA
Attainment deficit, percent
Not Applicable
0
−20
−40
−60
−80
−100
1985
1990
1995
2000
2005
2010
2015

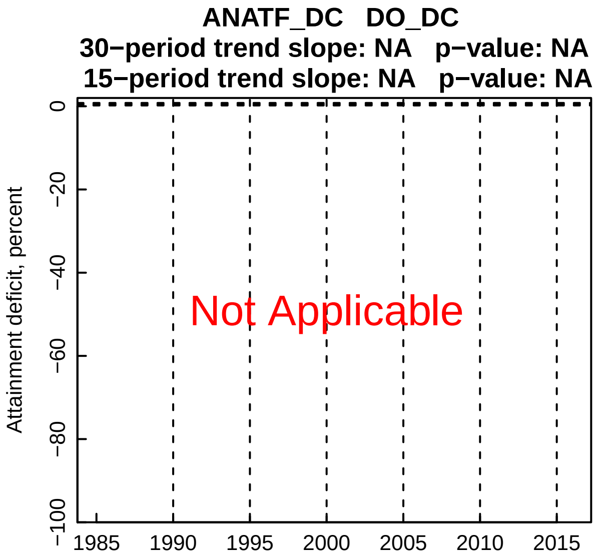
ANATF_DC DO_DC
30−period trend slope: NA p−value: NA
15−period trend slope: NA p−value: NA
Attainment deficit, percent
Not Applicable
0
−20
−40
−60
−80
−100
1985
1990
1995
2000
2005
2010
2015

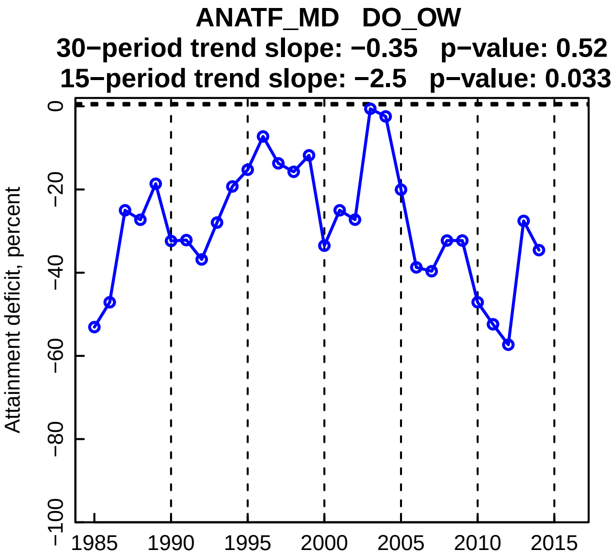

ANATF_MD DO_OW
30−period trend slope: −0.35 p−value: 0.52
15−period trend slope: −2.5 p−value: 0.033
Attainment deficit, percent
0
−20
−40
−60
−80
−100
1985
1990
1995
2000
2005
2010
2015

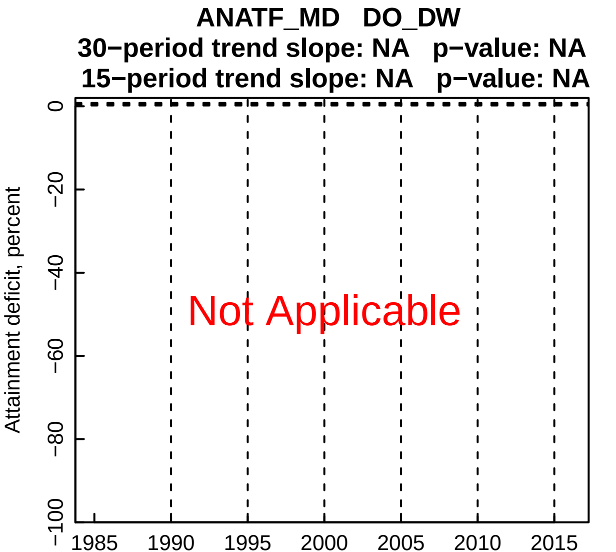

ANATF_MD DO_DW
30−period trend slope: NA p−value: NA
15−period trend slope: NA p−value: NA
Attainment deficit, percent
Not Applicable
0
−20
−40
−60
−80
−100
1985
1990
1995
2000
2005
2010
2015

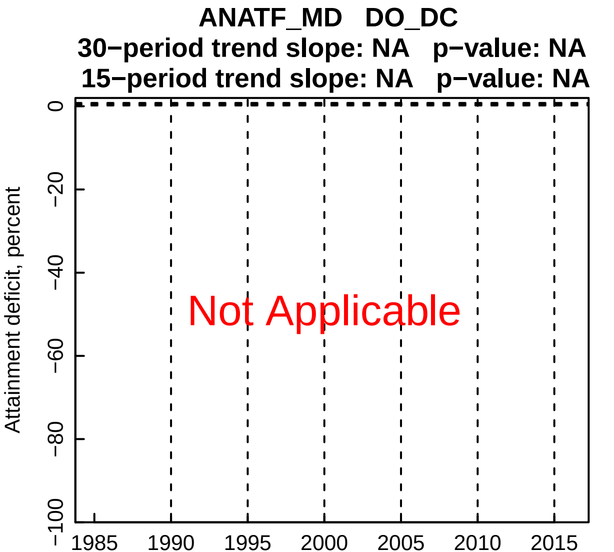

ANATF_MD DO_DC
30−period trend slope: NA p−value: NA
15−period trend slope: NA p−value: NA
Attainment deficit, percent
Not Applicable
0
−20
−40
−60
−80
−100
1985
1990
1995
2000
2005
2010
2015

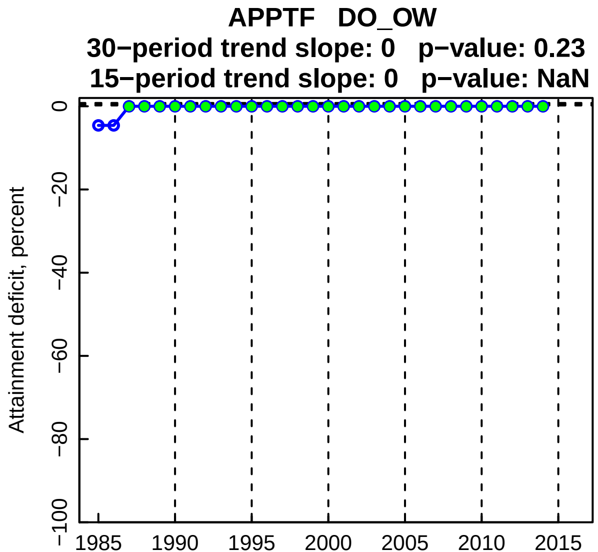
APPTF DO_OW
30−period trend slope: 0 p−value: 0.23
15−period trend slope: 0 p−value: NaN
Attainment deficit, percent
0
−20
−40
−60
−80
−100
1985
1990
1995
2000
2005
2010
2015

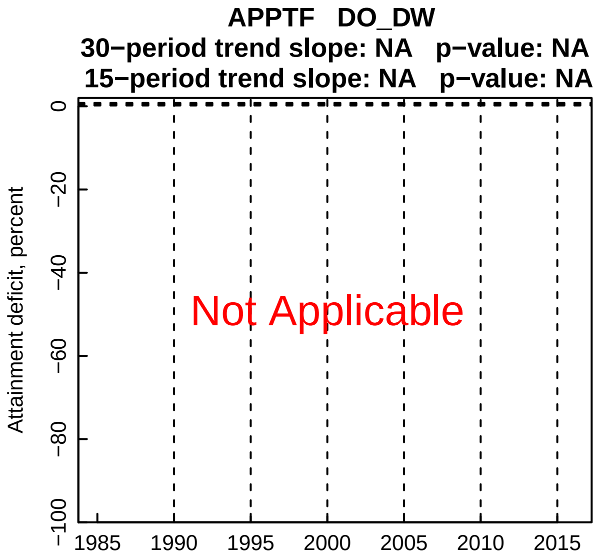
APPTF DO_DW
30−period trend slope: NA p−value: NA
15−period trend slope: NA p−value: NA
Attainment deficit, percent
Not Applicable
0
−20
−40
−60
−80
−100
1985
1990
1995
2000
2005
2010
2015

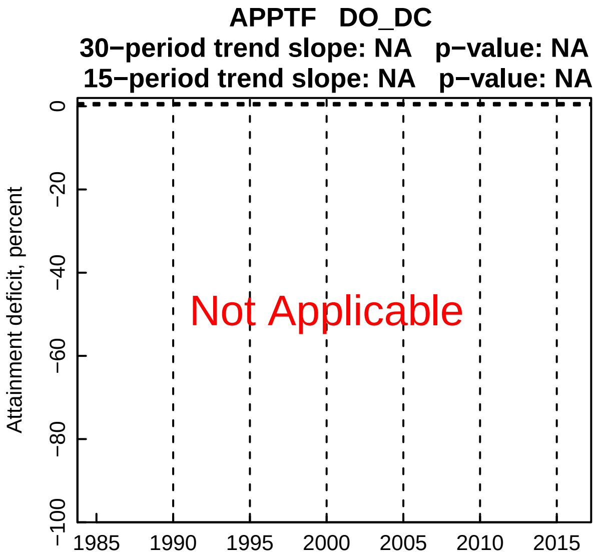
APPTF DO_DC
30−period trend slope: NA p−value: NA
15−period trend slope: NA p−value: NA
Attainment deficit, percent
Not Applicable
0
−20
−40
−60
−80
−100
1985
1990
1995
2000
2005
2010
2015

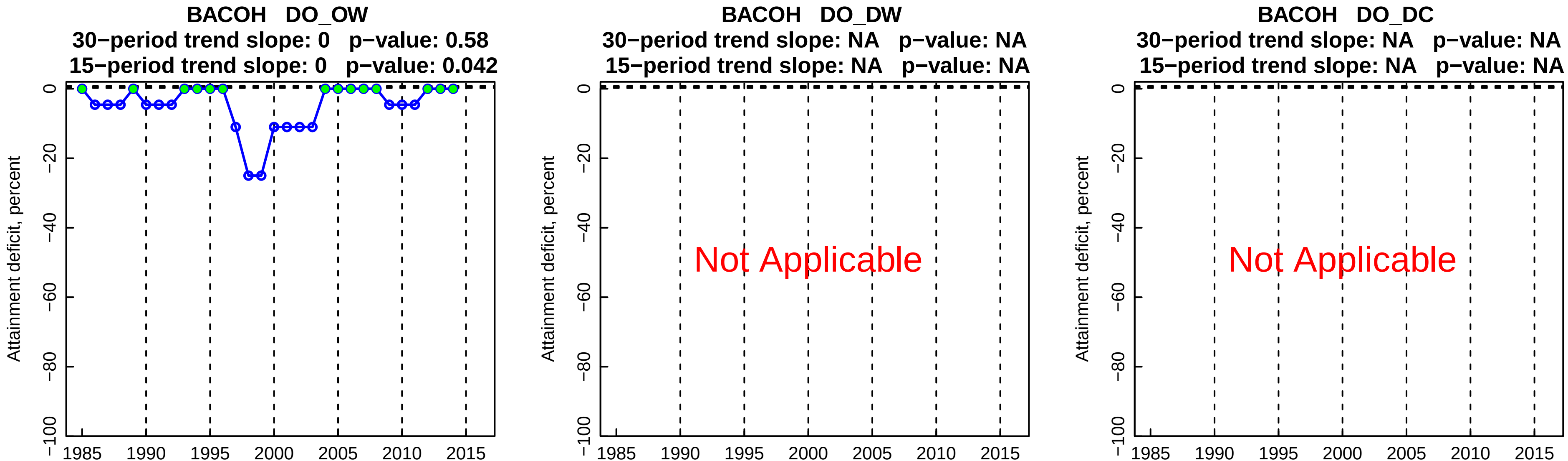

BACOH DO_OW
30−period trend slope: 0 p−value: 0.58
15−period trend slope: 0 p−value: 0.042
Attainment deficit, percent
0
−20
−40
−60
−80
−100
1985 1990 1995 2000 2005 2010 2015
BACOH DO_DW
30−period trend slope: NA p−value: NA
15−period trend slope: NA p−value: NA
Not Applicable
BACOH DO_DC
30−period trend slope: NA p−value: NA
15−period trend slope: NA p−value: NA
Not Applicable

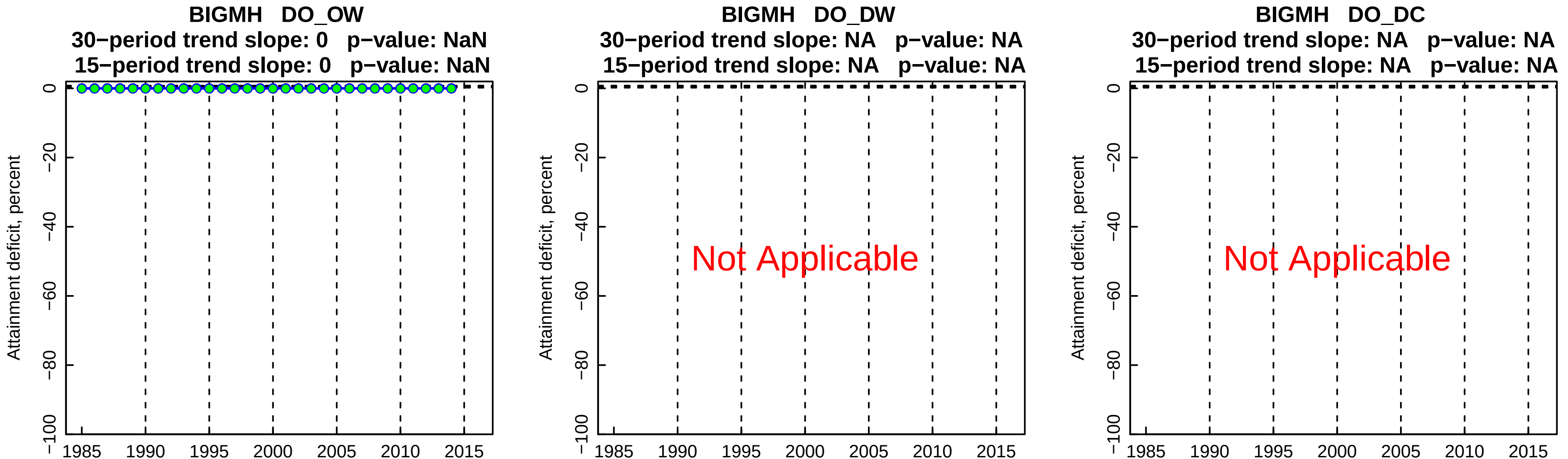

BIGMH DO_OW
30−period trend slope: 0 p−value: NaN
15−period trend slope: 0 p−value: NaN
Attainment deficit, percent
0
−20
−40
−60
−80
−100
1985
1990
1995
2000
2005
2010
2015
BIGMH DO_DW
30−period trend slope: NA p−value: NA
15−period trend slope: NA p−value: NA
Not Applicable
BIGMH DO_DC
30−period trend slope: NA p−value: NA
15−period trend slope: NA p−value: NA
Not Applicable

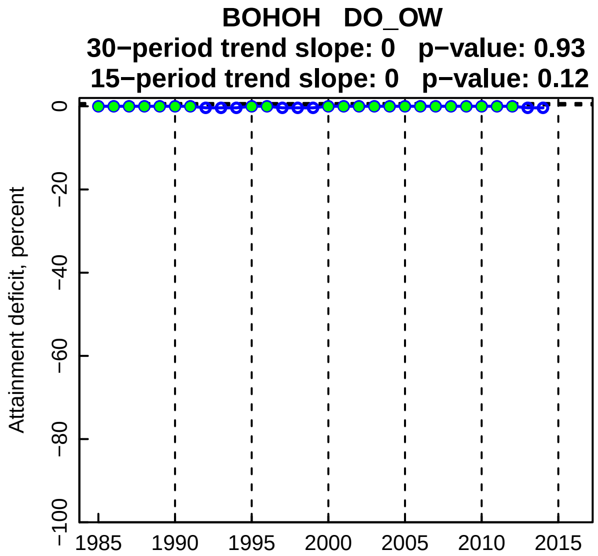
BOHOH DO_OW
30−period trend slope: 0 p−value: 0.93
15−period trend slope: 0 p−value: 0.12
Attainment deficit, percent
0
−20
−40
−60
−80
−100
1985
1990
1995
2000
2005
2010
2015

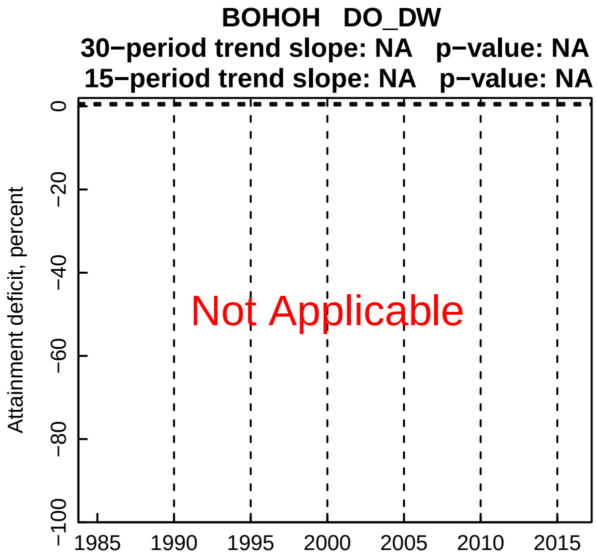
BOHOH DO_DW
30−period trend slope: NA p−value: NA
15−period trend slope: NA p−value: NA
Attainment deficit, percent
Not Applicable
0
−20
−40
−60
−80
−100
1985
1990
1995
2000
2005
2010
2015

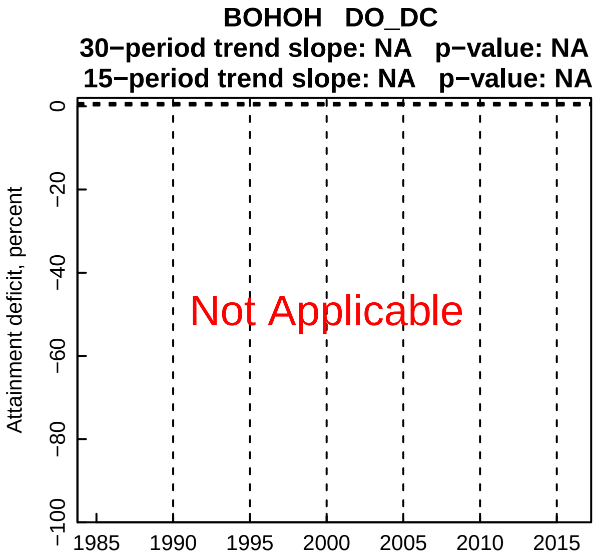
BOHOH DO_DC
30−period trend slope: NA p−value: NA
15−period trend slope: NA p−value: NA
Attainment deficit, percent
Not Applicable
0
−20
−40
−60
−80
−100
1985
1990
1995
2000
2005
2010
2015

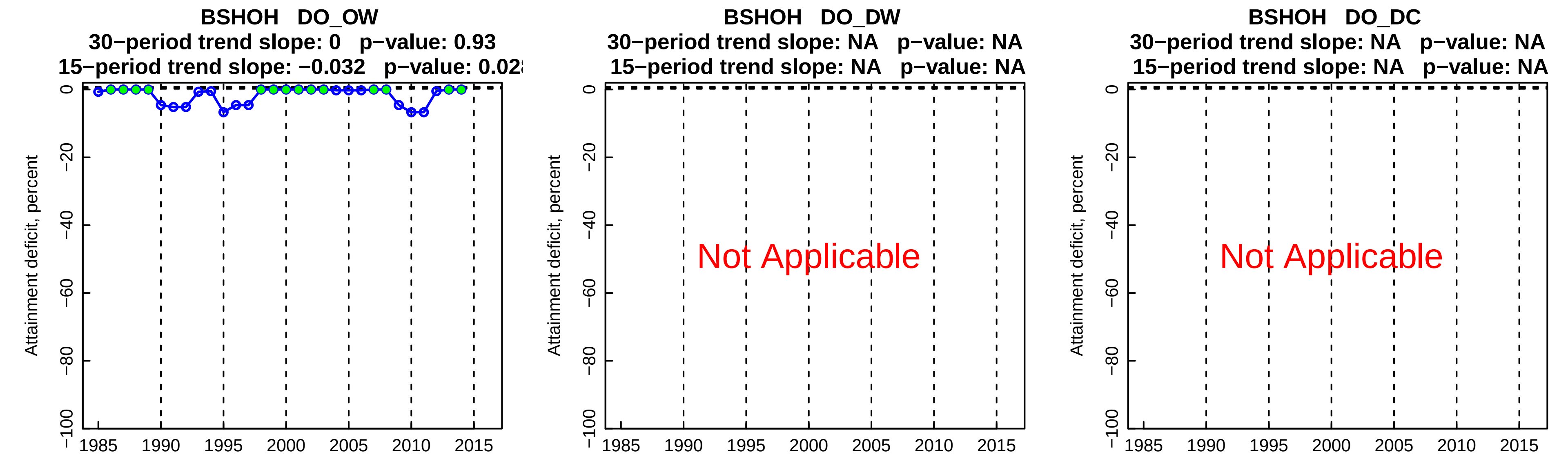
BSHOH DO_OW
30−period trend slope: 0 p−value: 0.93
15−period trend slope: −0.032 p−value: 0.02
Attainment deficit, percent
BSHOH DO_DW
30−period trend slope: NA p−value: NA
15−period trend slope: NA p−value: NA
Not Applicable
BSHOH DO_DC
30−period trend slope: NA p−value: NA
15−period trend slope: NA p−value: NA
Not Applicable
0
−20
−40
−60
−80
−100
1985
1990
1995
2000
2005
2010
2015

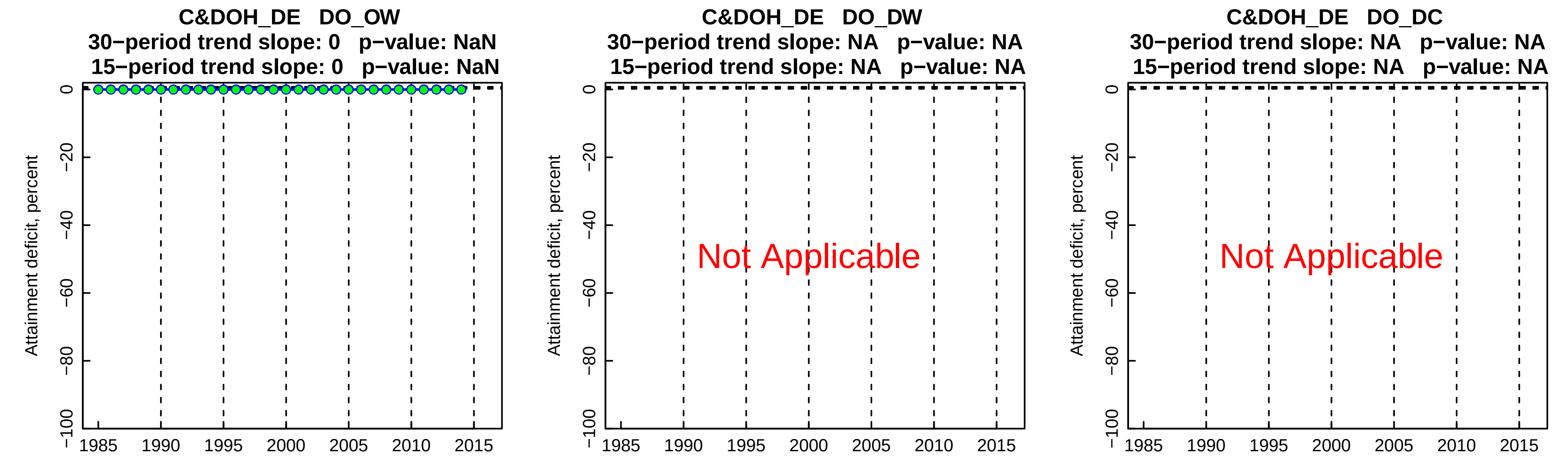
C&DOH_DE DO_OW
30−period trend slope: 0 p−value: NaN
15−period trend slope: 0 p−value: NaN
Attainment deficit, percent
0
−20
−40
−60
−80
−100
1985
1990
1995
2000
2005
2010
2015
C&DOH_DE DO_DW
30−period trend slope: NA p−value: NA
15−period trend slope: NA p−value: NA
Not Applicable
C&DOH_DE DO_DC
30−period trend slope: NA p−value: NA
15−period trend slope: NA p−value: NA
Not Applicable

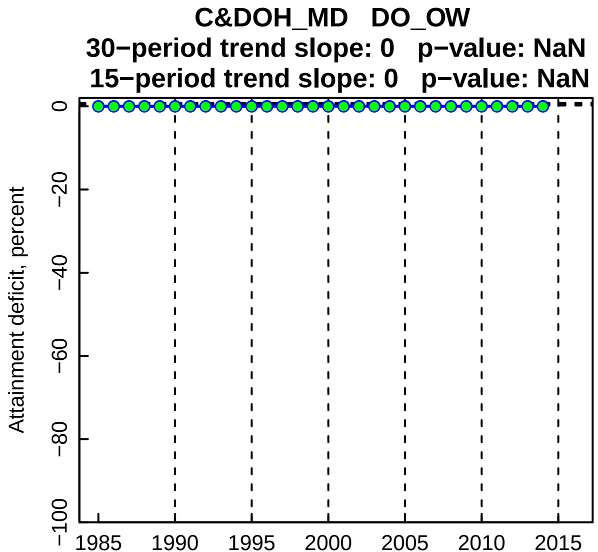
C&DOH_MD DO_OW
30−period trend slope: 0 p−value: NaN
15−period trend slope: 0 p−value: NaN
Attainment deficit, percent
0
−20
−40
−60
−80
−100
1985
1990
1995
2000
2005
2010
2015

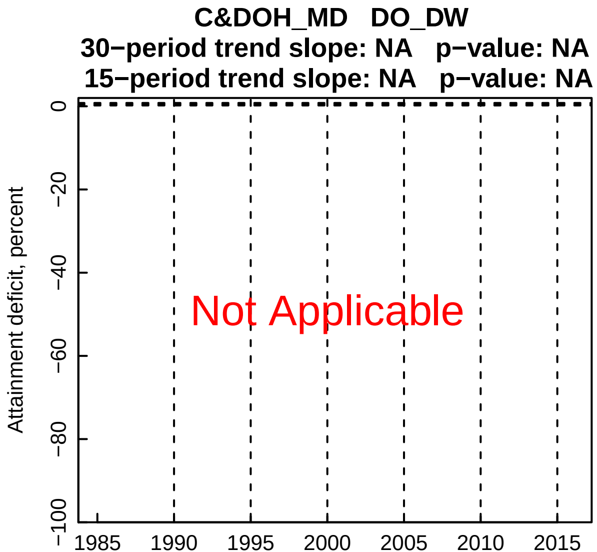
C&DOH_MD DO_DW
30−period trend slope: NA p−value: NA
15−period trend slope: NA p−value: NA
Attainment deficit, percent
Not Applicable
0
−20
−40
−60
−80
−100
1985
1990
1995
2000
2005
2010
2015

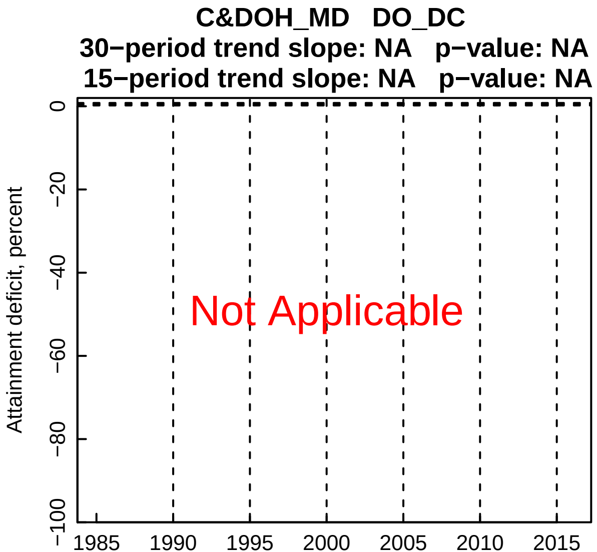
C&DOH_MD DO_DC
30−period trend slope: NA p−value: NA
15−period trend slope: NA p−value: NA
Attainment deficit, percent
Not Applicable
0
−20
−40
−60
−80
−100
1985
1990
1995
2000
2005
2010
2015

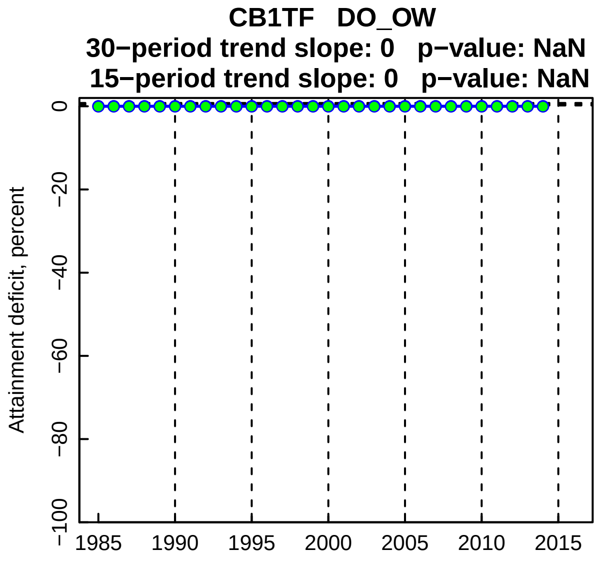
CB1TF DO_OW
30−period trend slope: 0 p−value: NaN
15−period trend slope: 0 p−value: NaN
Attainment deficit, percent
0
−20
−40
−60
−80
−100
1985
1990
1995
2000
2005
2010
2015

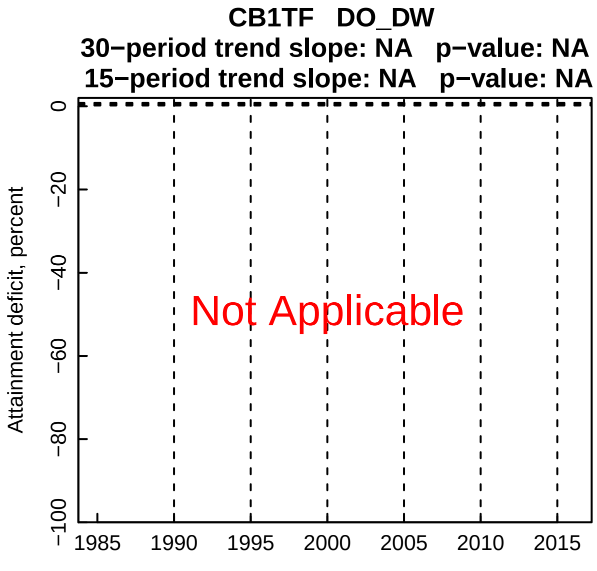
CB1TF DO_DW
30−period trend slope: NA p−value: NA
15−period trend slope: NA p−value: NA
Attainment deficit, percent
Not Applicable
0
−20
−40
−60
−80
−100
1985
1990
1995
2000
2005
2010
2015

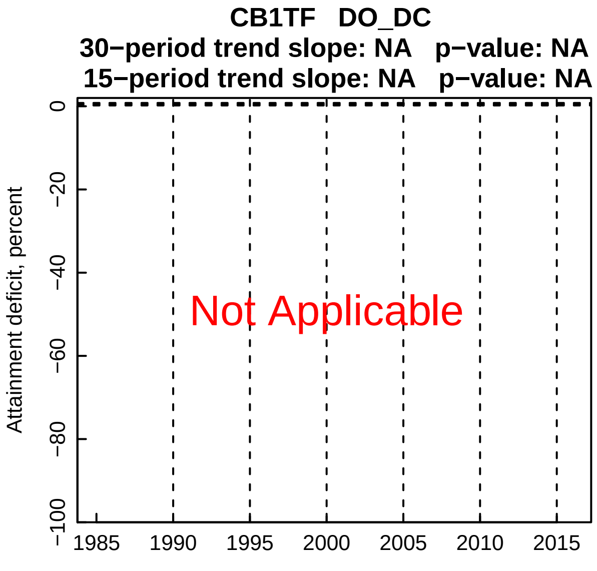
CB1TF DO_DC
30−period trend slope: NA p−value: NA
15−period trend slope: NA p−value: NA
Attainment deficit, percent
Not Applicable
0
−20
−40
−60
−80
−100
1985
1990
1995
2000
2005
2010
2015

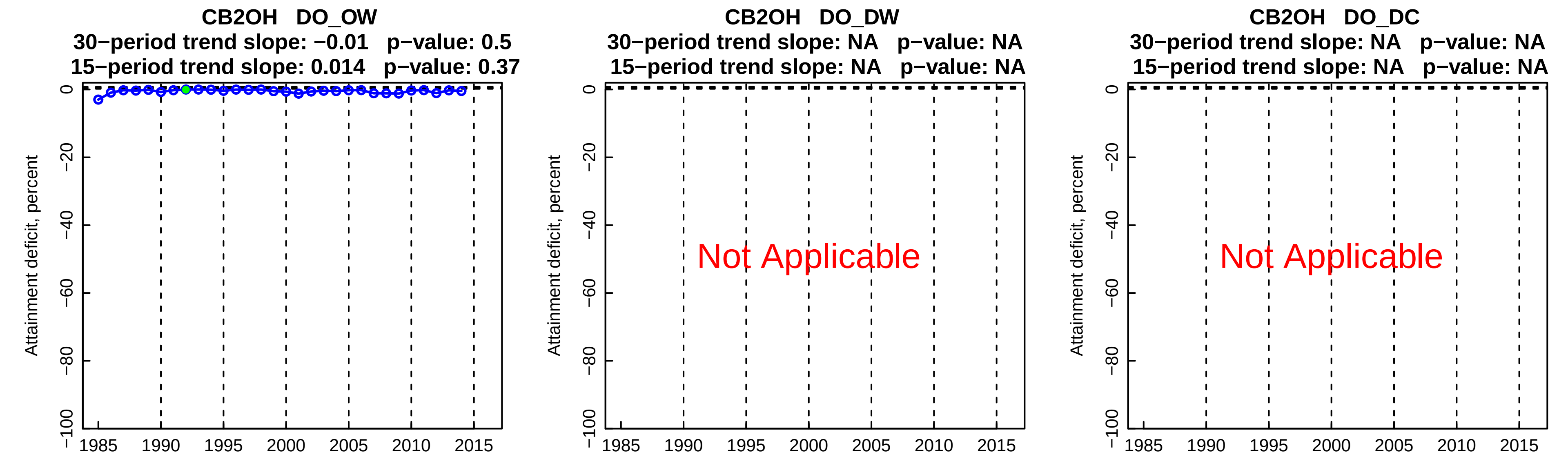
CB2OH DO_OW
30−period trend slope: −0.01 p−value: 0.5
15−period trend slope: 0.014 p−value: 0.37
Attainment deficit, percent
0
−20
−40
−60
−80
−100
1985
1990
1995
2000
2005
2010
2015
CB2OH DO_DW
30−period trend slope: NA p−value: NA
15−period trend slope: NA p−value: NA
Attainment deficit, percent
Not Applicable
CB2OH DO_DC
30−period trend slope: NA p−value: NA
15−period trend slope: NA p−value: NA
Attainment deficit, percent
Not Applicable

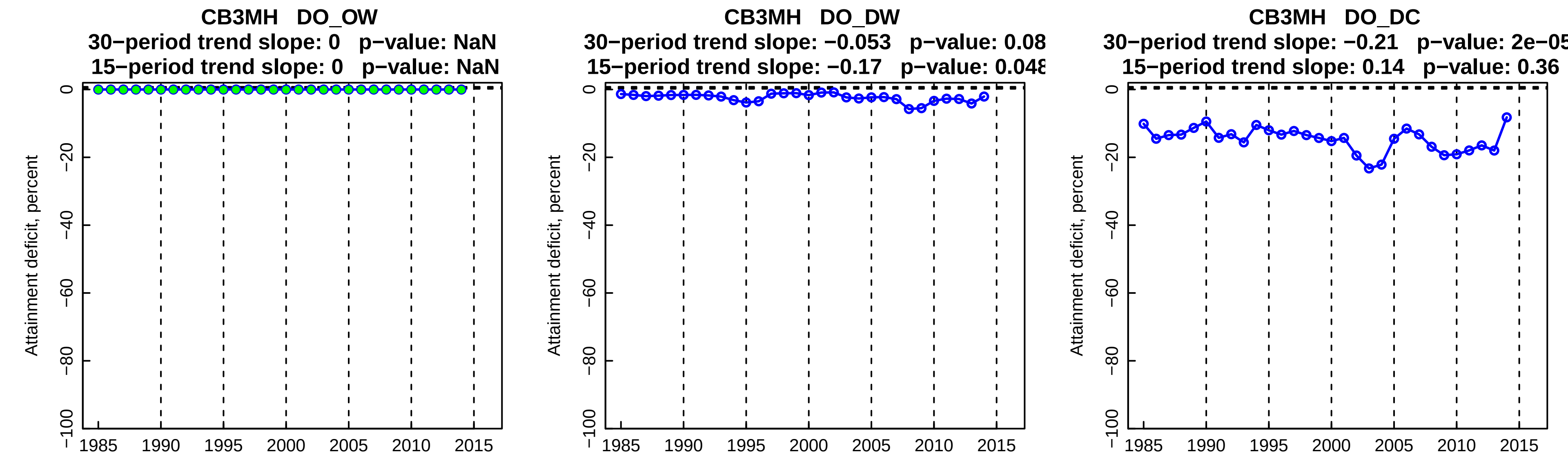
CB3MH DO_OW
30−period trend slope: 0 p−value: NaN
15−period trend slope: 0 p−value: NaN
CB3MH DO_DW
30−period trend slope: −0.053 p−value: 0.08
15−period trend slope: −0.17 p−value: 0.048
CB3MH DO_DC
30−period trend slope: −0.21 p−value: 2e−05
15−period trend slope: 0.14 p−value: 0.36
Attainment deficit, percent
0
−20
−40
−60
−80
−100
1985
1990
1995
2000
2005
2010
2015

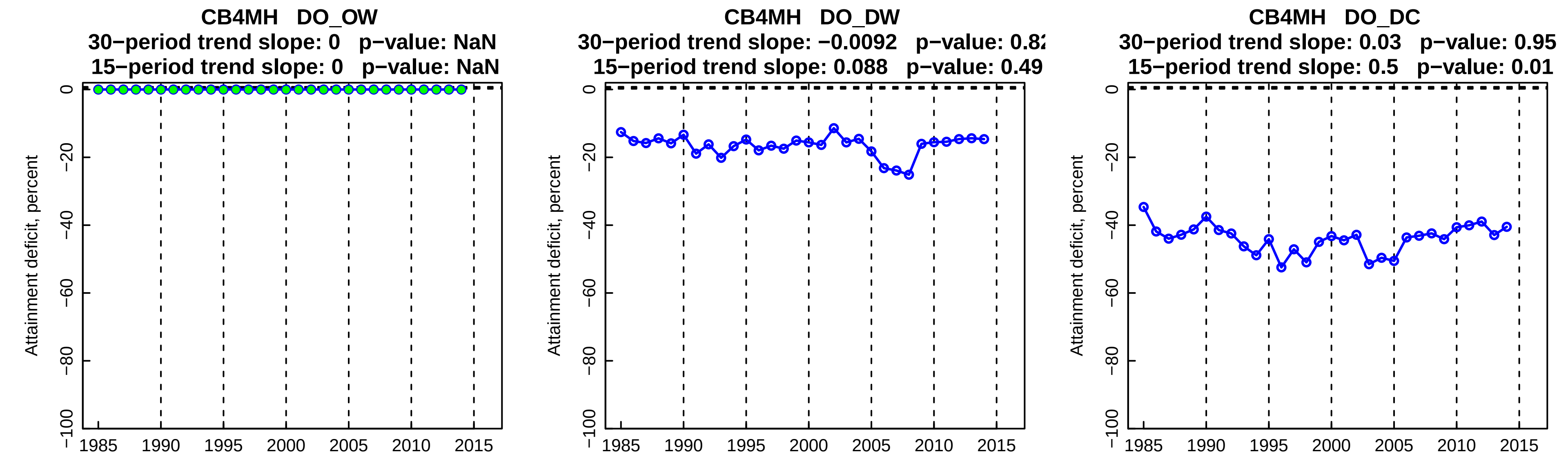
CB4MH DO_OW
30−period trend slope: 0 p−value: NaN
15−period trend slope: 0 p−value: NaN
CB4MH DO_DW
30−period trend slope: −0.0092 p−value: 0.82
15−period trend slope: 0.088 p−value: 0.49
CB4MH DO_DC
30−period trend slope: 0.03 p−value: 0.95
15−period trend slope: 0.5 p−value: 0.01
Attainment deficit, percent
0
−20
−40
−60
−80
−100
1985
1990
1995
2000
2005
2010
2015

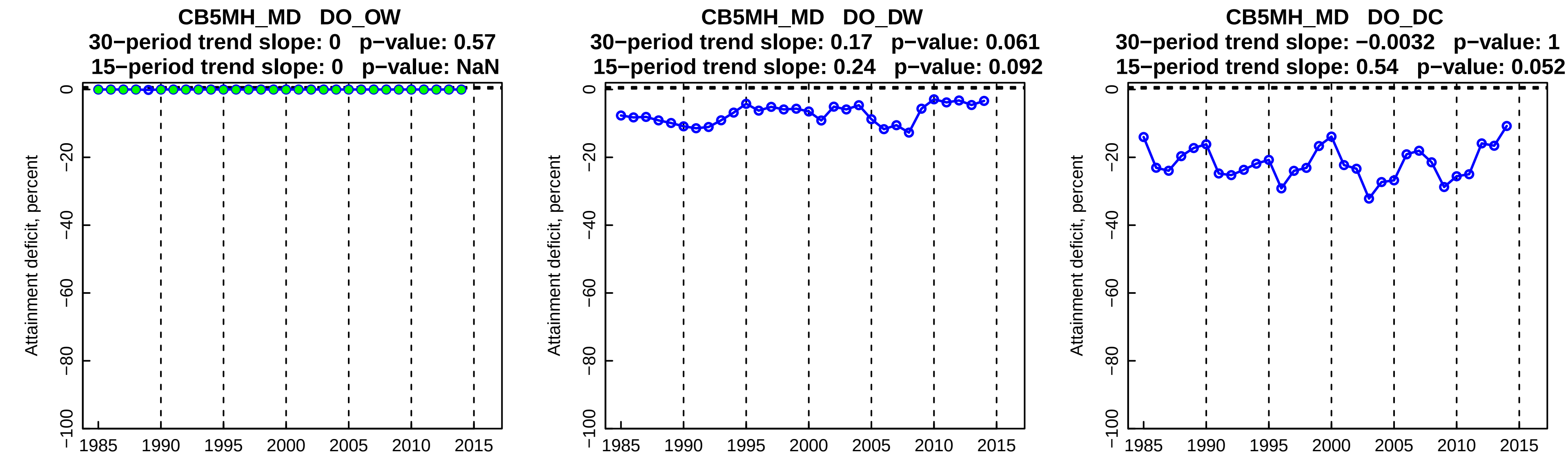

CB5MH_MD DO_OW
30−period trend slope: 0 p−value: 0.57
15−period trend slope: 0 p−value: NaN
Attainment deficit, percent
0
−20
−40
−60
−80
−100
1985
1990
1995
2000
2005
2010
2015
CB5MH_MD DO_DW
30−period trend slope: 0.17 p−value: 0.061
15−period trend slope: 0.24 p−value: 0.092
Attainment deficit, percent
CB5MH_MD DO_DC
30−period trend slope: −0.0032 p−value: 1
15−period trend slope: 0.54 p−value: 0.052
Attainment deficit, percent

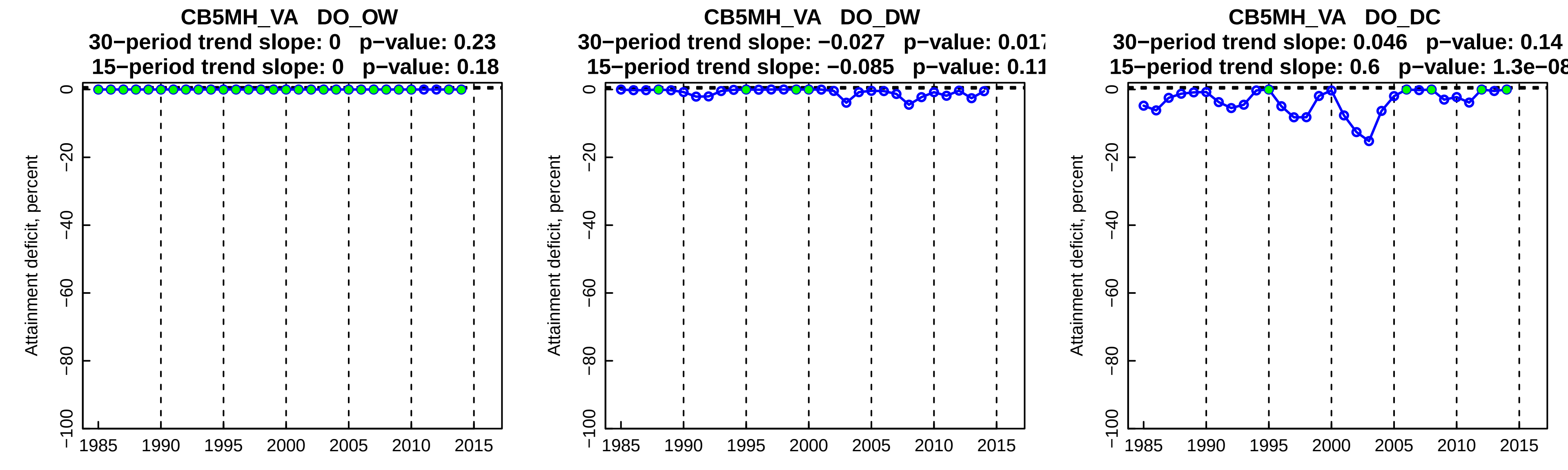
CB5MH_VA DO_OW
30−period trend slope: 0 p−value: 0.23
15−period trend slope: 0 p−value: 0.18
Attainment deficit, percent
CB5MH_VA DO_DW
30−period trend slope: −0.027 p−value: 0.017
15−period trend slope: −0.085 p−value: 0.11
Attainment deficit, percent
CB5MH_VA DO_DC
30−period trend slope: 0.046 p−value: 0.14
15−period trend slope: 0.6 p−value: 1.3e−08
Attainment deficit, percent
0
−20
−40
−60
−80
−100
1985 1990 1995 2000 2005 2010 2015

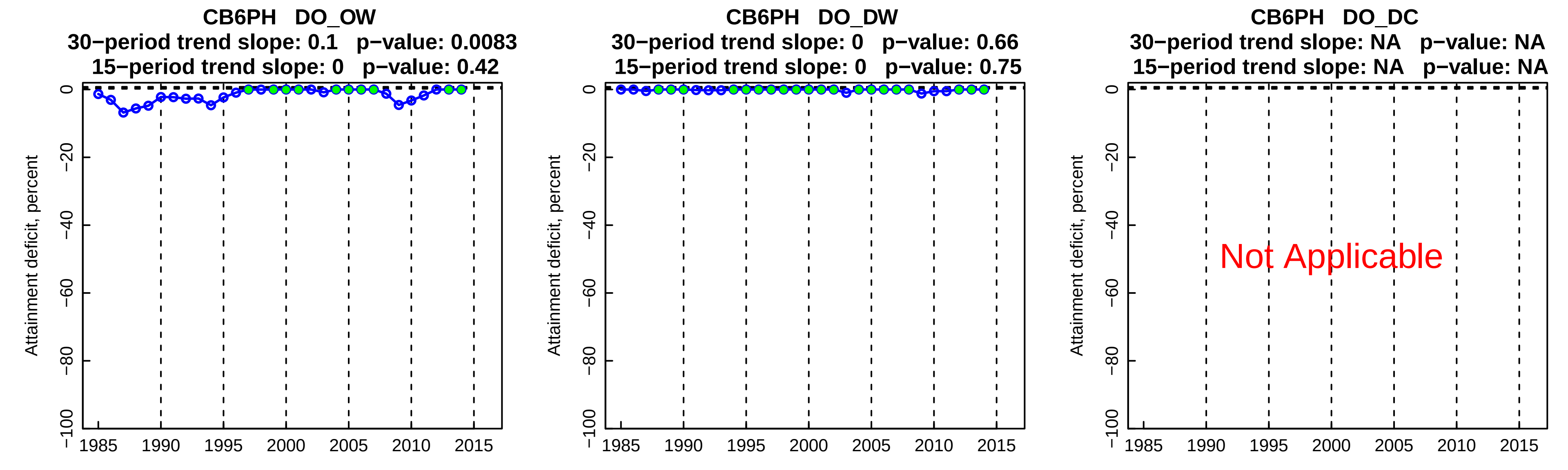
CB6PH DO_OW
30−period trend slope: 0.1 p−value: 0.0083
15−period trend slope: 0 p−value: 0.42
Attainment deficit, percent
0
−20
−40
−60
−80
−100
1985
1990
1995
2000
2005
2010
2015
CB6PH DO_DW
30−period trend slope: 0 p−value: 0.66
15−period trend slope: 0 p−value: 0.75
Attainment deficit, percent
CB6PH DO_DC
30−period trend slope: NA p−value: NA
15−period trend slope: NA p−value: NA
Attainment deficit, percent
Not Applicable

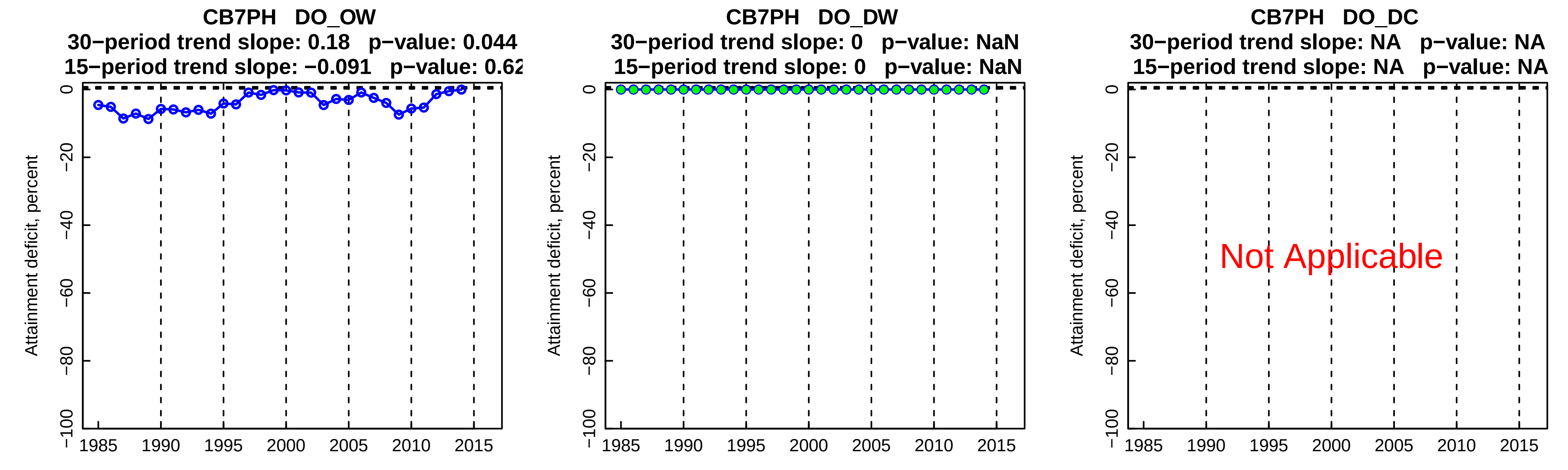

CB7PH DO_OW
30−period trend slope: 0.18 p−value: 0.044
15−period trend slope: −0.091 p−value: 0.62
Attainment deficit, percent
0
−20
−40
−60
−80
−100
1985
1990
1995
2000
2005
2010
2015
CB7PH DO_DW
30−period trend slope: 0 p−value: NaN
15−period trend slope: 0 p−value: NaN
Attainment deficit, percent
CB7PH DO_DC
30−period trend slope: NA p−value: NA
15−period trend slope: NA p−value: NA
Attainment deficit, percent
Not Applicable

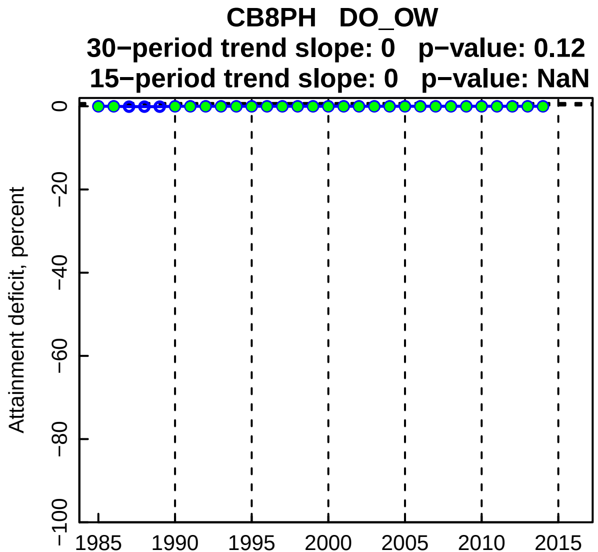
CB8PH DO_OW
30−period trend slope: 0 p−value: 0.12
15−period trend slope: 0 p−value: NaN
Attainment deficit, percent
0
−20
−40
−60
−80
−100
1985
1990
1995
2000
2005
2010
2015

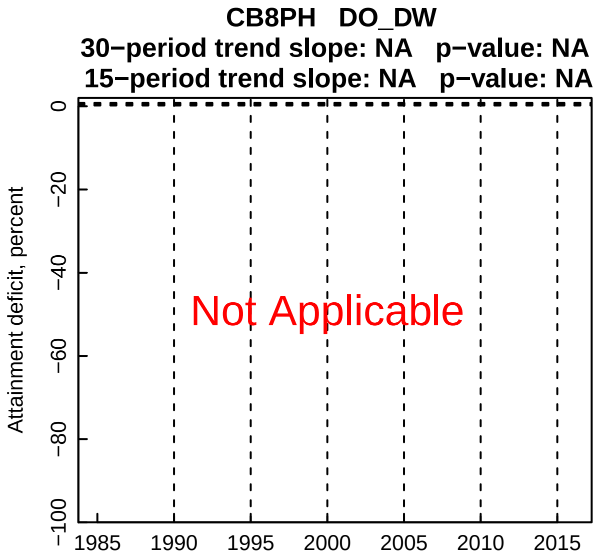
CB8PH DO_DW
30−period trend slope: NA p−value: NA
15−period trend slope: NA p−value: NA
Attainment deficit, percent
Not Applicable
0
−20
−40
−60
−80
−100
1985
1990
1995
2000
2005
2010
2015

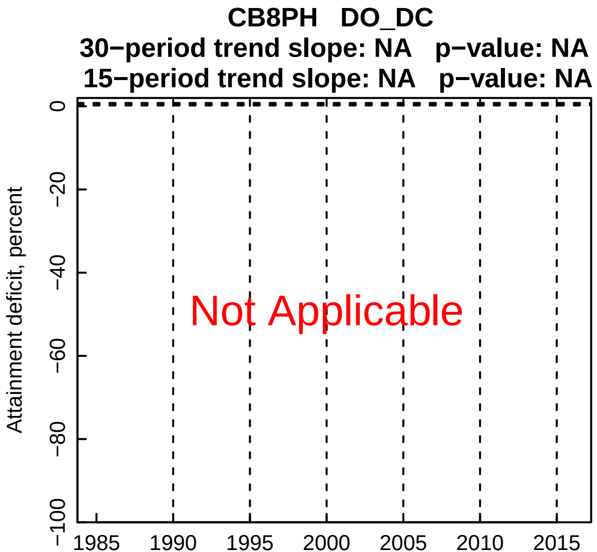
CB8PH DO_DC
30−period trend slope: NA p−value: NA
15−period trend slope: NA p−value: NA
Attainment deficit, percent
Not Applicable
0
−20
−40
−60
−80
−100
1985
1990
1995
2000
2005
2010
2015

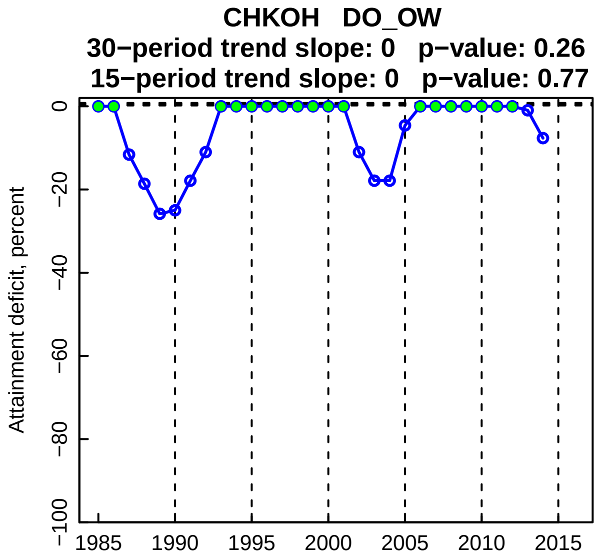
CHKOH DO_OW
30−period trend slope: 0 p−value: 0.26
15−period trend slope: 0 p−value: 0.77
Attainment deficit, percent
0
−20
−40
−60
−80
−100
1985
1990
1995
2000
2005
2010
2015

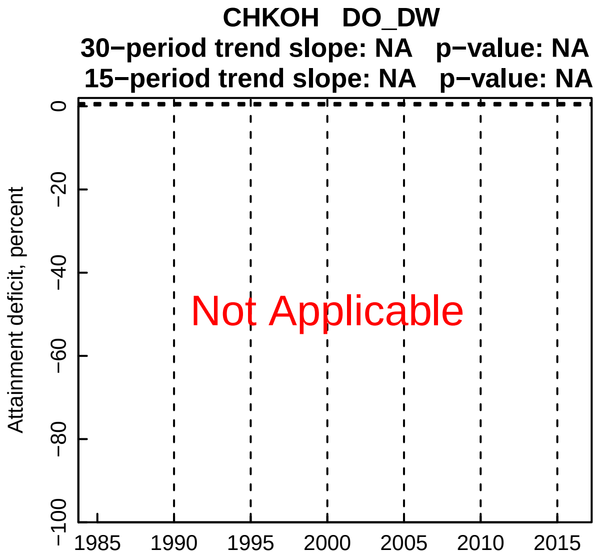
CHKOH DO_DW
30−period trend slope: NA p−value: NA
15−period trend slope: NA p−value: NA
Attainment deficit, percent
Not Applicable
0
−20
−40
−60
−80
−100
1985
1990
1995
2000
2005
2010
2015

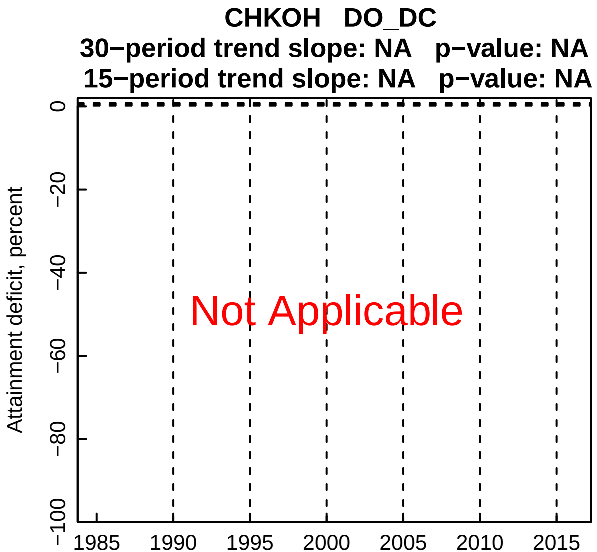
CHKOH DO_DC
30−period trend slope: NA p−value: NA
15−period trend slope: NA p−value: NA
Attainment deficit, percent
Not Applicable
0
−20
−40
−60
−80
−100
1985
1990
1995
2000
2005
2010
2015

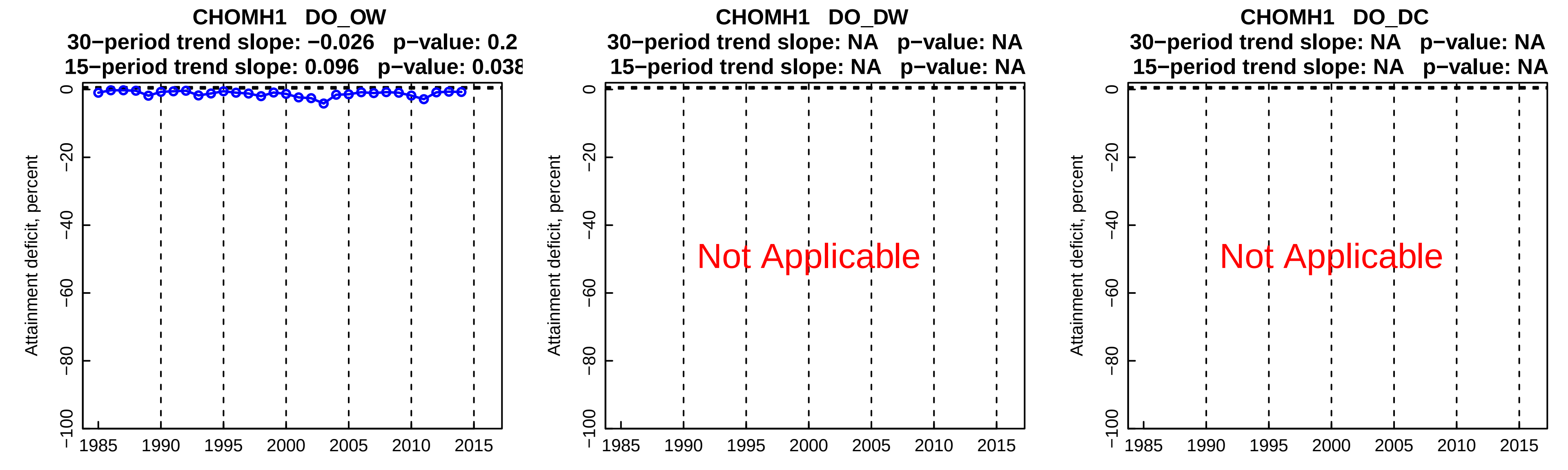
CHOMH1 DO_OW
30−period trend slope: −0.026 p−value: 0.2
15−period trend slope: 0.096 p−value: 0.038
Attainment deficit, percent
0
−20
−40
−60
−80
−100
1985
1990
1995
2000
2005
2010
2015
CHOMH1 DO_DW
30−period trend slope: NA p−value: NA
15−period trend slope: NA p−value: NA
Not Applicable
CHOMH1 DO_DC
30−period trend slope: NA p−value: NA
15−period trend slope: NA p−value: NA
Not Applicable

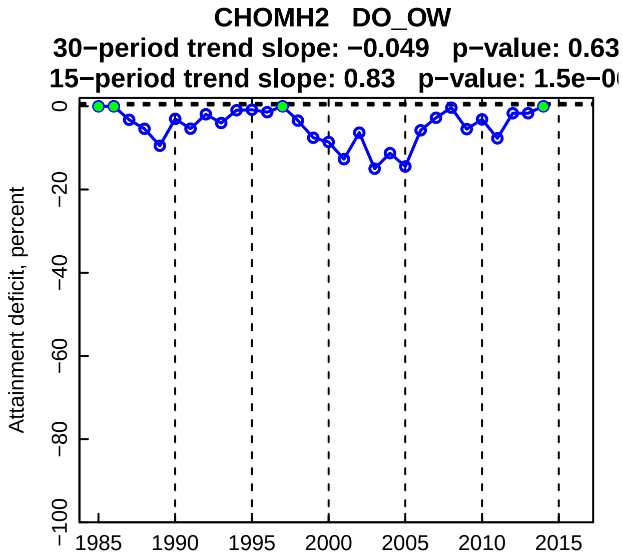

CHOMH2 DO_OW
30−period trend slope: −0.049 p−value: 0.63
15−period trend slope: 0.83 p−value: 1.5e−0
Attainment deficit, percent
0
−20
−40
−60
−80
−100
1985 1990 1995 2000 2005 2010 2015

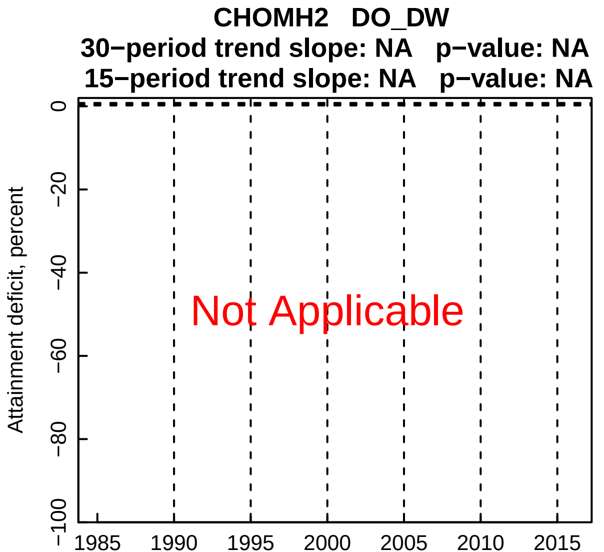

CHOMH2 DO_DW
30−period trend slope: NA p−value: NA
15−period trend slope: NA p−value: NA
Attainment deficit, percent
Not Applicable
0
−20
−40
−60
−80
−100
1985 1990 1995 2000 2005 2010 2015

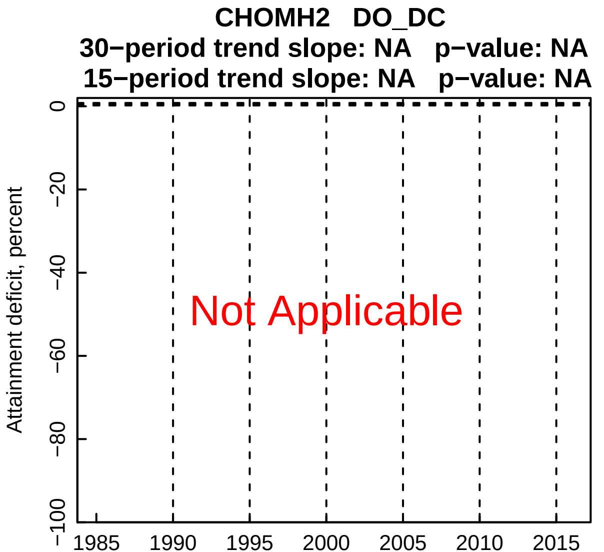

CHOMH2 DO_DC
30−period trend slope: NA p−value: NA
15−period trend slope: NA p−value: NA
Attainment deficit, percent
Not Applicable
0
−20
−40
−60
−80
−100
1985 1990 1995 2000 2005 2010 2015

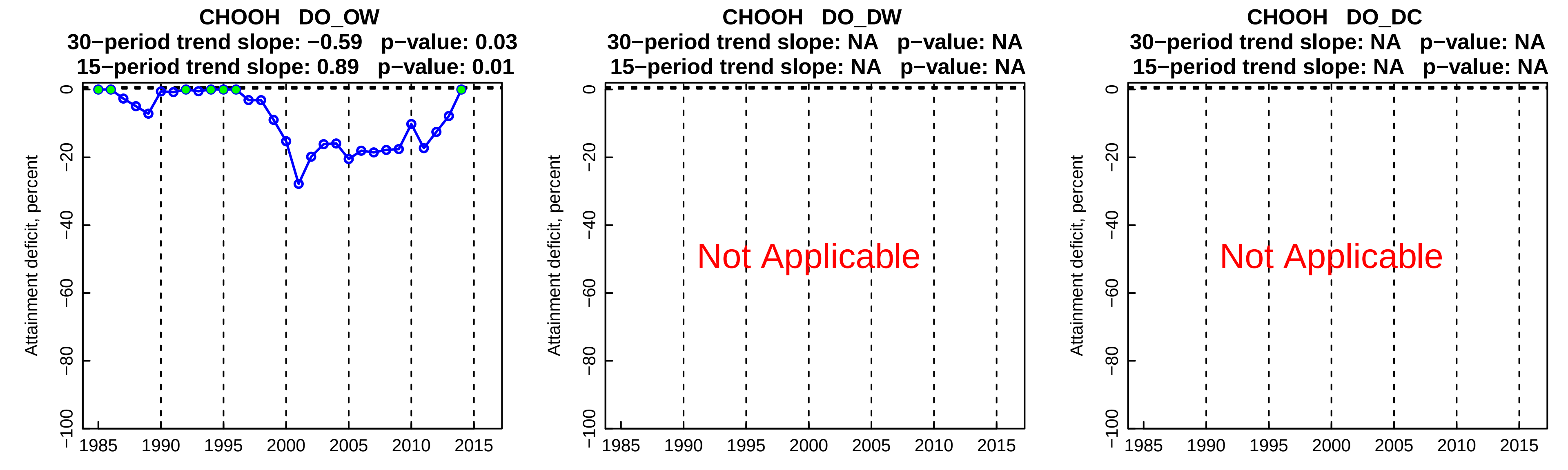
CHOOH DO_OW
30−period trend slope: −0.59 p−value: 0.03
15−period trend slope: 0.89 p−value: 0.01
Attainment deficit, percent
CHOOH DO_DW
30−period trend slope: NA p−value: NA
15−period trend slope: NA p−value: NA
Not Applicable
CHOOH DO_DC
30−period trend slope: NA p−value: NA
15−period trend slope: NA p−value: NA
Not Applicable

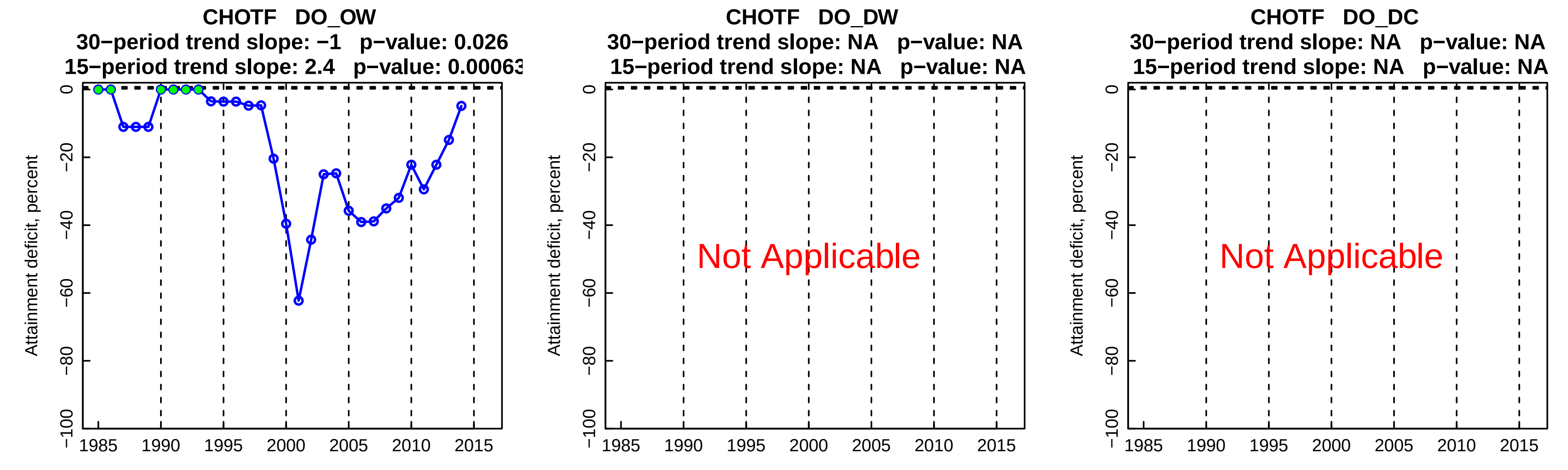
CHOTF DO_OW
30−period trend slope: −1 p−value: 0.026
15−period trend slope: 2.4 p−value: 0.00063
CHOTF DO_DW
30−period trend slope: NA p−value: NA
15−period trend slope: NA p−value: NA
Not Applicable
CHOTF DO_DC
30−period trend slope: NA p−value: NA
15−period trend slope: NA p−value: NA
Not Applicable
Attainment deficit, percent
0
−20
−40
−60
−80
−100
1985
1990
1995
2000
2005
2010
2015

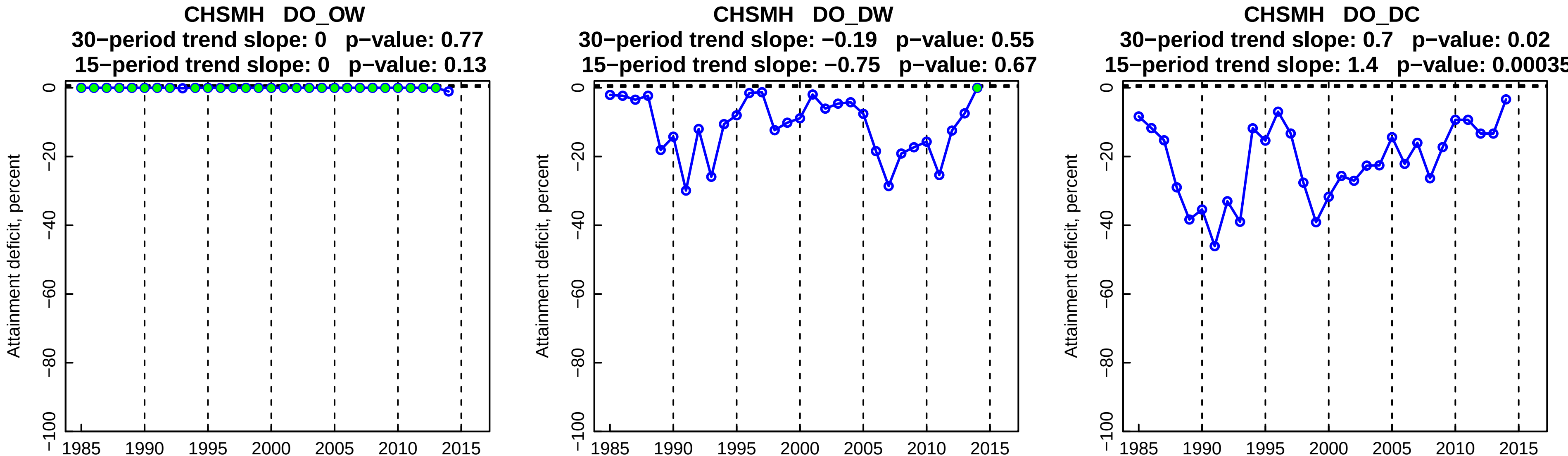
CHSMH DO_OW
30−period trend slope: 0 p−value: 0.77
15−period trend slope: 0 p−value: 0.13
CHSMH DO_DW
30−period trend slope: −0.19 p−value: 0.55
15−period trend slope: −0.75 p−value: 0.67
CHSMH DO_DC
30−period trend slope: 0.7 p−value: 0.02
15−period trend slope: 1.4 p−value: 0.00035
Attainment deficit, percent
0
−20
−40
−60
−80
−100
1985
1990
1995
2000
2005
2010
2015

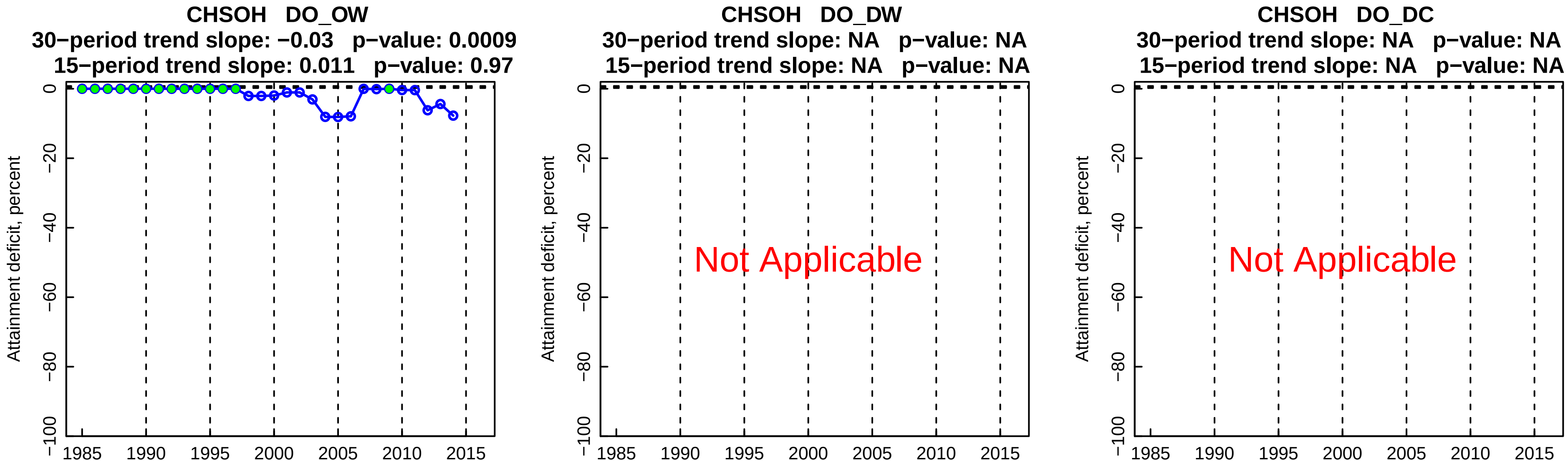
CHSOH DO_OW
30−period trend slope: −0.03 p−value: 0.0009
15−period trend slope: 0.011 p−value: 0.97
CHSOH DO_DW
30−period trend slope: NA p−value: NA
15−period trend slope: NA p−value: NA
Not Applicable
CHSOH DO_DC
30−period trend slope: NA p−value: NA
15−period trend slope: NA p−value: NA
Not Applicable
Attainment deficit, percent
0
−20
−40
−60
−80
−100
1985
1990
1995
2000
2005
2010
2015

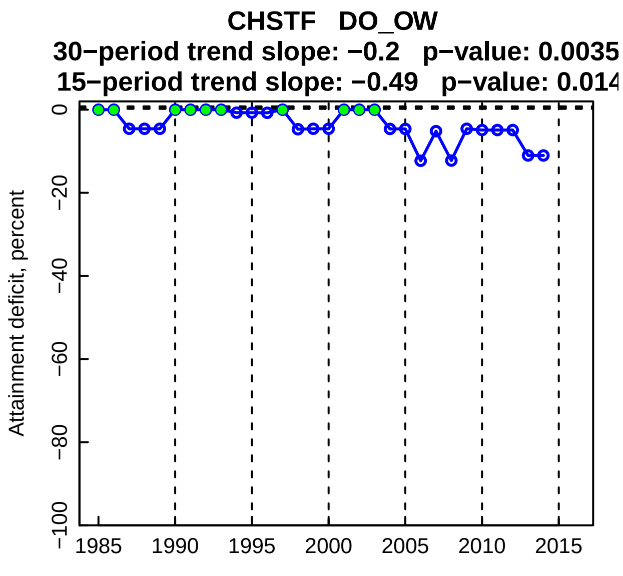

CHSTF DO_OW
30−period trend slope: −0.2 p−value: 0.0035
15−period trend slope: −0.49 p−value: 0.014
Attainment deficit, percent
0
−20
−40
−60
−80
−100
1985
1990
1995
2000
2005
2010
2015

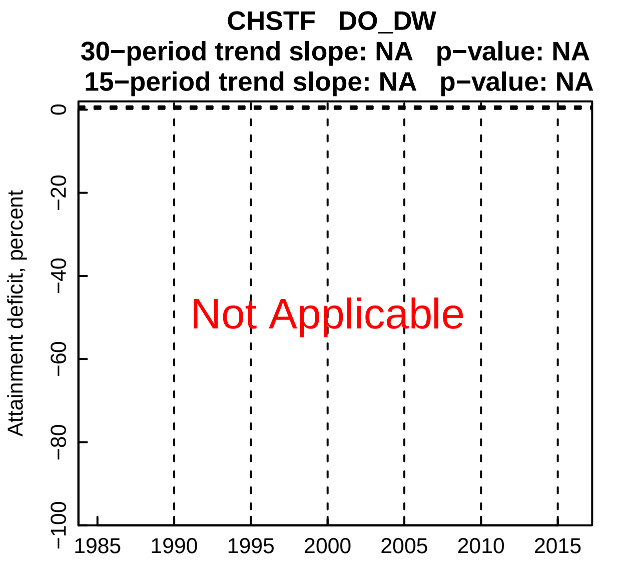

CHSTF DO_DW
30−period trend slope: NA p−value: NA
15−period trend slope: NA p−value: NA
Attainment deficit, percent
Not Applicable
0
−20
−40
−60
−80
−100
1985
1990
1995
2000
2005
2010
2015

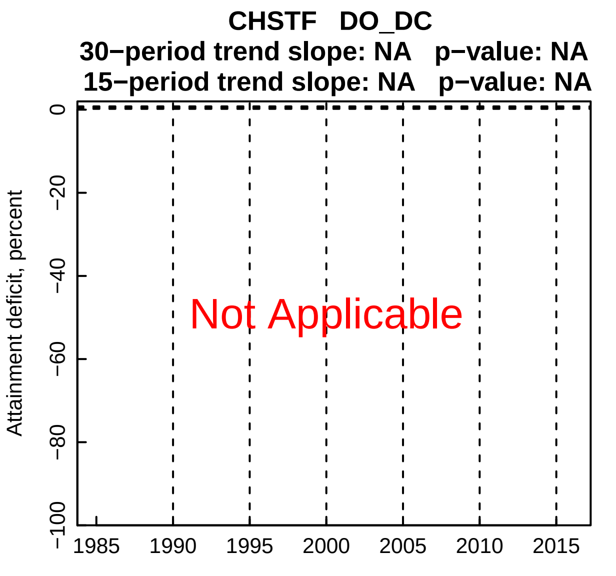

CHSTF DO_DC
30−period trend slope: NA p−value: NA
15−period trend slope: NA p−value: NA
Attainment deficit, percent
Not Applicable
0
−20
−40
−60
−80
−100
1985
1990
1995
2000
2005
2010
2015

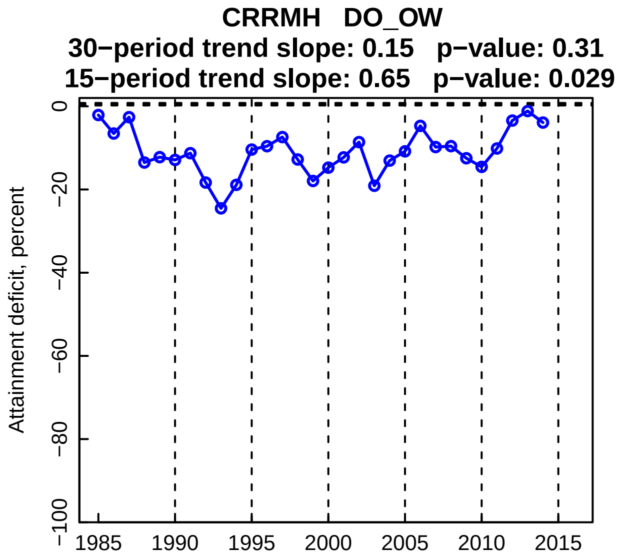
CRRMH DO_OW
30−period trend slope: 0.15 p−value: 0.31
15−period trend slope: 0.65 p−value: 0.029
Attainment deficit, percent
0
−20
−40
−60
−80
−100
1985
1990
1995
2000
2005
2010
2015

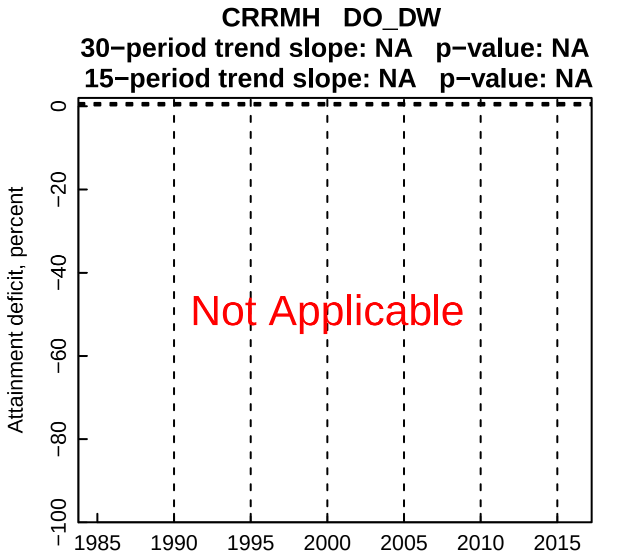
CRRMH DO_DW
30−period trend slope: NA p−value: NA
15−period trend slope: NA p−value: NA
Attainment deficit, percent
Not Applicable
0
−20
−40
−60
−80
−100
1985
1990
1995
2000
2005
2010
2015

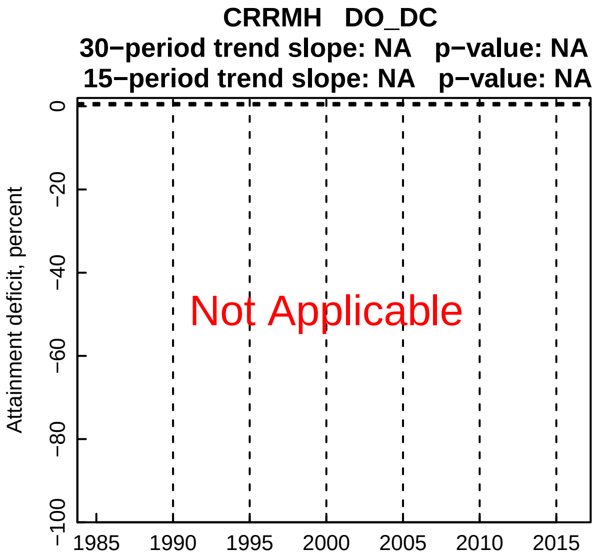
CRRMH DO_DC
30−period trend slope: NA p−value: NA
15−period trend slope: NA p−value: NA
Attainment deficit, percent
Not Applicable
0
−20
−40
−60
−80
−100
1985
1990
1995
2000
2005
2010
2015

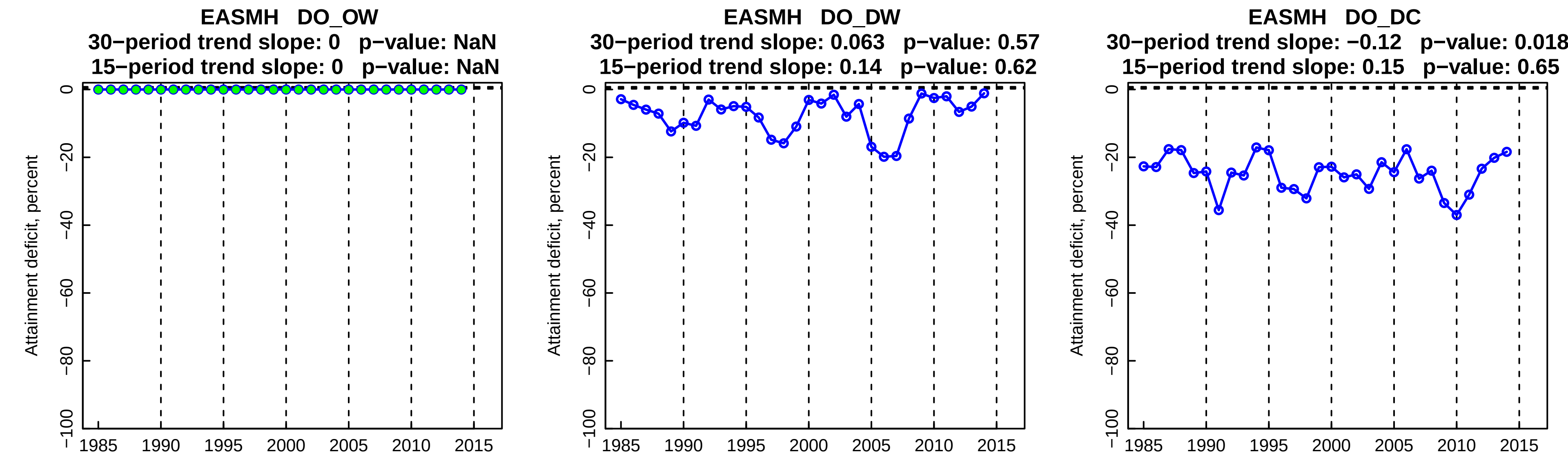

EASMH DO_OW
30−period trend slope: 0 p−value: NaN
15−period trend slope: 0 p−value: NaN
EASMH DO_DW
30−period trend slope: 0.063 p−value: 0.57
15−period trend slope: 0.14 p−value: 0.62
EASMH DO_DC
30−period trend slope: −0.12 p−value: 0.018
15−period trend slope: 0.15 p−value: 0.65
Attainment deficit, percent
0
−20
−40
−60
−80
−100
1985
1990
1995
2000
2005
2010
2015

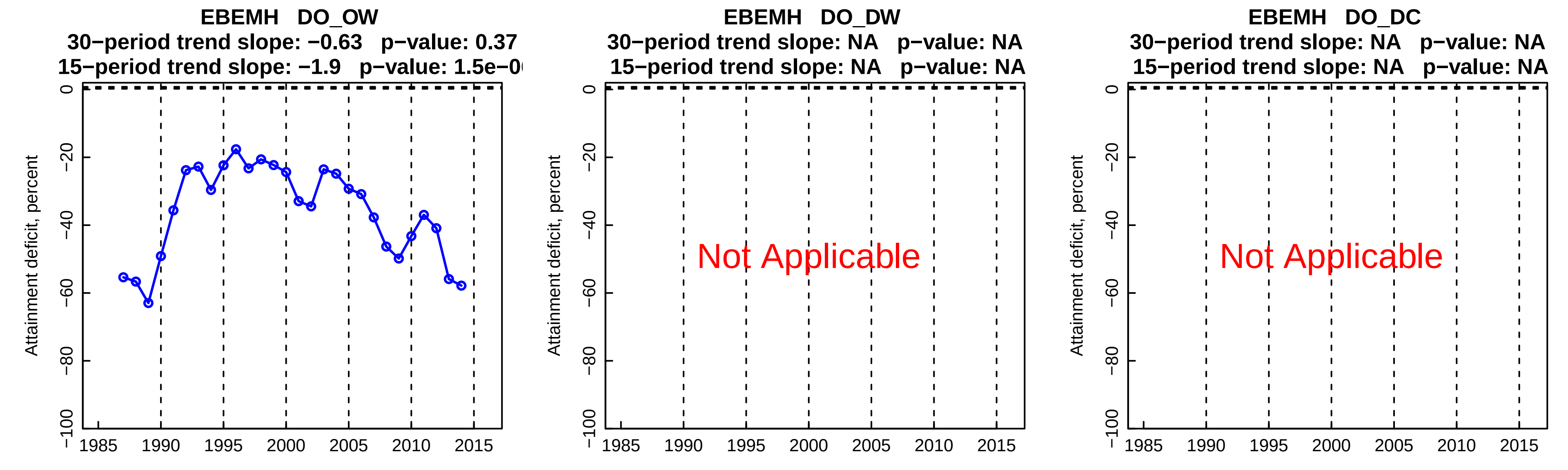
EBEMH DO_OW
30−period trend slope: −0.63 p−value: 0.37
15−period trend slope: −1.9 p−value: 1.5e−0
Attainment deficit, percent
0
−20
−40
−60
−80
−100
1985
1990
1995
2000
2005
2010
2015
EBEMH DO_DW
30−period trend slope: NA p−value: NA
15−period trend slope: NA p−value: NA
Not Applicable
EBEMH DO_DC
30−period trend slope: NA p−value: NA
15−period trend slope: NA p−value: NA
Not Applicable

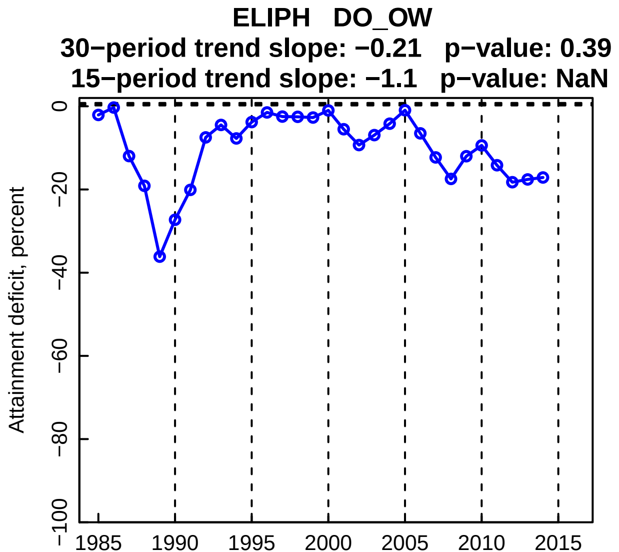
ELIPH DO_OW
30−period trend slope: −0.21 p−value: 0.39
15−period trend slope: −1.1 p−value: NaN
Attainment deficit, percent
0
−20
−40
−60
−80
−100
1985
1990
1995
2000
2005
2010
2015

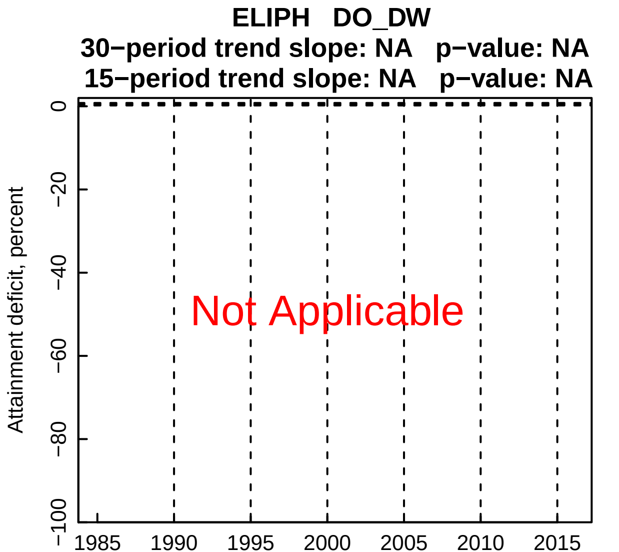
ELIPH DO_DW
30−period trend slope: NA p−value: NA
15−period trend slope: NA p−value: NA
Attainment deficit, percent
Not Applicable
0
−20
−40
−60
−80
−100
1985
1990
1995
2000
2005
2010
2015

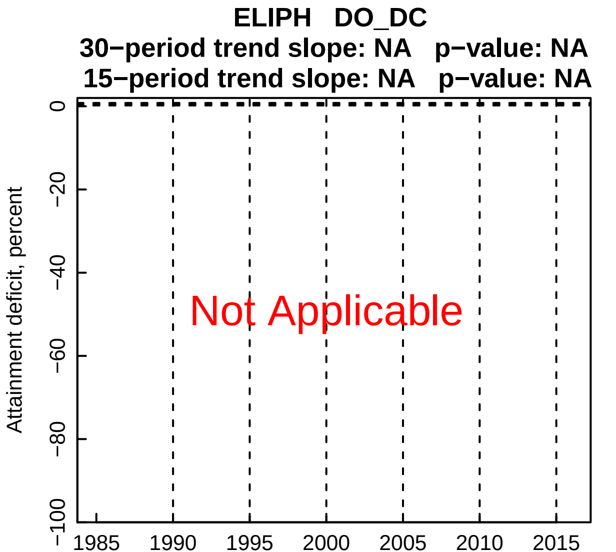
ELIPH DO_DC
30−period trend slope: NA p−value: NA
15−period trend slope: NA p−value: NA
Attainment deficit, percent
Not Applicable
0
−20
−40
−60
−80
−100
1985
1990
1995
2000
2005
2010
2015

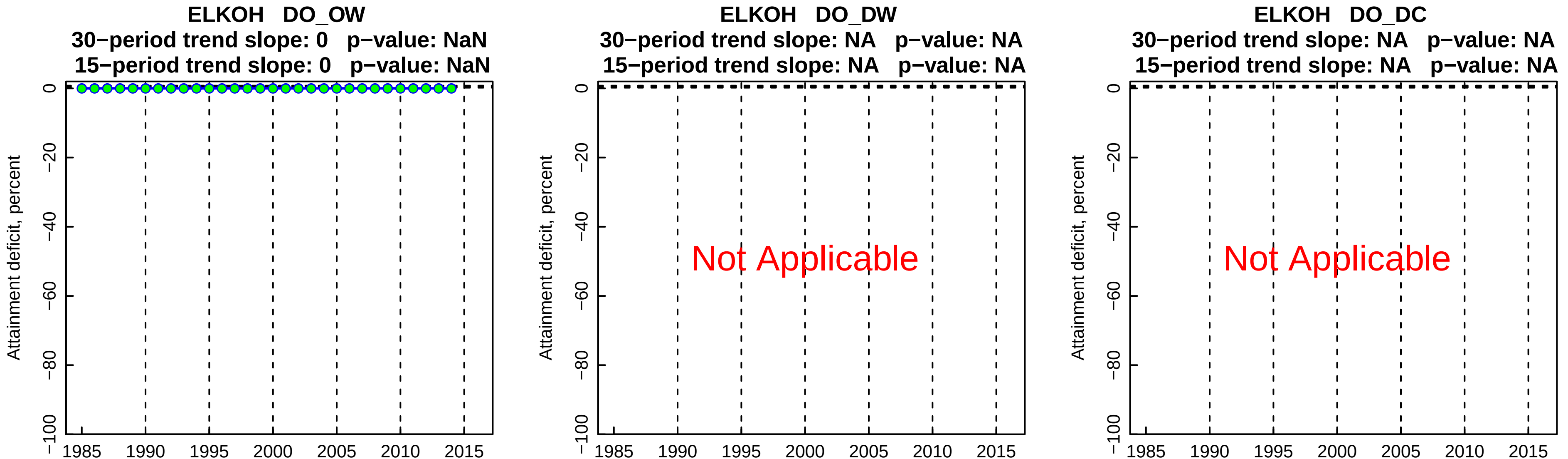
ELKOH DO_OW
30−period trend slope: 0 p−value: NaN
15−period trend slope: 0 p−value: NaN
Attainment deficit, percent
0
−20
−40
−60
−80
−100
1985
1990
1995
2000
2005
2010
2015
ELKOH DO_DW
30−period trend slope: NA p−value: NA
15−period trend slope: NA p−value: NA
Not Applicable
ELKOH DO_DC
30−period trend slope: NA p−value: NA
15−period trend slope: NA p−value: NA
Not Applicable

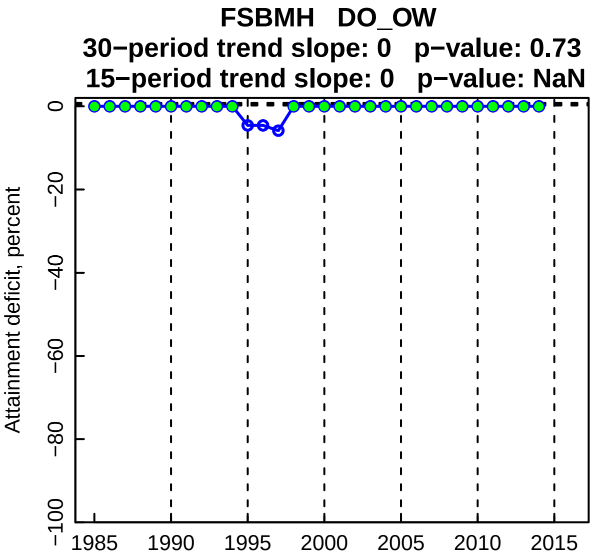
FSBMH DO_OW
30−period trend slope: 0 p−value: 0.73
15−period trend slope: 0 p−value: NaN
Attainment deficit, percent
0
−20
−40
−60
−80
−100
1985
1990
1995
2000
2005
2010
2015

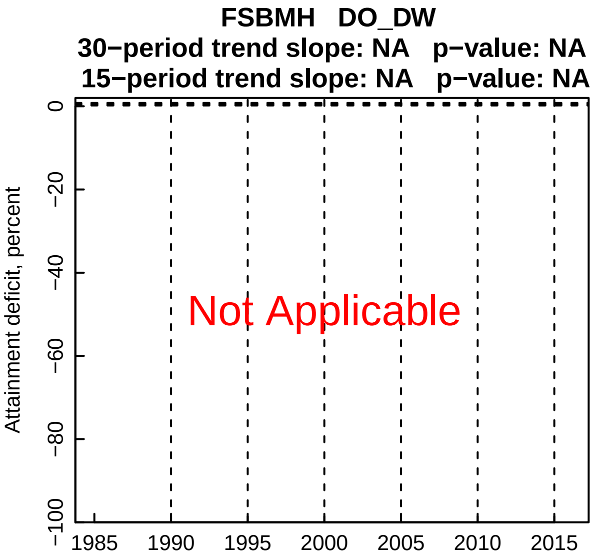
FSBMH DO_DW
30−period trend slope: NA p−value: NA
15−period trend slope: NA p−value: NA
Attainment deficit, percent
Not Applicable
0
−20
−40
−60
−80
−100
1985
1990
1995
2000
2005
2010
2015

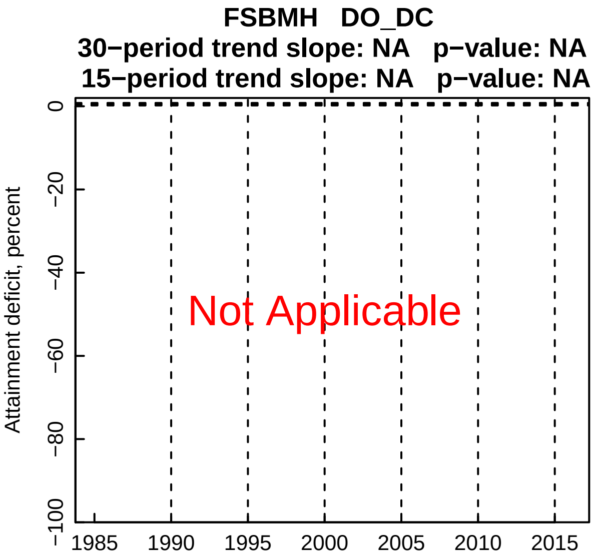
FSBMH DO_DC
30−period trend slope: NA p−value: NA
15−period trend slope: NA p−value: NA
Attainment deficit, percent
Not Applicable
0
−20
−40
−60
−80
−100
1985
1990
1995
2000
2005
2010
2015

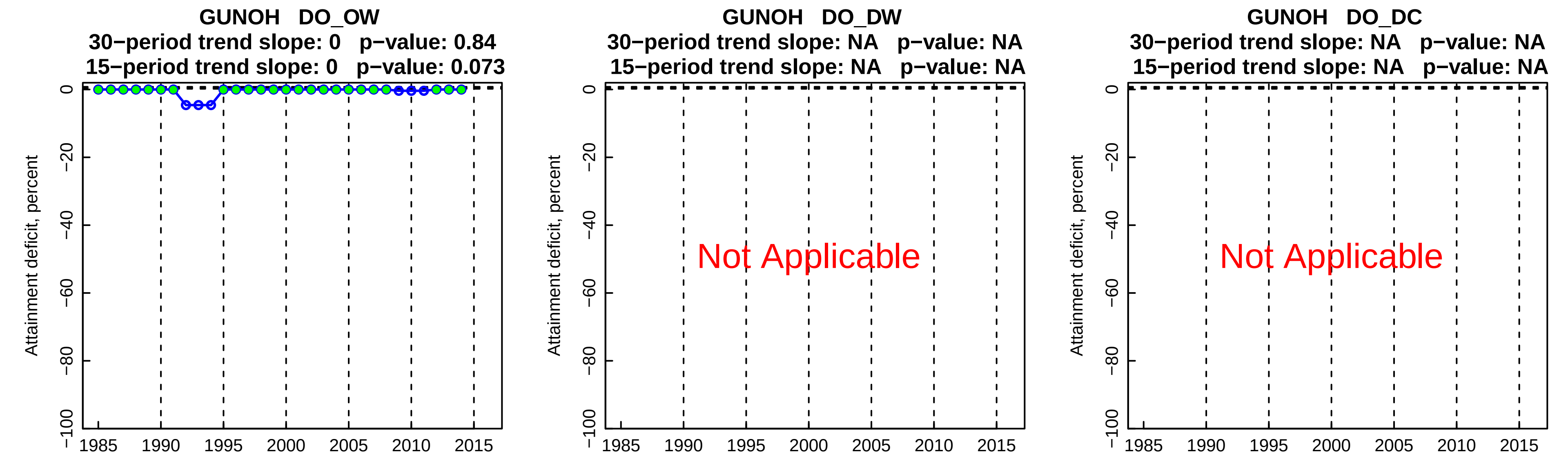
GUNOH DO_OW
30−period trend slope: 0 p−value: 0.84
15−period trend slope: 0 p−value: 0.073
Attainment deficit, percent
0
−20
−40
−60
−80
−100
1985 1990 1995 2000 2005 2010 2015
GUNOH DO_DW
30−period trend slope: NA p−value: NA
15−period trend slope: NA p−value: NA
Attainment deficit, percent
Not Applicable
GUNOH DO_DC
30−period trend slope: NA p−value: NA
15−period trend slope: NA p−value: NA
Attainment deficit, percent
Not Applicable

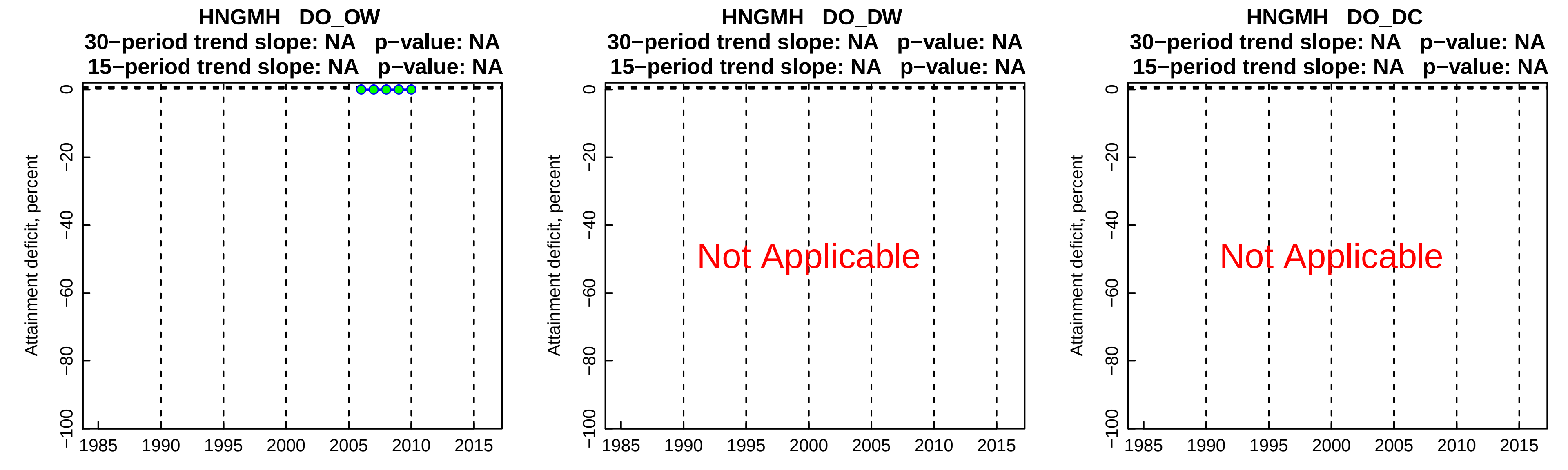
HNGMH DO_OW
30−period trend slope: NA p−value: NA
15−period trend slope: NA p−value: NA
Attainment deficit, percent
0
−20
−40
−60
−80
−100
1985
1990
1995
2000
2005
2010
2015
HNGMH DO_DW
30−period trend slope: NA p−value: NA
15−period trend slope: NA p−value: NA
Not Applicable
HNGMH DO_DC
30−period trend slope: NA p−value: NA
15−period trend slope: NA p−value: NA
Not Applicable

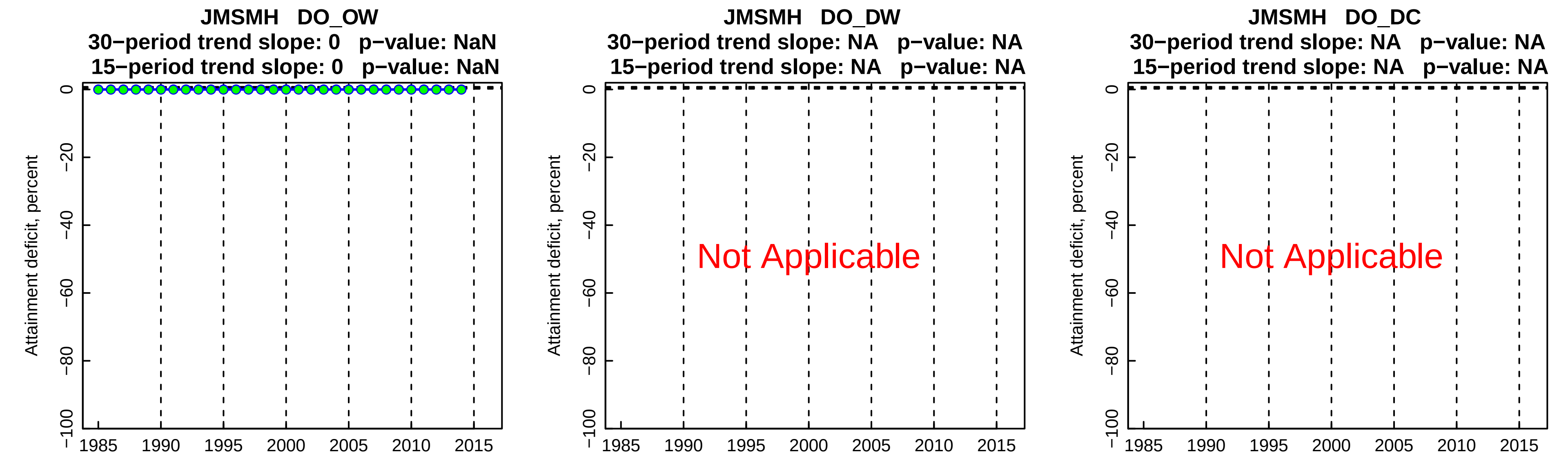

JMSMH DO_OW
30−period trend slope: 0 p−value: NaN
15−period trend slope: 0 p−value: NaN
Attainment deficit, percent
0
−20
−40
−60
−80
−100
1985 1990 1995 2000 2005 2010 2015
JMSMH DO_DW
30−period trend slope: NA p−value: NA
15−period trend slope: NA p−value: NA
Not Applicable
JMSMH DO_DC
30−period trend slope: NA p−value: NA
15−period trend slope: NA p−value: NA
Not Applicable

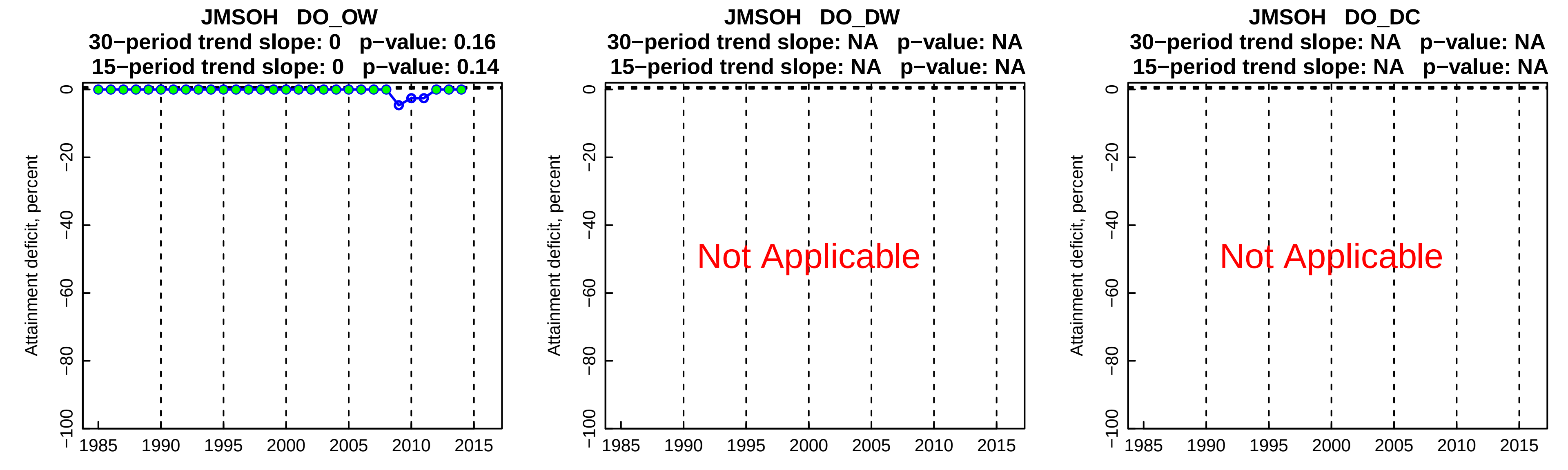

JMSOH DO_OW
30−period trend slope: 0 p−value: 0.16
15−period trend slope: 0 p−value: 0.14
Attainment deficit, percent
0
−20
−40
−60
−80
−100
1985
1990
1995
2000
2005
2010
2015
JMSOH DO_DW
30−period trend slope: NA p−value: NA
15−period trend slope: NA p−value: NA
Not Applicable
JMSOH DO_DC
30−period trend slope: NA p−value: NA
15−period trend slope: NA p−value: NA
Not Applicable

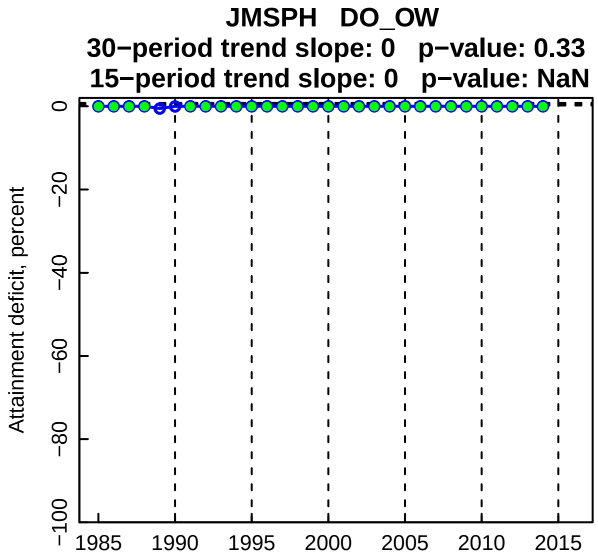
JMSPH DO_OW
30−period trend slope: 0 p−value: 0.33
15−period trend slope: 0 p−value: NaN
Attainment deficit, percent
0
−20
−40
−60
−80
−100
1985
1990
1995
2000
2005
2010
2015

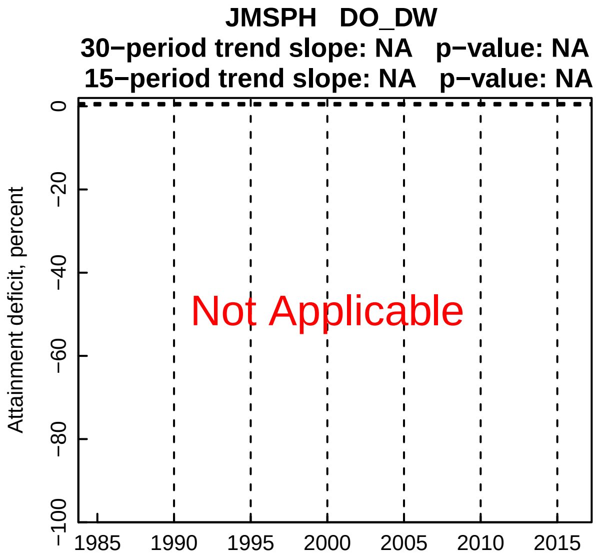
JMSPH DO_DW
30−period trend slope: NA p−value: NA
15−period trend slope: NA p−value: NA
Attainment deficit, percent
Not Applicable
0
−20
−40
−60
−80
−100
1985
1990
1995
2000
2005
2010
2015

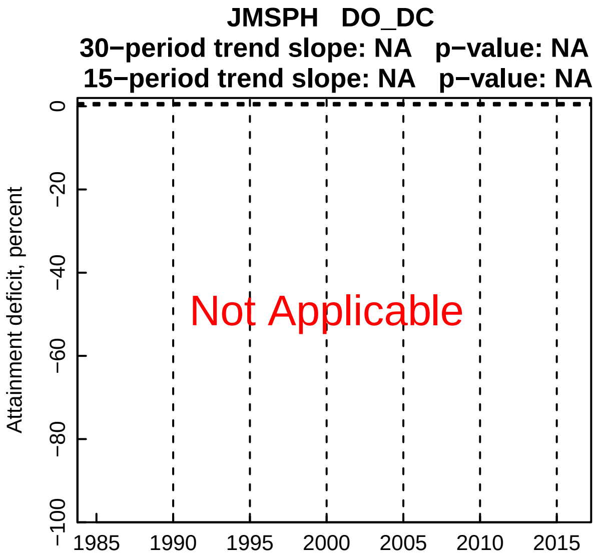
JMSPH DO_DC
30−period trend slope: NA p−value: NA
15−period trend slope: NA p−value: NA
Attainment deficit, percent
Not Applicable
0
−20
−40
−60
−80
−100
1985
1990
1995
2000
2005
2010
2015

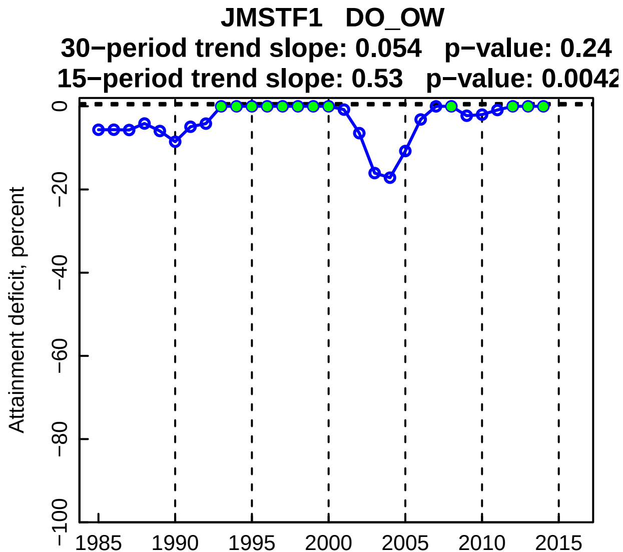
JMSTF1 DO_OW
30−period trend slope: 0.054 p−value: 0.24
15−period trend slope: 0.53 p−value: 0.0042
Attainment deficit, percent
0
−20
−40
−60
−80
−100
1985
1990
1995
2000
2005
2010
2015

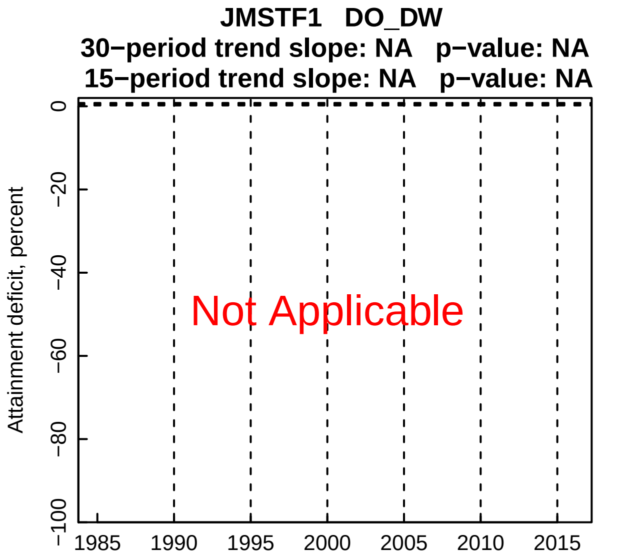
JMSTF1 DO_DW
30−period trend slope: NA p−value: NA
15−period trend slope: NA p−value: NA
Attainment deficit, percent
Not Applicable
0
−20
−40
−60
−80
−100
1985
1990
1995
2000
2005
2010
2015

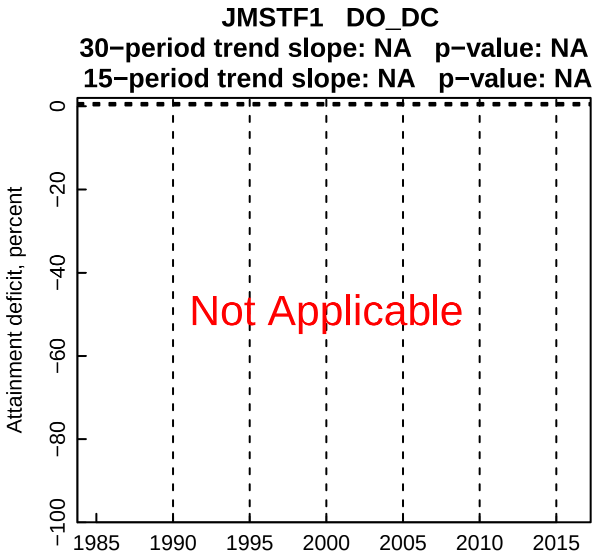
JMSTF1 DO_DC
30−period trend slope: NA p−value: NA
15−period trend slope: NA p−value: NA
Attainment deficit, percent
Not Applicable
0
−20
−40
−60
−80
−100
1985
1990
1995
2000
2005
2010
2015

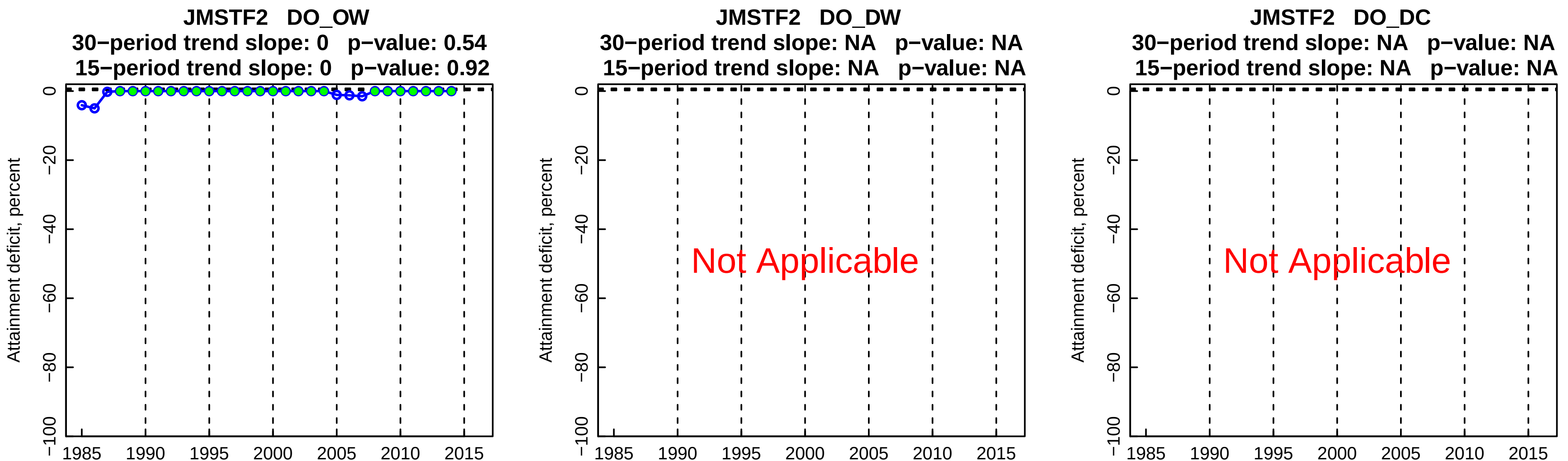

JMSTF2 DO_OW
30−period trend slope: 0 p−value: 0.54
15−period trend slope: 0 p−value: 0.92
Attainment deficit, percent
0
−20
−40
−60
−80
−100
1985
1990
1995
2000
2005
2010
2015
JMSTF2 DO_DW
30−period trend slope: NA p−value: NA
15−period trend slope: NA p−value: NA
Attainment deficit, percent
Not Applicable
JMSTF2 DO_DC
30−period trend slope: NA p−value: NA
15−period trend slope: NA p−value: NA
Attainment deficit, percent
Not Applicable

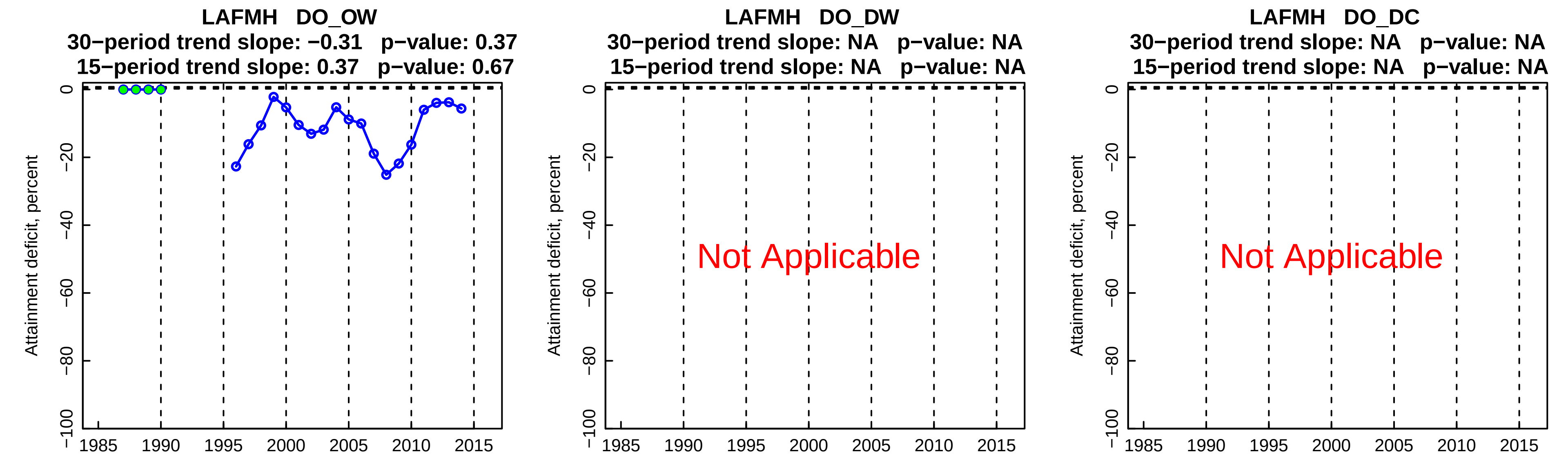
LAFMH DO_OW
30−period trend slope: −0.31 p−value: 0.37
15−period trend slope: 0.37 p−value: 0.67
Attainment deficit, percent
LAFMH DO_DW
30−period trend slope: NA p−value: NA
15−period trend slope: NA p−value: NA
Not Applicable
LAFMH DO_DC
30−period trend slope: NA p−value: NA
15−period trend slope: NA p−value: NA
Not Applicable
0
−20
−40
−60
−80
−100
1985
1990
1995
2000
2005
2010
2015

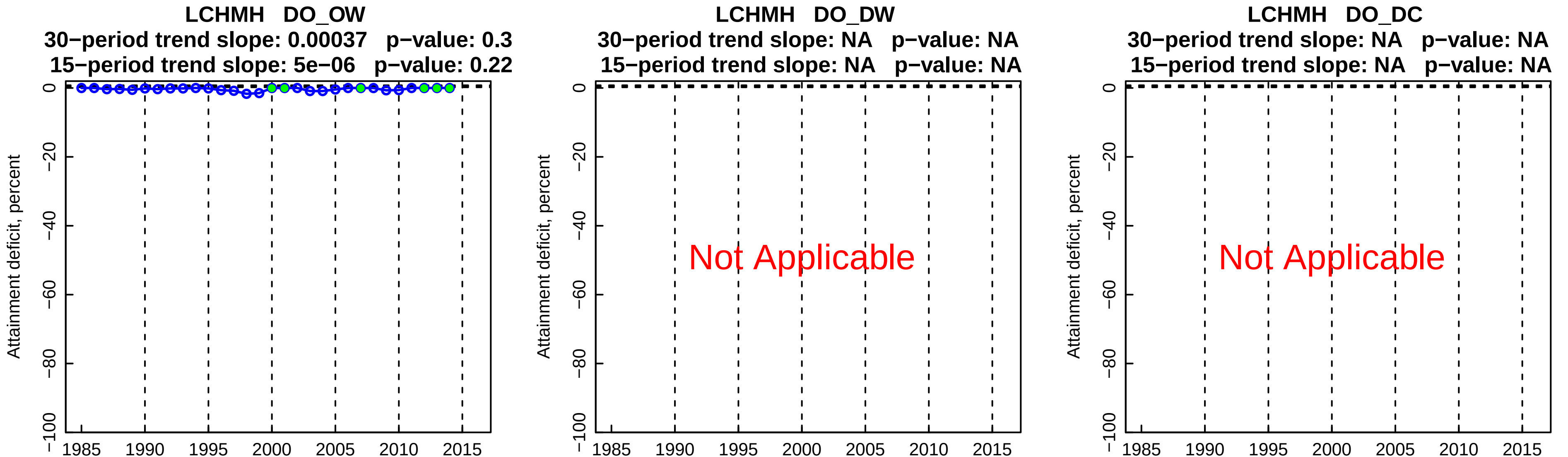

LCHMH DO_OW
30−period trend slope: 0.00037 p−value: 0.3
15−period trend slope: 5e−06 p−value: 0.22
Attainment deficit, percent
0
−20
−40
−60
−80
−100
1985
1990
1995
2000
2005
2010
2015
LCHMH DO_DW
30−period trend slope: NA p−value: NA
15−period trend slope: NA p−value: NA
Not Applicable
LCHMH DO_DC
30−period trend slope: NA p−value: NA
15−period trend slope: NA p−value: NA
Not Applicable

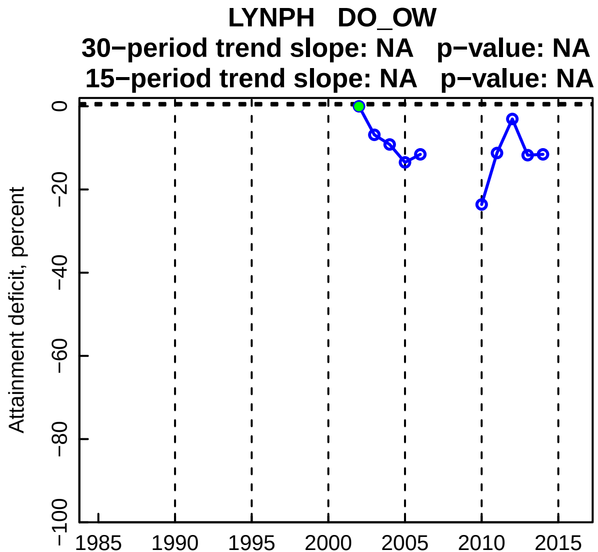
LYNPH DO_OW
30−period trend slope: NA p−value: NA
15−period trend slope: NA p−value: NA
Attainment deficit, percent
0
−20
−40
−60
−80
−100
1985
1990
1995
2000
2005
2010
2015

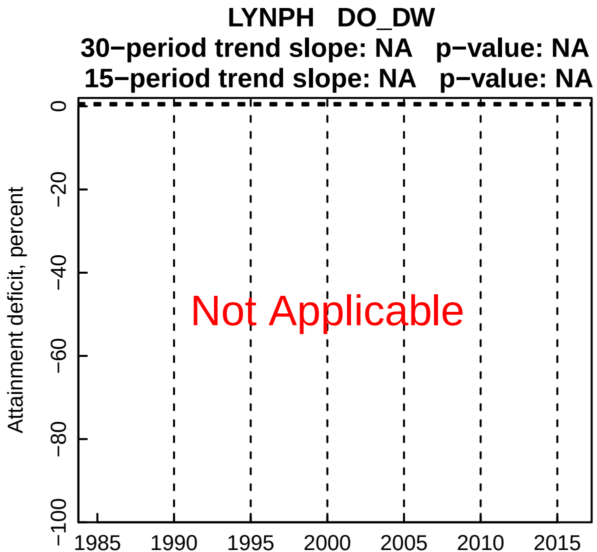
LYNPH DO_DW
30−period trend slope: NA p−value: NA
15−period trend slope: NA p−value: NA
Attainment deficit, percent
Not Applicable
0
−20
−40
−60
−80
−100
1985
1990
1995
2000
2005
2010
2015

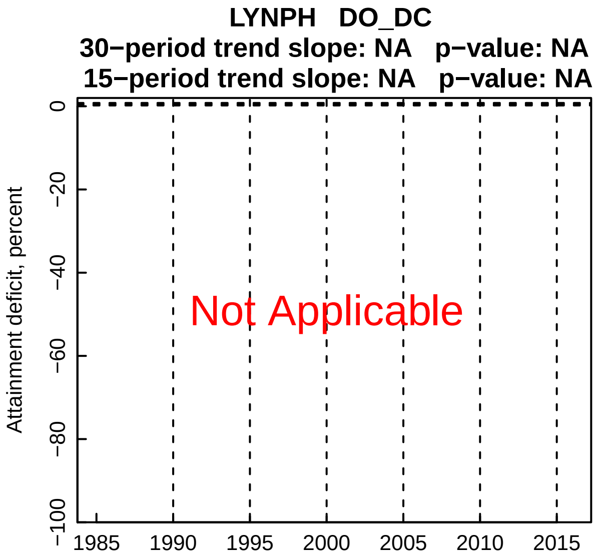
LYNPH DO_DC
30−period trend slope: NA p−value: NA
15−period trend slope: NA p−value: NA
Attainment deficit, percent
Not Applicable
0
−20
−40
−60
−80
−100
1985
1990
1995
2000
2005
2010
2015

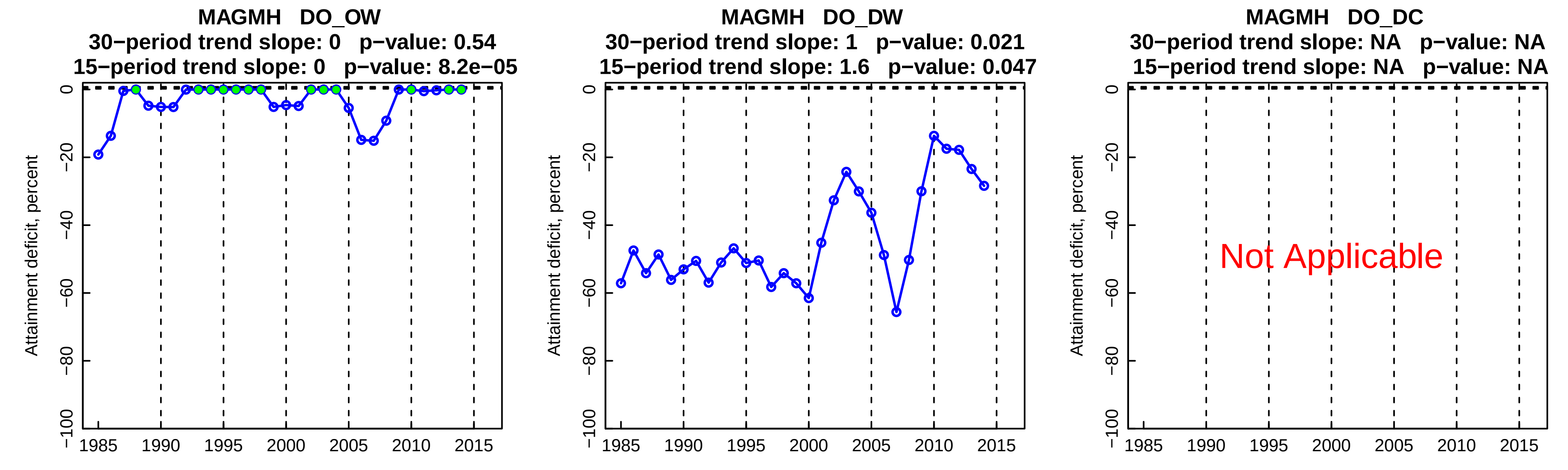
MAGMH DO_OW
30−period trend slope: 0 p−value: 0.54
15−period trend slope: 0 p−value: 8.2e−05
MAGMH DO_DW
30−period trend slope: 1 p−value: 0.021
15−period trend slope: 1.6 p−value: 0.047
MAGMH DO_DC
30−period trend slope: NA p−value: NA
15−period trend slope: NA p−value: NA
Attainment deficit, percent
0
−20
−40
−60
−80
−100
1985
1990
1995
2000
2005
2010
2015
Not Applicable

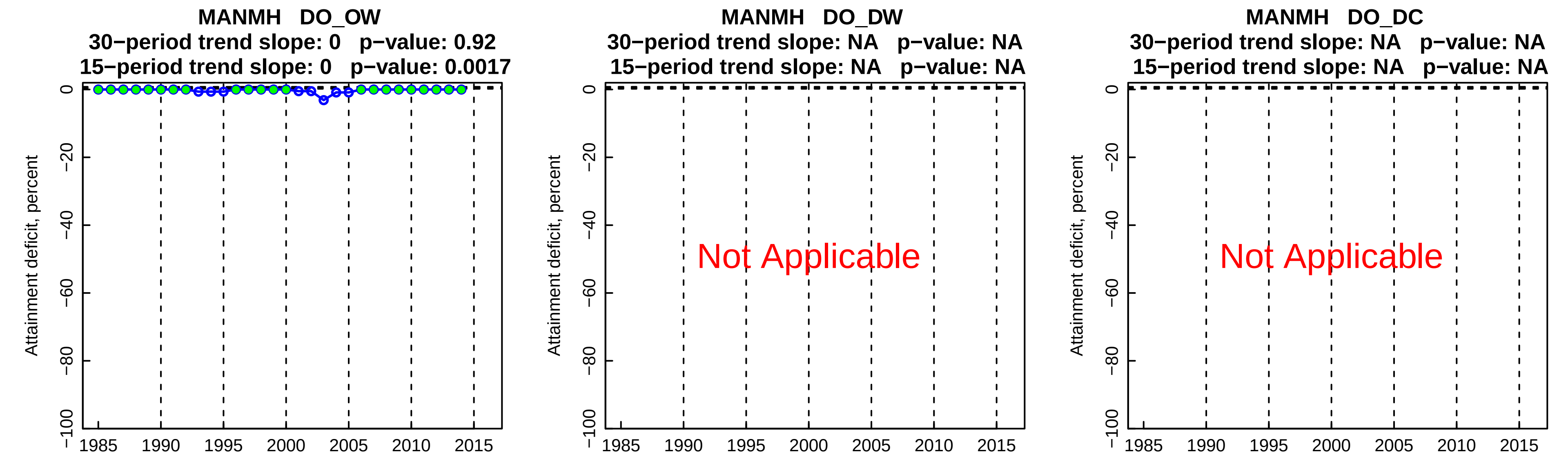
MANMH DO_OW
30−period trend slope: 0 p−value: 0.92
15−period trend slope: 0 p−value: 0.0017
MANMH DO_DW
30−period trend slope: NA p−value: NA
15−period trend slope: NA p−value: NA
Not Applicable
MANMH DO_DC
30−period trend slope: NA p−value: NA
15−period trend slope: NA p−value: NA
Not Applicable
Attainment deficit, percent
0
−20
−40
−60
−80
−100
1985
1990
1995
2000
2005
2010
2015

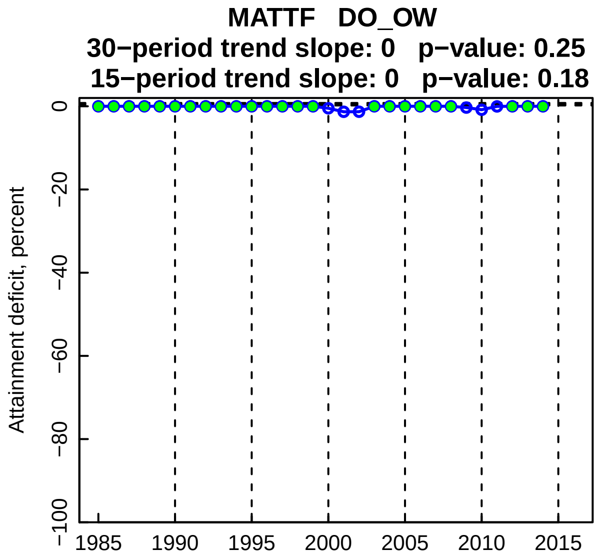

MATTF DO_OW
30−period trend slope: 0 p−value: 0.25
15−period trend slope: 0 p−value: 0.18
Attainment deficit, percent
0
−20
−40
−60
−80
−100
1985
1990
1995
2000
2005
2010
2015

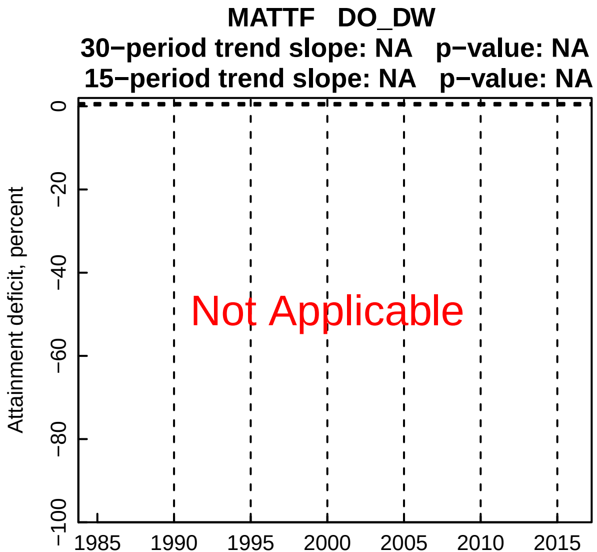

MATTF DO_DW
30−period trend slope: NA p−value: NA
15−period trend slope: NA p−value: NA
Attainment deficit, percent
Not Applicable
0
−20
−40
−60
−80
−100
1985
1990
1995
2000
2005
2010
2015

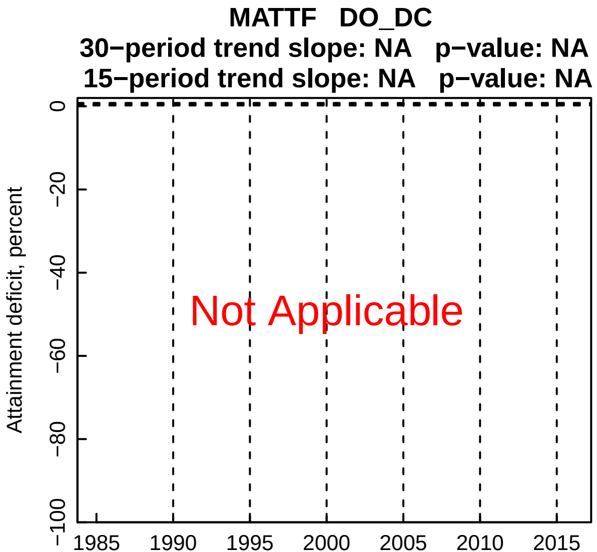

MATTF DO_DC
30−period trend slope: NA p−value: NA
15−period trend slope: NA p−value: NA
Attainment deficit, percent
Not Applicable
0
−20
−40
−60
−80
−100
1985
1990
1995
2000
2005
2010
2015

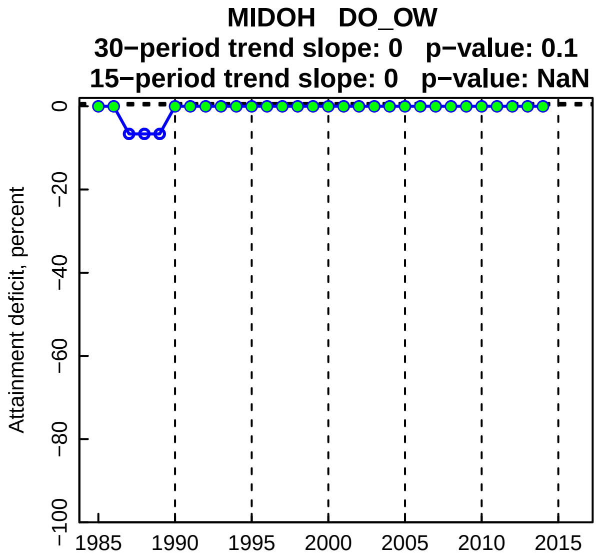
MIDOH DO_OW
30−period trend slope: 0 p−value: 0.1
15−period trend slope: 0 p−value: NaN
Attainment deficit, percent
0
−20
−40
−60
−80
−100
1985
1990
1995
2000
2005
2010
2015

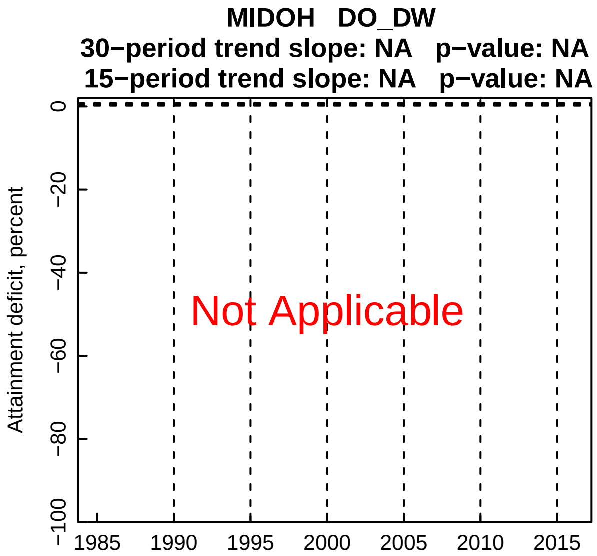
MIDOH DO_DW
30−period trend slope: NA p−value: NA
15−period trend slope: NA p−value: NA
Attainment deficit, percent
Not Applicable
0
−20
−40
−60
−80
−100
1985
1990
1995
2000
2005
2010
2015

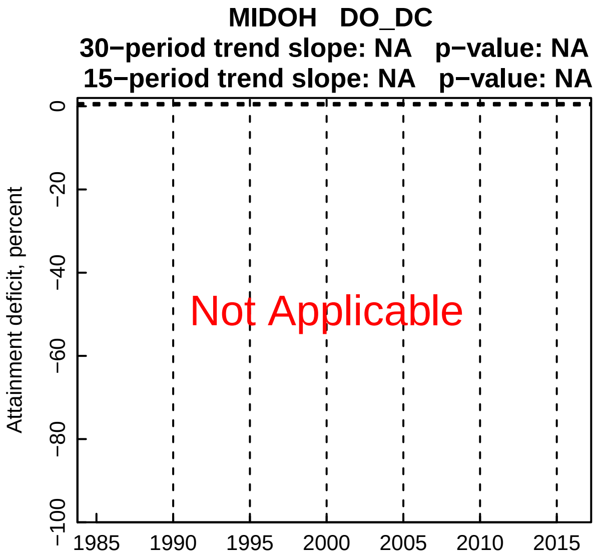
MIDOH DO_DC
30−period trend slope: NA p−value: NA
15−period trend slope: NA p−value: NA
Attainment deficit, percent
Not Applicable
0
−20
−40
−60
−80
−100
1985
1990
1995
2000
2005
2010
2015

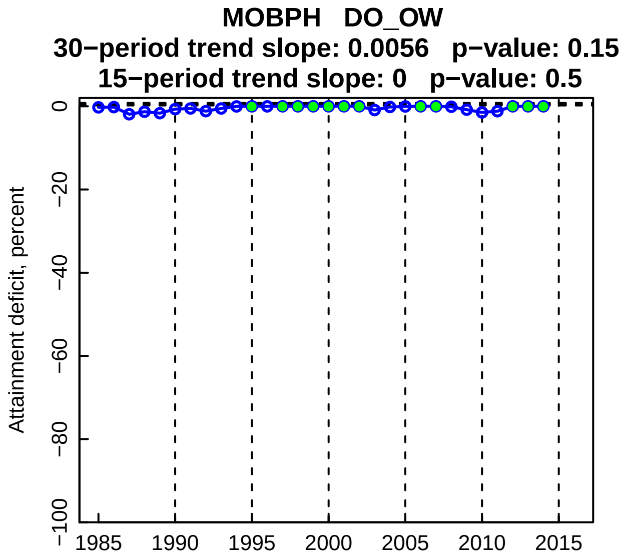
MOBPH DO_OW
30−period trend slope: 0.0056 p−value: 0.15
15−period trend slope: 0 p−value: 0.5
Attainment deficit, percent
0
−20
−40
−60
−80
−100
1985
1990
1995
2000
2005
2010
2015

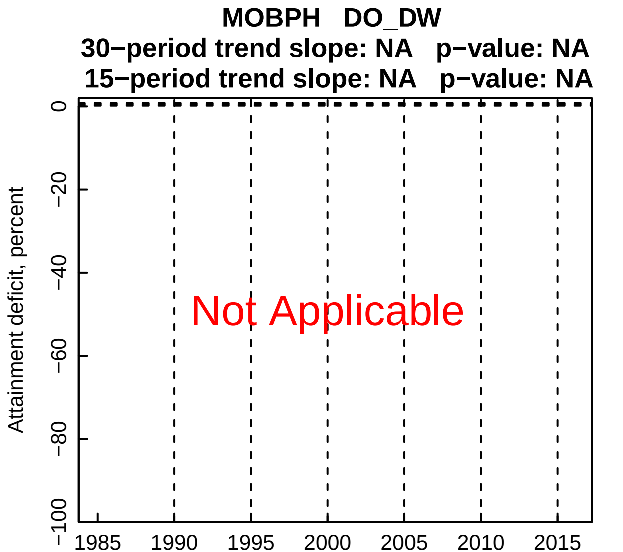
MOBPH DO_DW
30−period trend slope: NA p−value: NA
15−period trend slope: NA p−value: NA
Attainment deficit, percent
0
−20
−40
−60
−80
−100
Not Applicable
1985
1990
1995
2000
2005
2010
2015

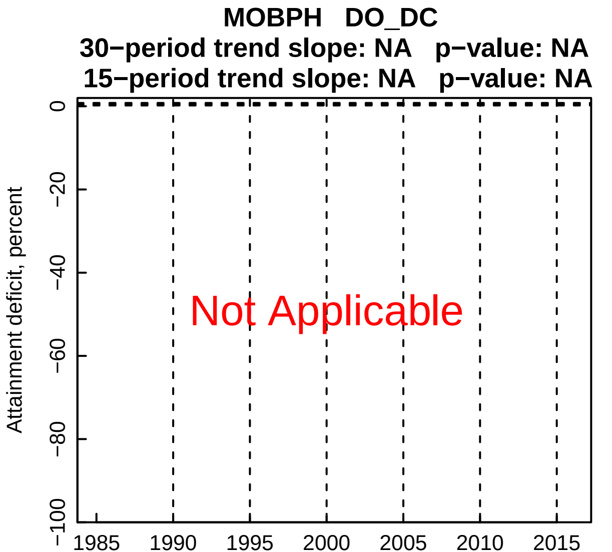
MOBPH DO_DC
30−period trend slope: NA p−value: NA
15−period trend slope: NA p−value: NA
Attainment deficit, percent
0
−20
−40
−60
−80
−100
Not Applicable
1985
1990
1995
2000
2005
2010
2015

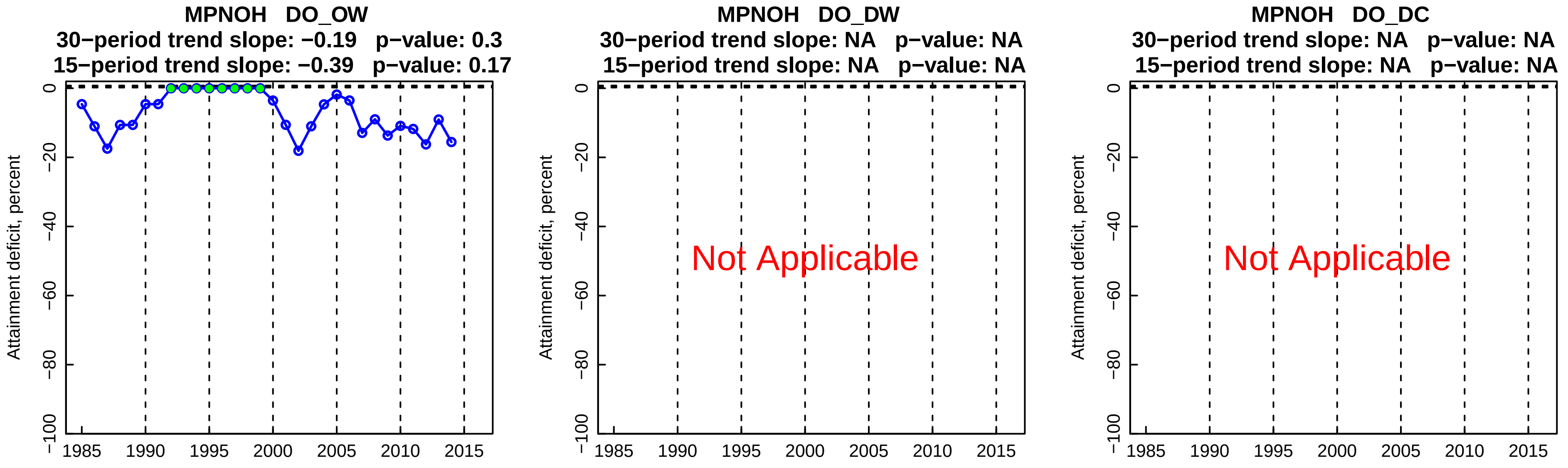
MPNOH DO_OW
30−period trend slope: −0.19 p−value: 0.3
15−period trend slope: −0.39 p−value: 0.17
Attainment deficit, percent
MPNOH DO_DW
30−period trend slope: NA p−value: NA
15−period trend slope: NA p−value: NA
Not Applicable
MPNOH DO_DC
30−period trend slope: NA p−value: NA
15−period trend slope: NA p−value: NA
Not Applicable
0
−20
−40
−60
−80
−100
1985
1990
1995
2000
2005
2010
2015

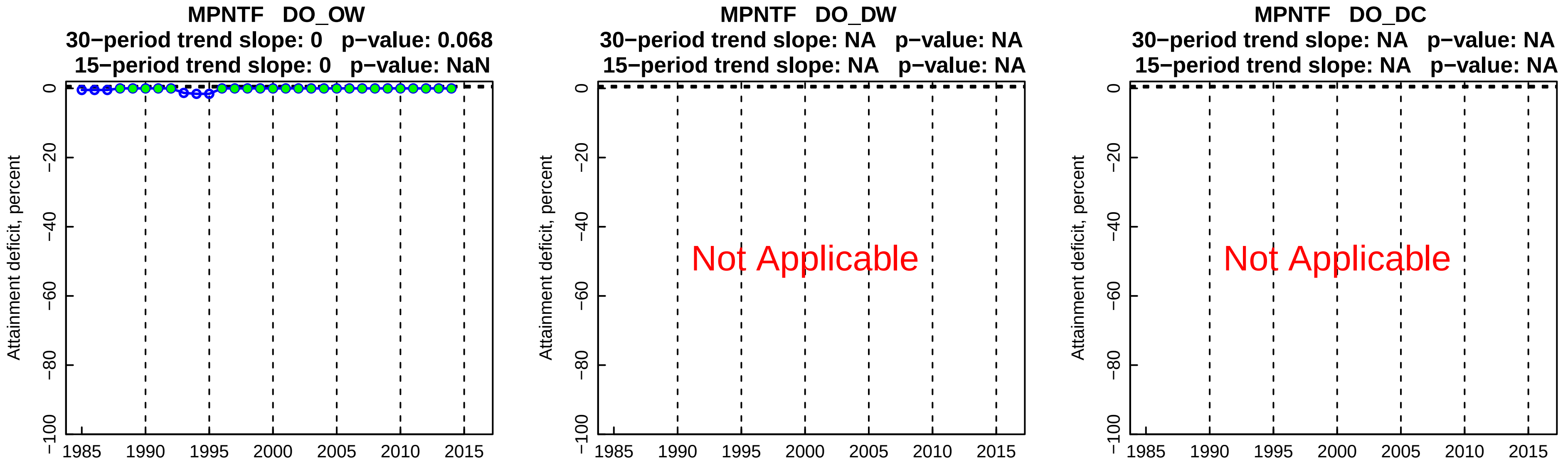
MPNTF DO_OW
30−period trend slope: 0 p−value: 0.068
15−period trend slope: 0 p−value: NaN
Attainment deficit, percent
0
−20
−40
−60
−80
−100
1985
1990
1995
2000
2005
2010
2015
MPNTF DO_DW
30−period trend slope: NA p−value: NA
15−period trend slope: NA p−value: NA
Not Applicable
MPNTF DO_DC
30−period trend slope: NA p−value: NA
15−period trend slope: NA p−value: NA
Not Applicable

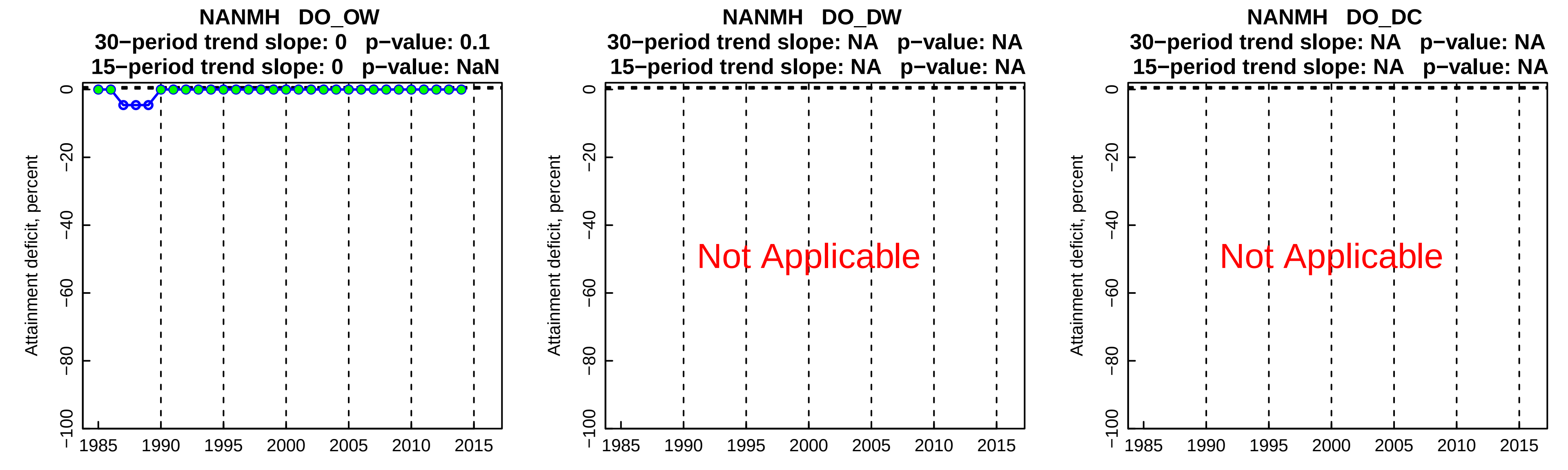
NANMH DO_OW
30−period trend slope: 0 p−value: 0.1
15−period trend slope: 0 p−value: NaN
NANMH DO_DW
30−period trend slope: NA p−value: NA
15−period trend slope: NA p−value: NA
NANMH DO_DC
30−period trend slope: NA p−value: NA
15−period trend slope: NA p−value: NA
Attainment deficit, percent
0
−20
−40
−60
−80
−100
1985
1990
1995
2000
2005
2010
2015
Not Applicable
Not Applicable

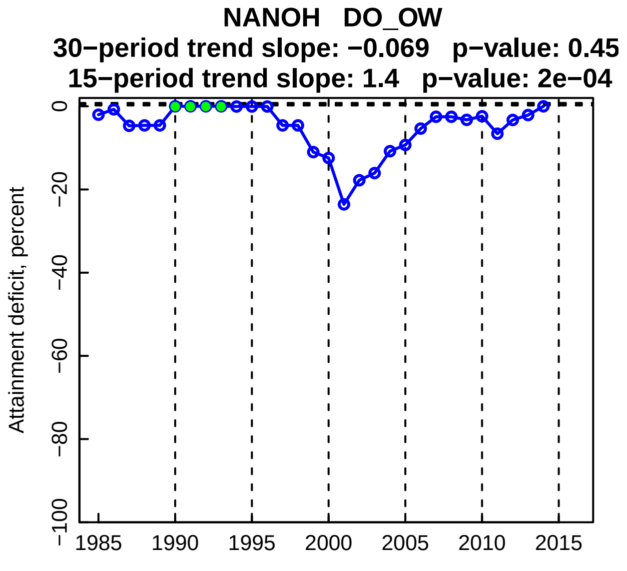
NANOH DO_OW
30−period trend slope: −0.069 p−value: 0.45
15−period trend slope: 1.4 p−value: 2e−04
Attainment deficit, percent
0
−20
−40
−60
−80
−100
1985
1990
1995
2000
2005
2010
2015

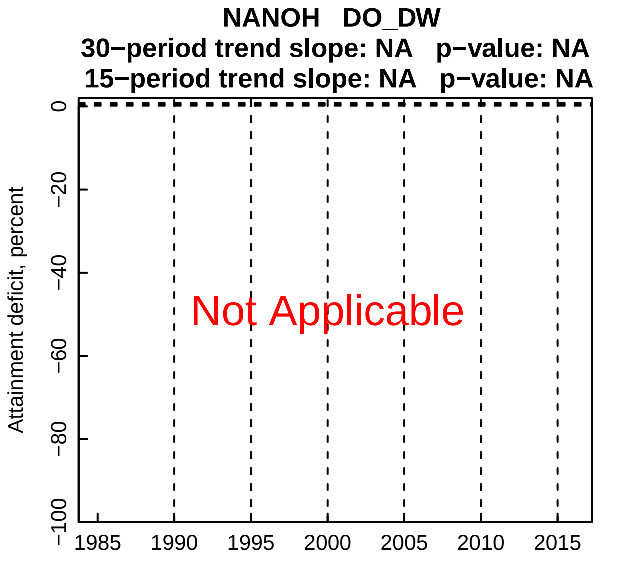
NANOH DO_DW
30−period trend slope: NA p−value: NA
15−period trend slope: NA p−value: NA
Attainment deficit, percent
0
−20
−40
−60
−80
−100
Not Applicable
1985
1990
1995
2000
2005
2010
2015

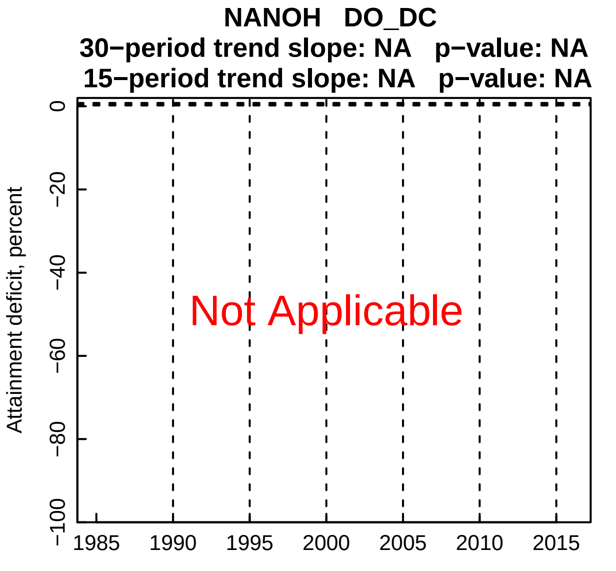
NANOH DO_DC
30−period trend slope: NA p−value: NA
15−period trend slope: NA p−value: NA
Attainment deficit, percent
0
−20
−40
−60
−80
−100
Not Applicable
1985
1990
1995
2000
2005
2010
2015

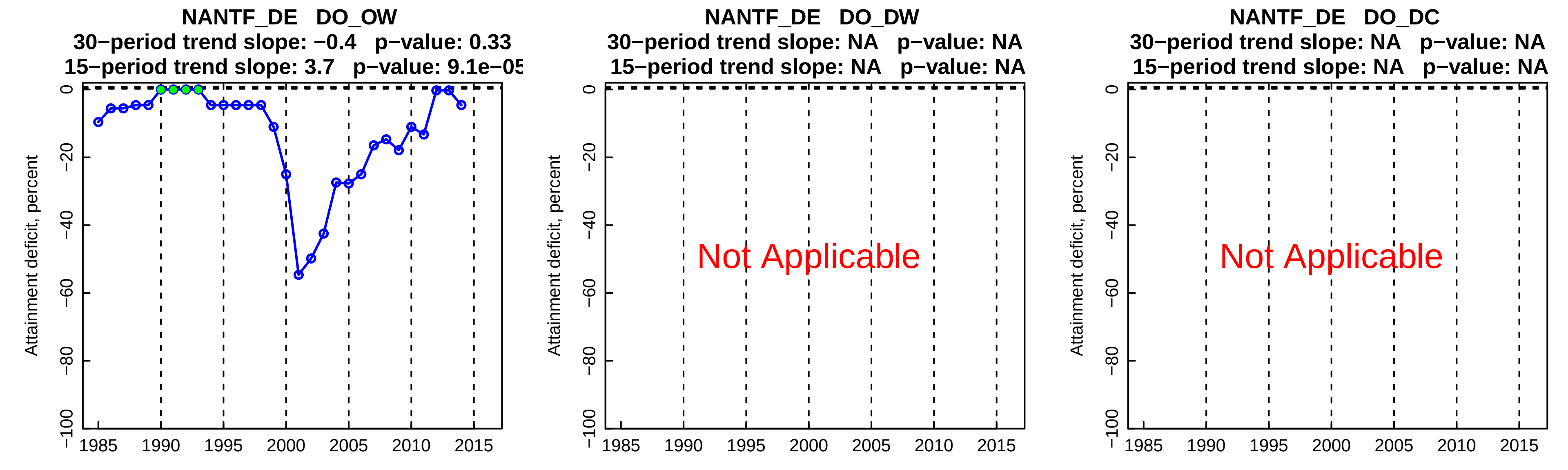
NANTF_DE DO_OW
30−period trend slope: −0.4 p−value: 0.33
15−period trend slope: 3.7 p−value: 9.1e−05
Attainment deficit, percent
0
−20
−40
−60
−80
−100
1985
1990
1995
2000
2005
2010
2015
NANTF_DE DO_DW
30−period trend slope: NA p−value: NA
15−period trend slope: NA p−value: NA
Not Applicable
NANTF_DE DO_DC
30−period trend slope: NA p−value: NA
15−period trend slope: NA p−value: NA
Not Applicable

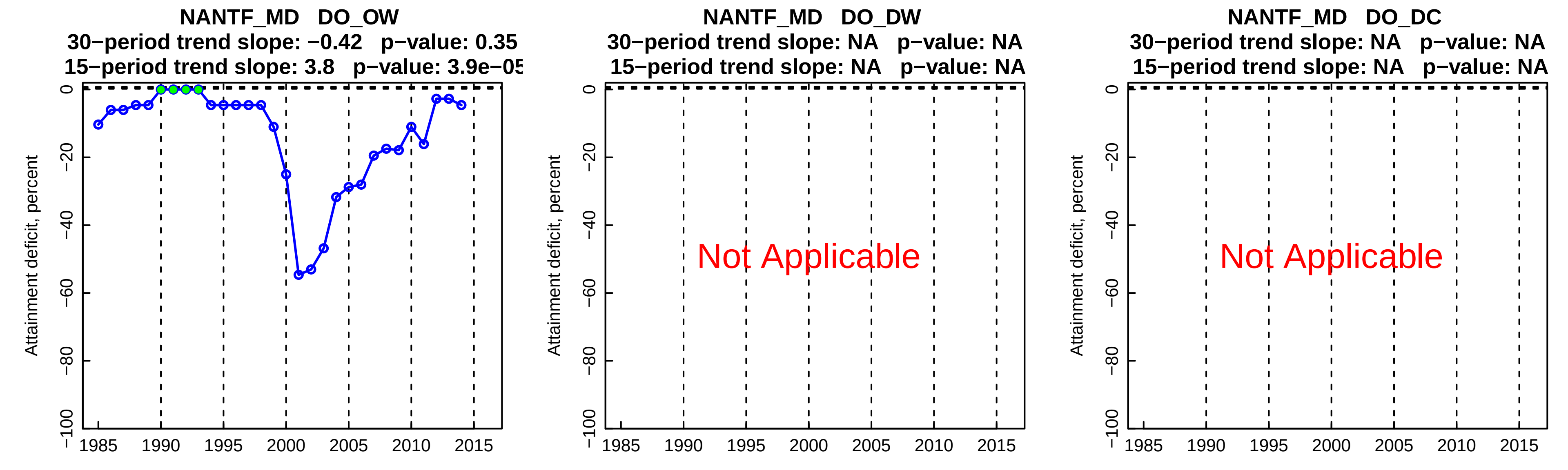

NANTF_MD DO_OW
30−period trend slope: −0.42 p−value: 0.35
15−period trend slope: 3.8 p−value: 3.9e−05
Attainment deficit, percent
0
−20
−40
−60
−80
−100
1985
1990
1995
2000
2005
2010
2015
NANTF_MD DO_DW
30−period trend slope: NA p−value: NA
15−period trend slope: NA p−value: NA
Not Applicable
NANTF_MD DO_DC
30−period trend slope: NA p−value: NA
15−period trend slope: NA p−value: NA
Not Applicable

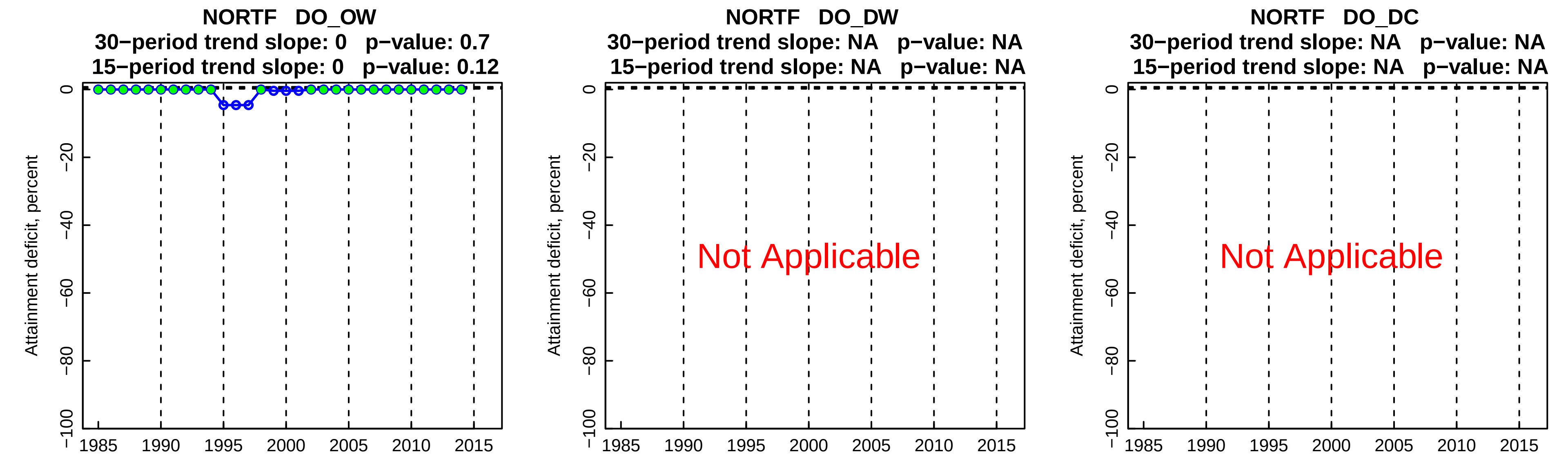

NORTF DO_OW
30−period trend slope: 0 p−value: 0.7
15−period trend slope: 0 p−value: 0.12
Attainment deficit, percent
0
−20
−40
−60
−80
−100
1985
1990
1995
2000
2005
2010
2015
NORTF DO_DW
30−period trend slope: NA p−value: NA
15−period trend slope: NA p−value: NA
Not Applicable
NORTF DO_DC
30−period trend slope: NA p−value: NA
15−period trend slope: NA p−value: NA
Not Applicable

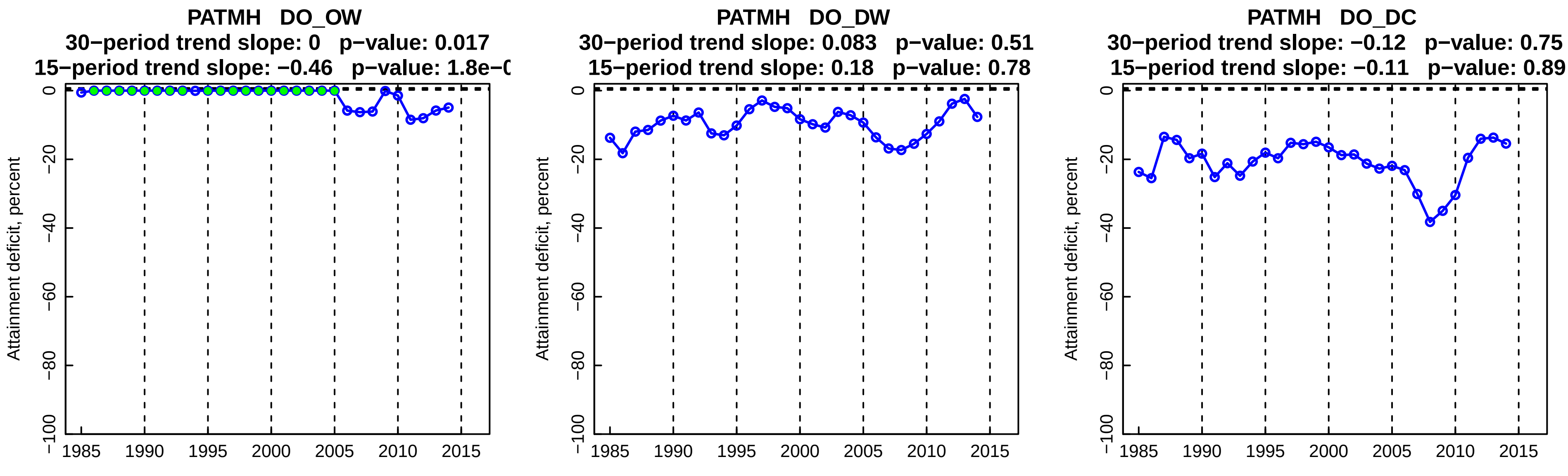
PATMH DO_OW
30−period trend slope: 0 p−value: 0.017
15−period trend slope: −0.46 p−value: 1.8e−0
PATMH DO_DW
30−period trend slope: 0.083 p−value: 0.51
15−period trend slope: 0.18 p−value: 0.78
PATMH DO_DC
30−period trend slope: −0.12 p−value: 0.75
15−period trend slope: −0.11 p−value: 0.89
Attainment deficit, percent
0
−20
−40
−60
−80
−100
1985
1990
1995
2000
2005
2010
2015

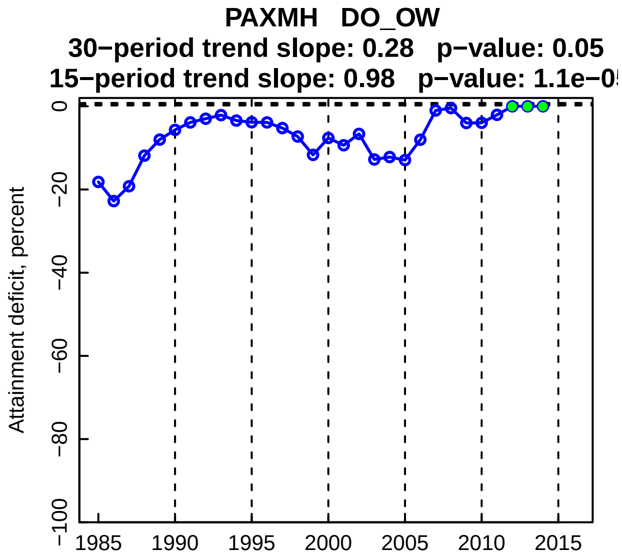
PAXMH DO_OW
30−period trend slope: 0.28 p−value: 0.05
15−period trend slope: 0.98 p−value: 1.1e−0
Attainment deficit, percent
0
−20
−40
−60
−80
−100
1985
1990
1995
2000
2005
2010
2015

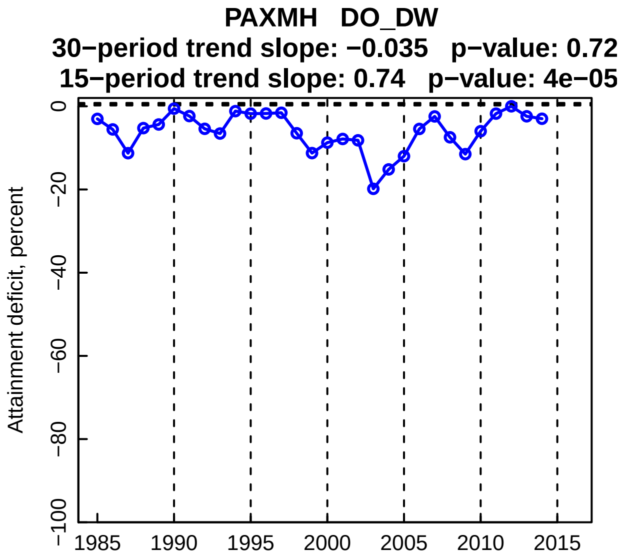
PAXMH DO_DW
30−period trend slope: −0.035 p−value: 0.72
15−period trend slope: 0.74 p−value: 4e−05
Attainment deficit, percent
0
−20
−40
−60
−80
−100
1985
1990
1995
2000
2005
2010
2015

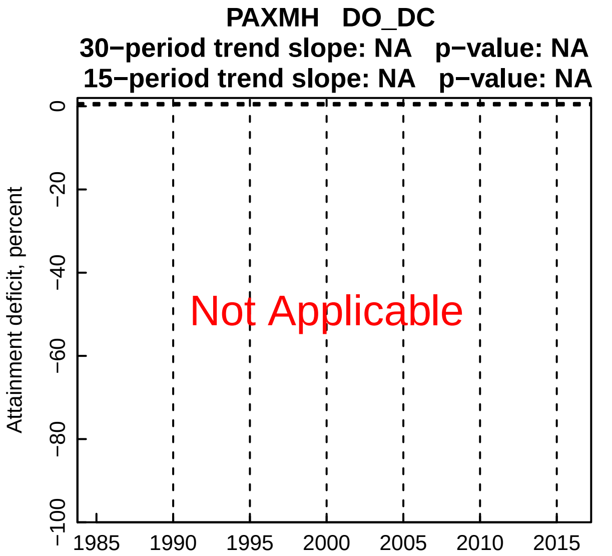
PAXMH DO_DC
30−period trend slope: NA p−value: NA
15−period trend slope: NA p−value: NA
Attainment deficit, percent
Not Applicable
0
−20
−40
−60
−80
−100
1985
1990
1995
2000
2005
2010
2015

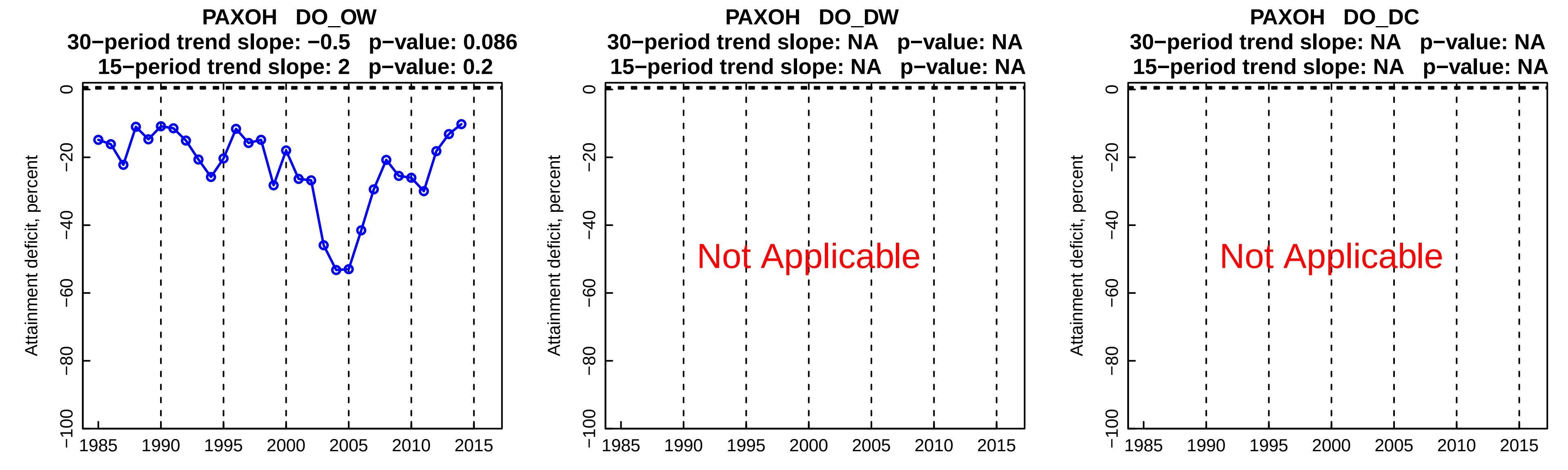
PAXOH DO_OW
30−period trend slope: −0.5 p−value: 0.086
15−period trend slope: 2 p−value: 0.2
Attainment deficit, percent
0
−20
−40
−60
−80
−100
1985
1990
1995
2000
2005
2010
2015
PAXOH DO_DW
30−period trend slope: NA p−value: NA
15−period trend slope: NA p−value: NA
Not Applicable
PAXOH DO_DC
30−period trend slope: NA p−value: NA
15−period trend slope: NA p−value: NA
Not Applicable

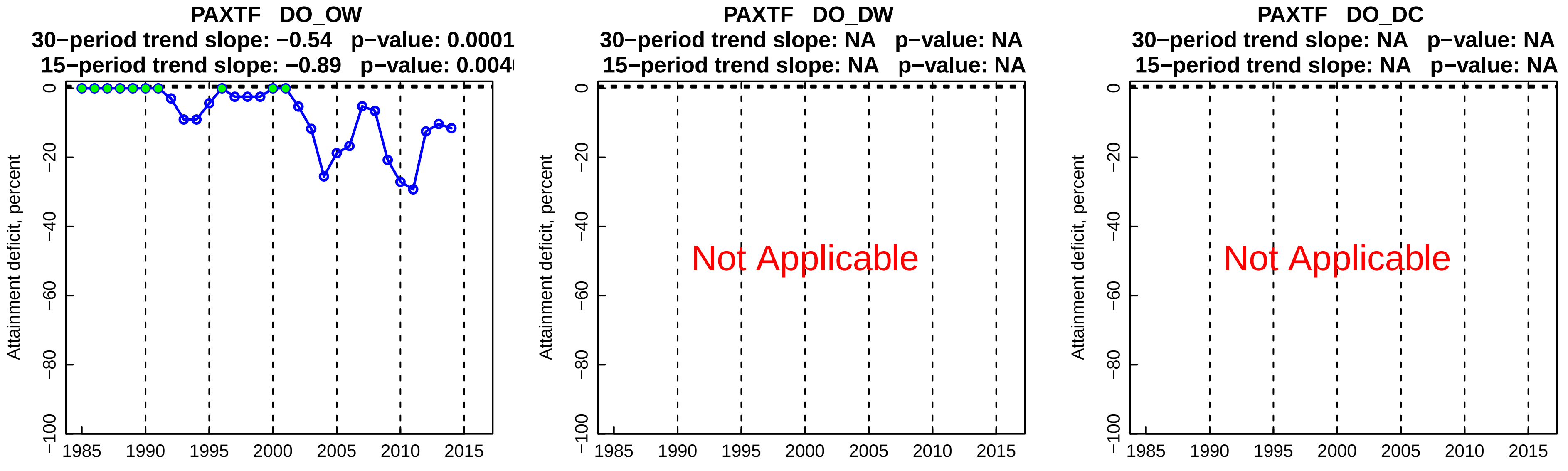

PAXTF DO_OW
30−period trend slope: −0.54 p−value: 0.0001
15−period trend slope: −0.89 p−value: 0.004
Attainment deficit, percent
0
−20
−40
−60
−80
−100
1985
1990
1995
2000
2005
2010
2015
PAXTF DO_DW
30−period trend slope: NA p−value: NA
15−period trend slope: NA p−value: NA
Not Applicable
PAXTF DO_DC
30−period trend slope: NA p−value: NA
15−period trend slope: NA p−value: NA
Not Applicable

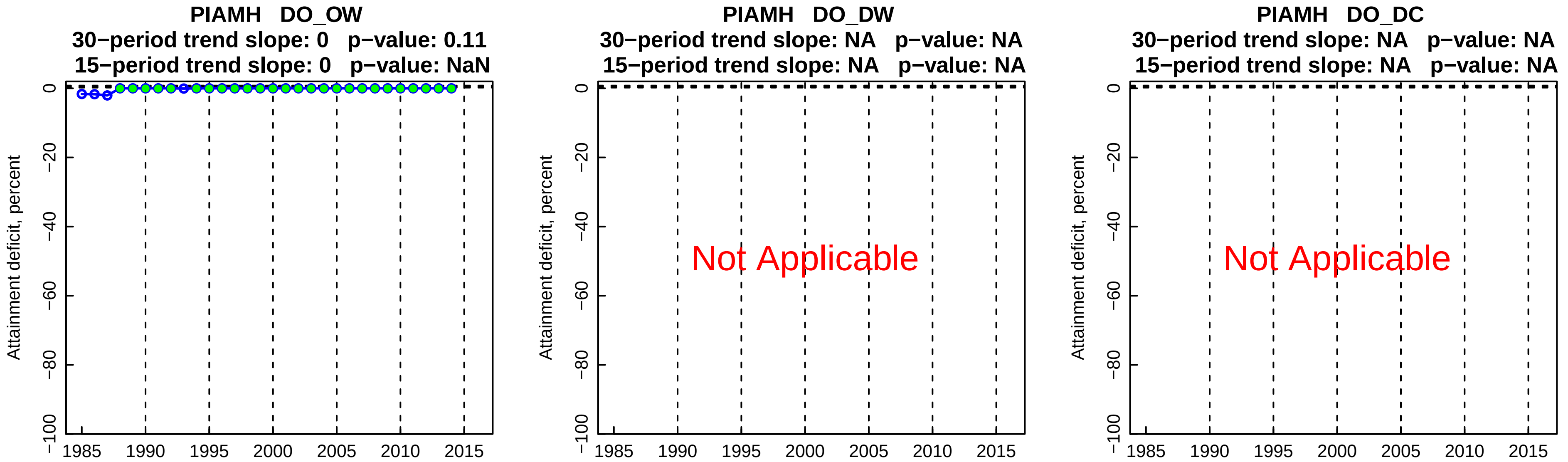
PIAMH DO_OW
30−period trend slope: 0 p−value: 0.11
15−period trend slope: 0 p−value: NaN
PIAMH DO_DW
30−period trend slope: NA p−value: NA
15−period trend slope: NA p−value: NA
PIAMH DO_DC
30−period trend slope: NA p−value: NA
15−period trend slope: NA p−value: NA
Attainment deficit, percent
0
−20
−40
−60
−80
−100
1985
1990
1995
2000
2005
2010
2015
Not Applicable
Not Applicable

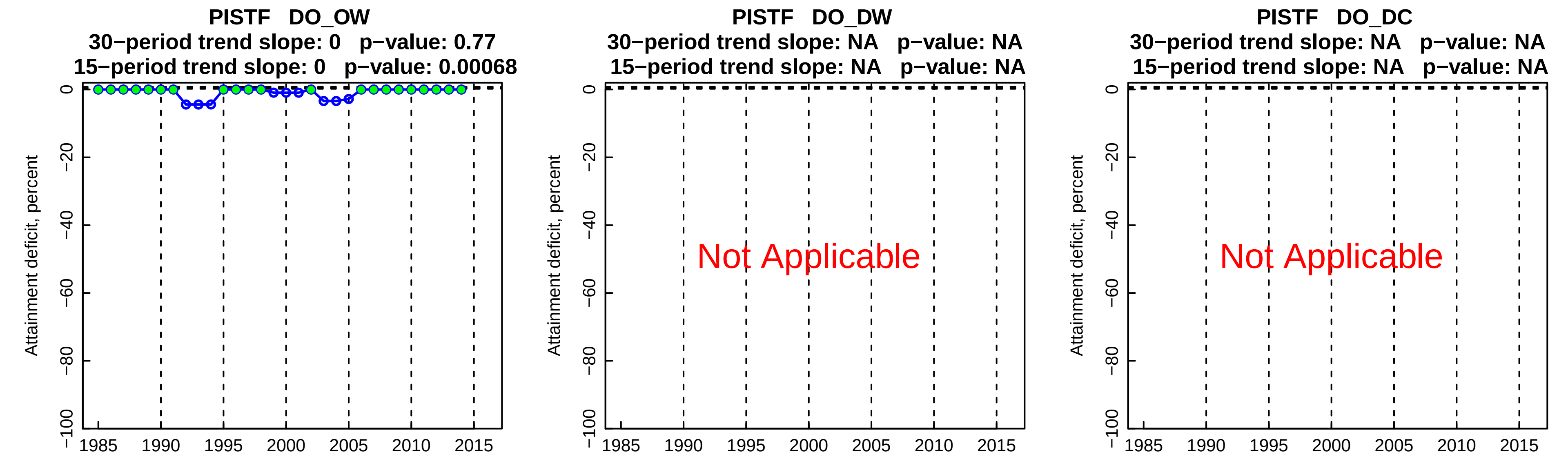
PISTF DO_OW
30−period trend slope: 0 p−value: 0.77
15−period trend slope: 0 p−value: 0.00068
PISTF DO_DW
30−period trend slope: NA p−value: NA
15−period trend slope: NA p−value: NA
Not Applicable
PISTF DO_DC
30−period trend slope: NA p−value: NA
15−period trend slope: NA p−value: NA
Not Applicable
Attainment deficit, percent
0
−20
−40
−60
−80
−100
1985
1990
1995
2000
2005
2010
2015

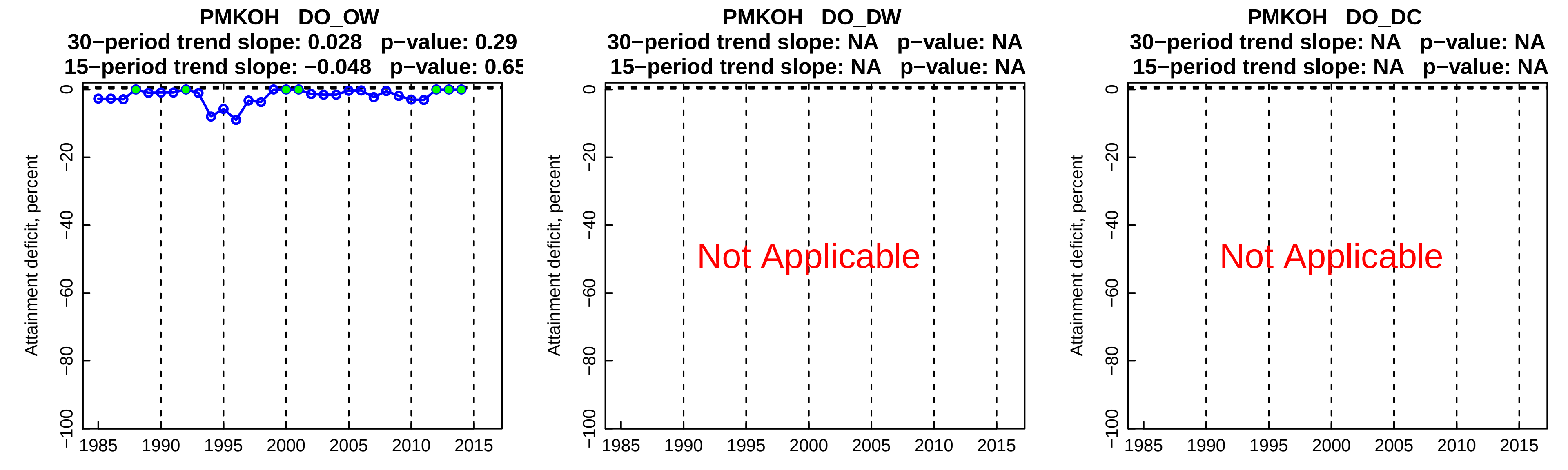
PMKOH DO_OW
30−period trend slope: 0.028 p−value: 0.29
15−period trend slope: −0.048 p−value: 0.65
Attainment deficit, percent
0
−20
−40
−60
−80
−100
1985 1990 1995 2000 2005 2010 2015
PMKOH DO_DW
30−period trend slope: NA p−value: NA
15−period trend slope: NA p−value: NA
Not Applicable
PMKOH DO_DC
30−period trend slope: NA p−value: NA
15−period trend slope: NA p−value: NA
Not Applicable

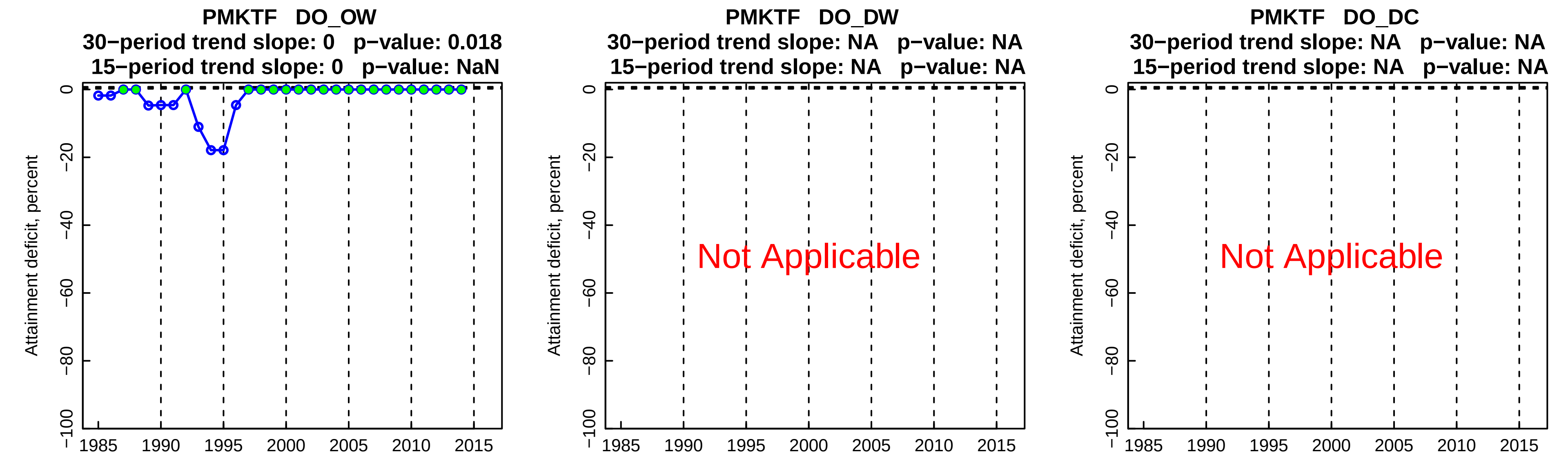

PMKTF DO_OW
30−period trend slope: 0 p−value: 0.018
15−period trend slope: 0 p−value: NaN
Attainment deficit, percent
0
−20
−40
−60
−80
−100
1985
1990
1995
2000
2005
2010
2015
PMKTF DO_DW
30−period trend slope: NA p−value: NA
15−period trend slope: NA p−value: NA
Not Applicable
PMKTF DO_DC
30−period trend slope: NA p−value: NA
15−period trend slope: NA p−value: NA
Not Applicable

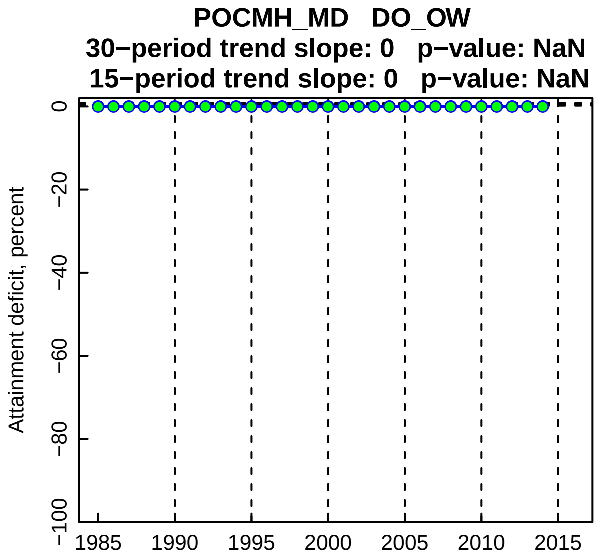

POCMH_MD DO_OW
30−period trend slope: 0 p−value: NaN
15−period trend slope: 0 p−value: NaN
Attainment deficit, percent
0
−20
−40
−60
−80
−100
1985
1990
1995
2000
2005
2010
2015

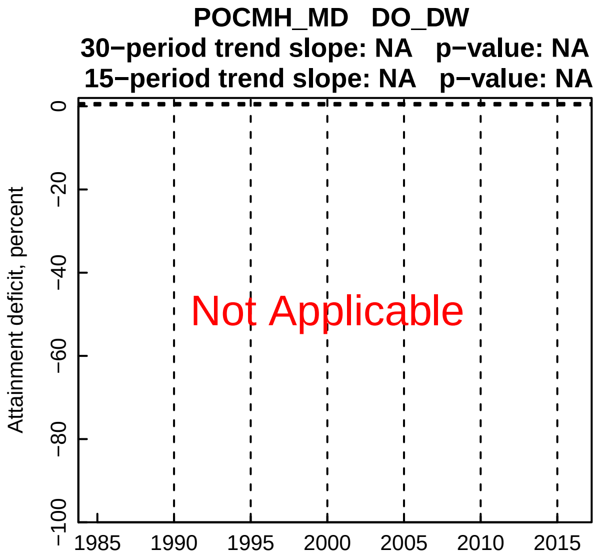

POCMH_MD DO_DW
30−period trend slope: NA p−value: NA
15−period trend slope: NA p−value: NA
Attainment deficit, percent
Not Applicable
0
−20
−40
−60
−80
−100
1985
1990
1995
2000
2005
2010
2015

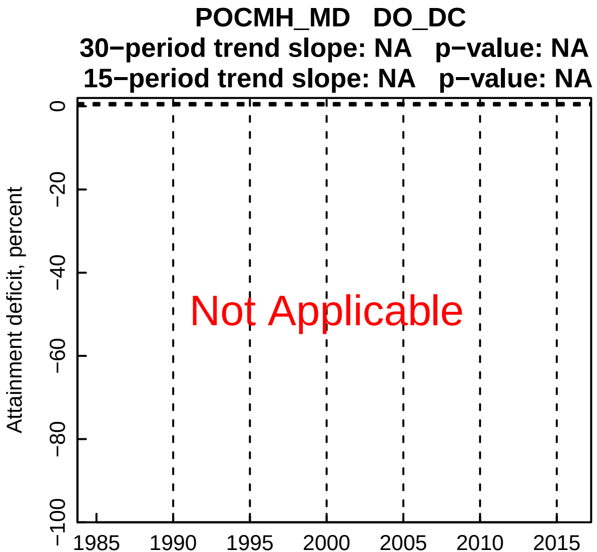

POCMH_MD DO_DC
30−period trend slope: NA p−value: NA
15−period trend slope: NA p−value: NA
Attainment deficit, percent
Not Applicable
0
−20
−40
−60
−80
−100
1985
1990
1995
2000
2005
2010
2015

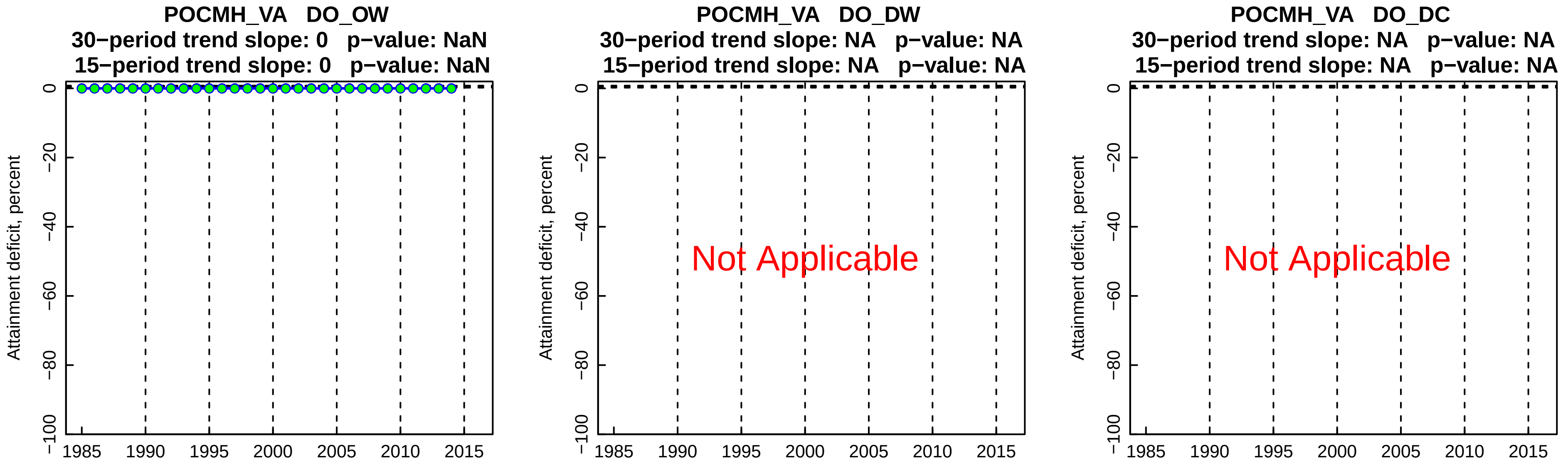
POCMH_VA DO_OW
30−period trend slope: 0 p−value: NaN
15−period trend slope: 0 p−value: NaN
POCMH_VA DO_DW
30−period trend slope: NA p−value: NA
15−period trend slope: NA p−value: NA
Not Applicable
POCMH_VA DO_DC
30−period trend slope: NA p−value: NA
15−period trend slope: NA p−value: NA
Not Applicable
Attainment deficit, percent
0
−20
−40
−60
−80
−100
1985
1990
1995
2000
2005
2010
2015

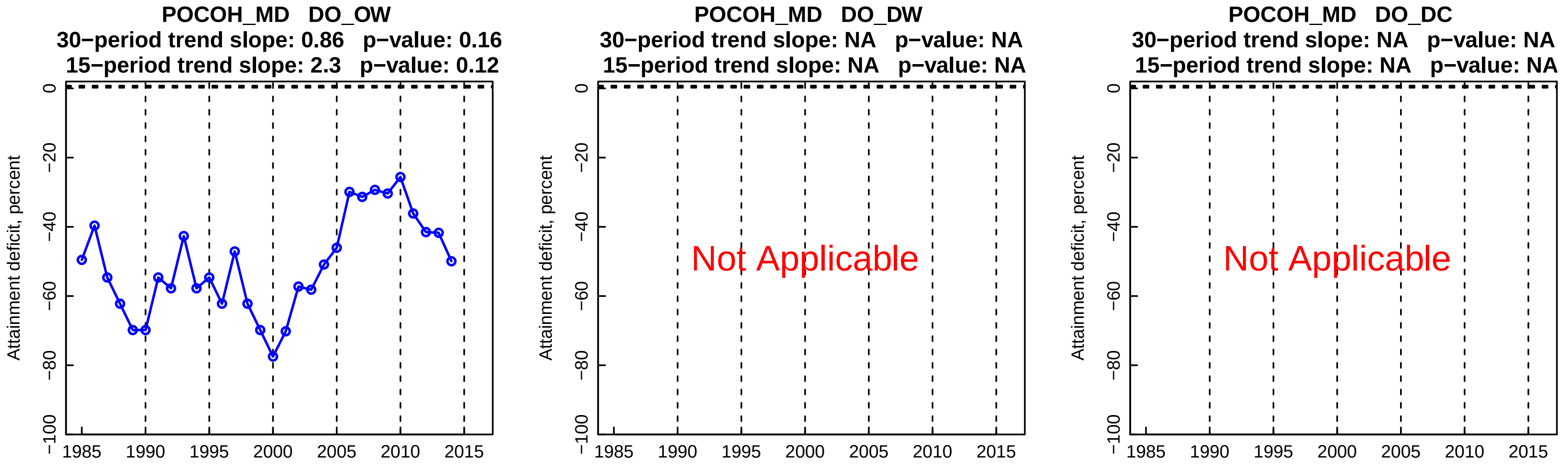
POCOH_MD DO_OW
30−period trend slope: 0.86 p−value: 0.16
15−period trend slope: 2.3 p−value: 0.12
Attainment deficit, percent
0
−20
−40
−60
−80
−100
1985
1990
1995
2000
2005
2010
2015
POCOH_MD DO_DW
30−period trend slope: NA p−value: NA
15−period trend slope: NA p−value: NA
Not Applicable
POCOH_MD DO_DC
30−period trend slope: NA p−value: NA
15−period trend slope: NA p−value: NA
Not Applicable

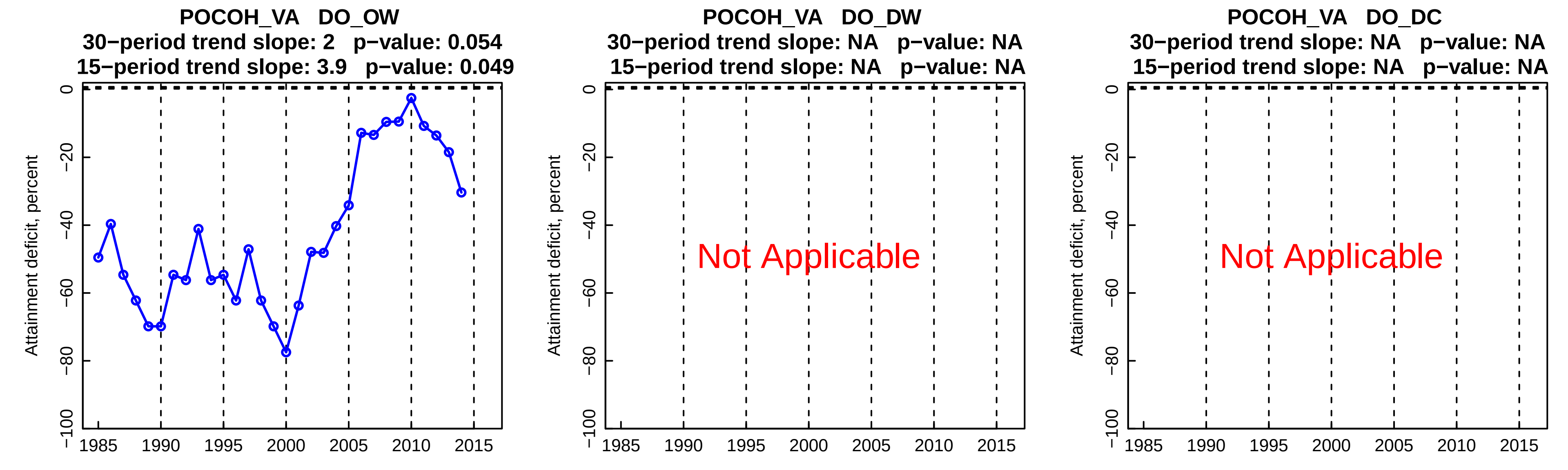
POCOH_VA DO_OW
30−period trend slope: 2 p−value: 0.054
15−period trend slope: 3.9 p−value: 0.049
Attainment deficit, percent
POCOH_VA DO_DW
30−period trend slope: NA p−value: NA
15−period trend slope: NA p−value: NA
Not Applicable
POCOH_VA DO_DC
30−period trend slope: NA p−value: NA
15−period trend slope: NA p−value: NA
Not Applicable

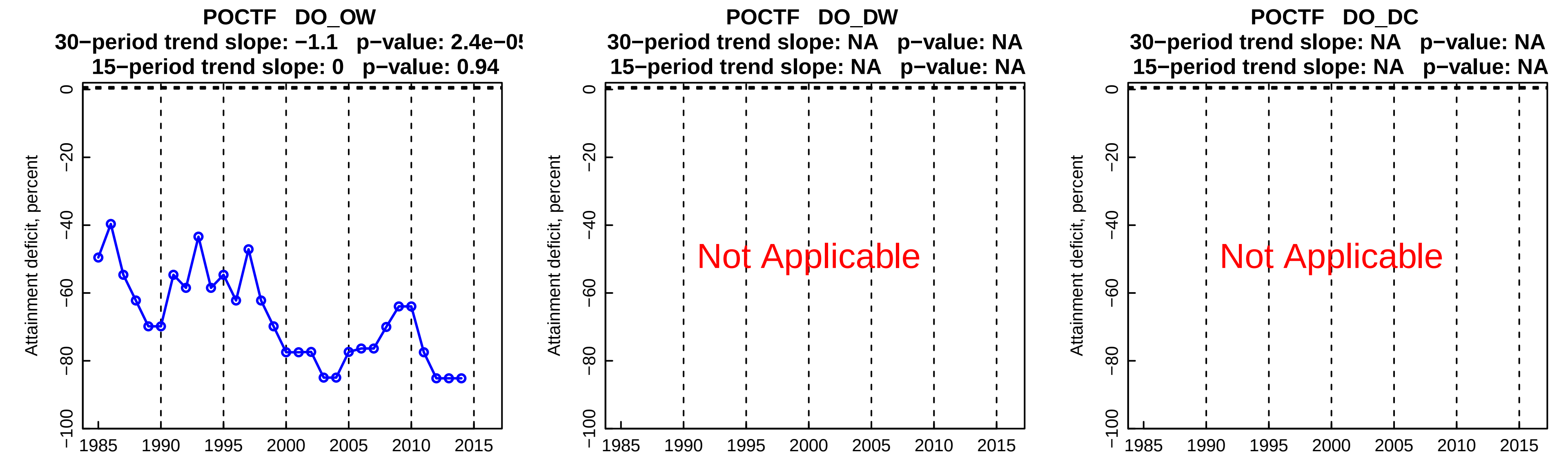
POCTF DO_OW
30−period trend slope: −1.1 p−value: 2.4e−0
15−period trend slope: 0 p−value: 0.94
Attainment deficit, percent
0
−20
−40
−60
−80
−100
1985
1990
1995
2000
2005
2010
2015
POCTF DO_DW
30−period trend slope: NA p−value: NA
15−period trend slope: NA p−value: NA
Not Applicable
POCTF DO_DC
30−period trend slope: NA p−value: NA
15−period trend slope: NA p−value: NA
Not Applicable

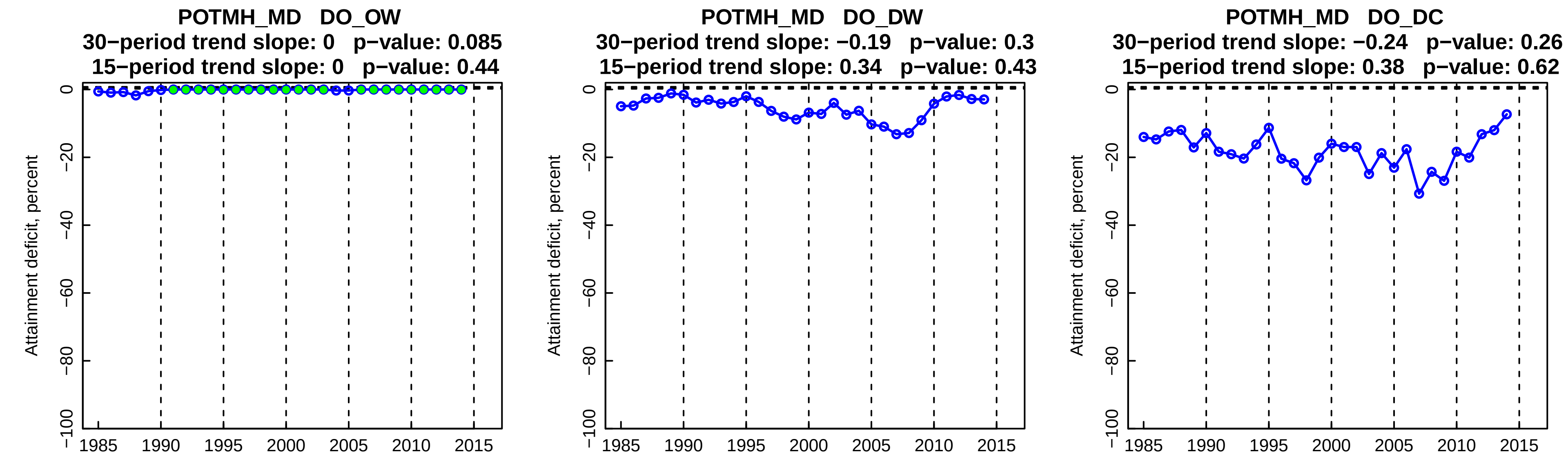
POTMH_MD DO_OW
30−period trend slope: 0 p−value: 0.085
15−period trend slope: 0 p−value: 0.44
POTMH_MD DO_DW
30−period trend slope: −0.19 p−value: 0.3
15−period trend slope: 0.34 p−value: 0.43
POTMH_MD DO_DC
30−period trend slope: −0.24 p−value: 0.26
15−period trend slope: 0.38 p−value: 0.62
Attainment deficit, percent
0
−20
−40
−60
−80
−100
1985
1990
1995
2000
2005
2010
2015

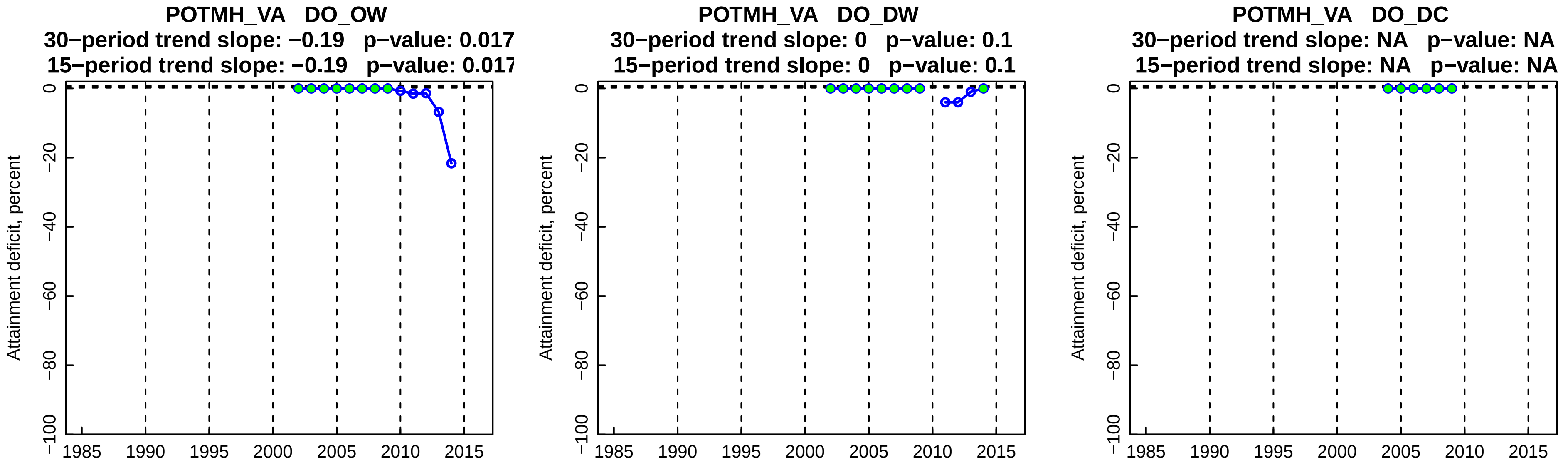
POTMH_VA DO_OW
30−period trend slope: −0.19 p−value: 0.017
15−period trend slope: −0.19 p−value: 0.017
Attainment deficit, percent
0
−20
−40
−60
−80
−100
1985
1990
1995
2000
2005
2010
2015
POTMH_VA DO_DW
30−period trend slope: 0 p−value: 0.1
15−period trend slope: 0 p−value: 0.1
Attainment deficit, percent
0
−20
−40
−60
−80
−100
1985
1990
1995
2000
2005
2010
2015
POTMH_VA DO_DC
30−period trend slope: NA p−value: NA
15−period trend slope: NA p−value: NA
Attainment deficit, percent
0
−20
−40
−60
−80
−100
1985
1990
1995
2000
2005
2010
2015

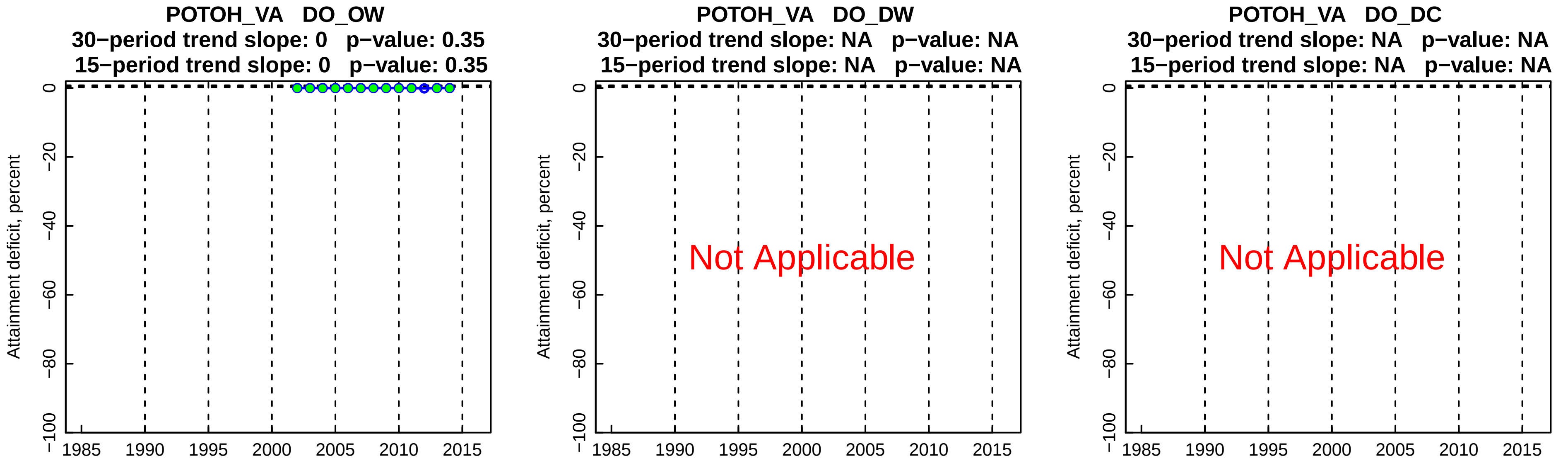
POTOH_VA DO_OW
30−period trend slope: 0 p−value: 0.35
15−period trend slope: 0 p−value: 0.35
Attainment deficit, percent
0
−20
−40
−60
−80
−100
1985
1990
1995
2000
2005
2010
2015
POTOH_VA DO_DW
30−period trend slope: NA p−value: NA
15−period trend slope: NA p−value: NA
Not Applicable
POTOH_VA DO_DC
30−period trend slope: NA p−value: NA
15−period trend slope: NA p−value: NA
Not Applicable

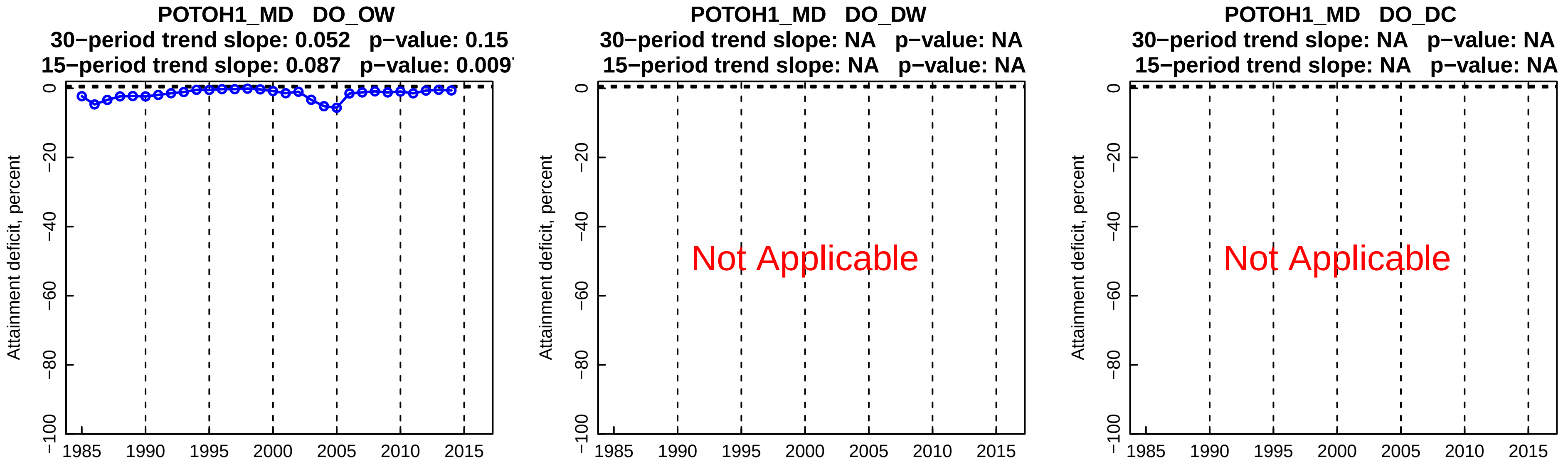

POTOH1_MD DO_OW
30−period trend slope: 0.052 p−value: 0.15
15−period trend slope: 0.087 p−value: 0.009
Attainment deficit, percent
0
−20
−40
−60
−80
−100
1985
1990
1995
2000
2005
2010
2015
POTOH1_MD DO_DW
30−period trend slope: NA p−value: NA
15−period trend slope: NA p−value: NA
Not Applicable
POTOH1_MD DO_DC
30−period trend slope: NA p−value: NA
15−period trend slope: NA p−value: NA
Not Applicable

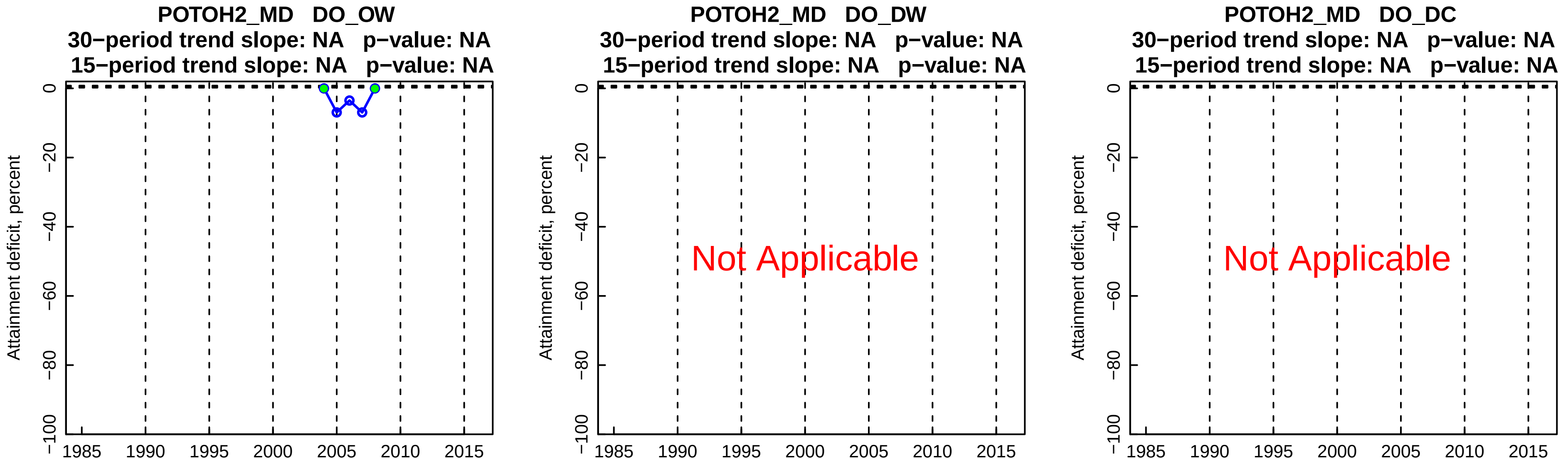
POTOH2_MD DO_OW
30−period trend slope: NA p−value: NA
15−period trend slope: NA p−value: NA
Attainment deficit, percent
0
−20
−40
−60
−80
−100
1985
1990
1995
2000
2005
2010
2015
POTOH2_MD DO_DW
30−period trend slope: NA p−value: NA
15−period trend slope: NA p−value: NA
Not Applicable
POTOH2_MD DO_DC
30−period trend slope: NA p−value: NA
15−period trend slope: NA p−value: NA
Not Applicable

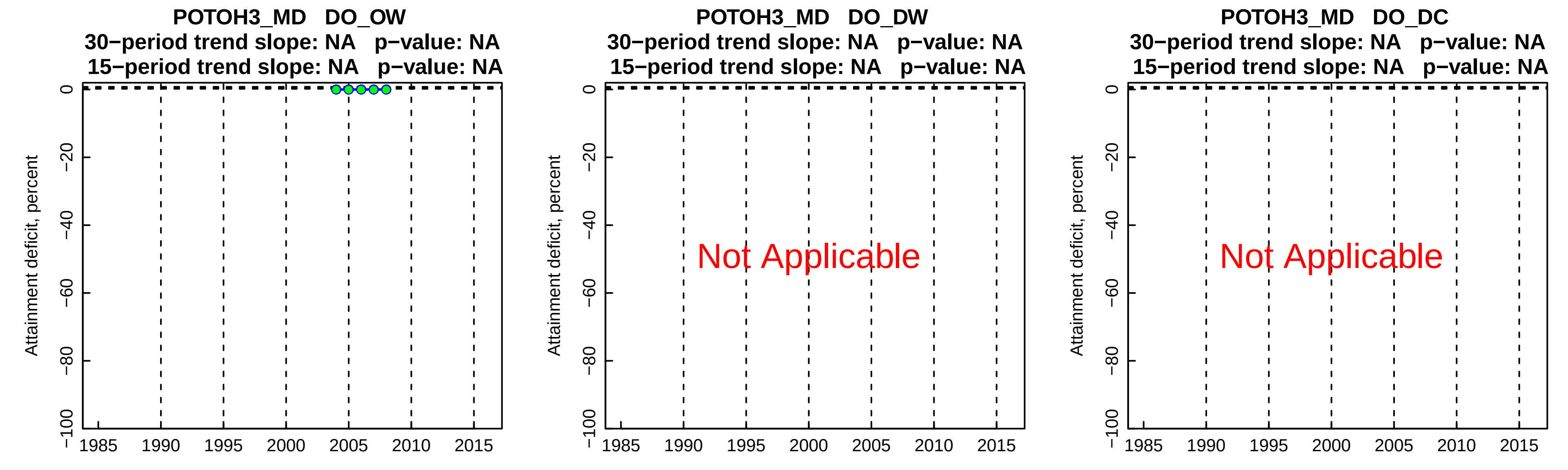

POTOH3_MD DO_OW
30−period trend slope: NA p−value: NA
15−period trend slope: NA p−value: NA
Attainment deficit, percent
0
−20
−40
−60
−80
−100
1985
1990
1995
2000
2005
2010
2015
POTOH3_MD DO_DW
30−period trend slope: NA p−value: NA
15−period trend slope: NA p−value: NA
Not Applicable
POTOH3_MD DO_DC
30−period trend slope: NA p−value: NA
15−period trend slope: NA p−value: NA
Not Applicable

POTTF_DC DO_OW
30−period trend slope: 0.008 p−value: 0.083
15−period trend slope: 0.00015 p−value: 0.000

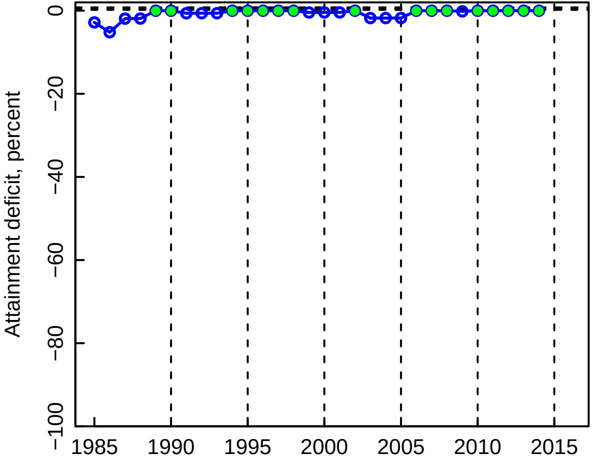

POTTF_DC DO_DW
30−period trend slope: NA p−value: NA
15−period trend slope: NA p−value: NA

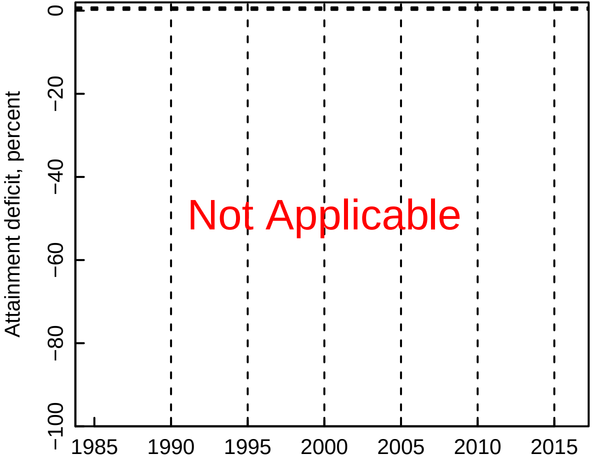

POTTF_DC DO_DC
30−period trend slope: NA p−value: NA
15−period trend slope: NA p−value: NA

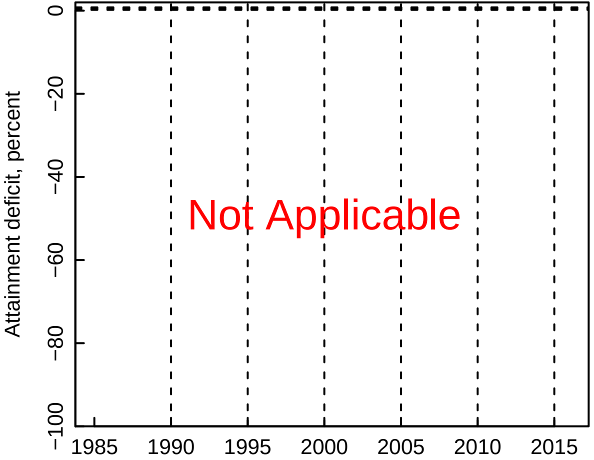

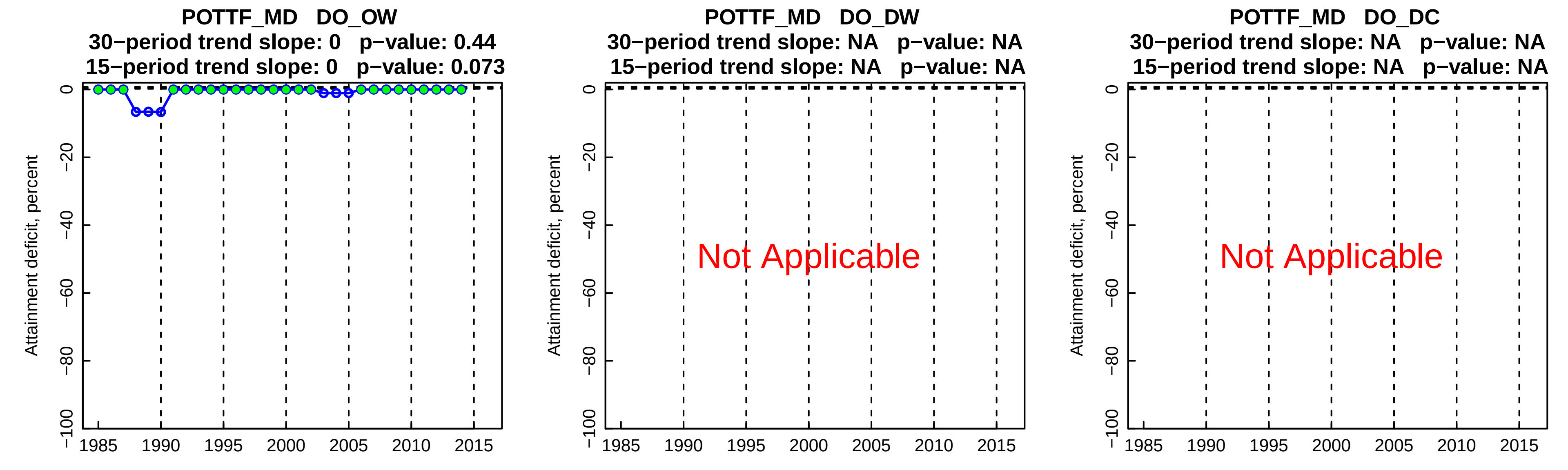

POTTF_MD DO_OW
30−period trend slope: 0 p−value: 0.44
15−period trend slope: 0 p−value: 0.073
Attainment deficit, percent
0
−20
−40
−60
−80
−100
1985
1990
1995
2000
2005
2010
2015
POTTF_MD DO_DW
30−period trend slope: NA p−value: NA
15−period trend slope: NA p−value: NA
Not Applicable
POTTF_MD DO_DC
30−period trend slope: NA p−value: NA
15−period trend slope: NA p−value: NA
Not Applicable

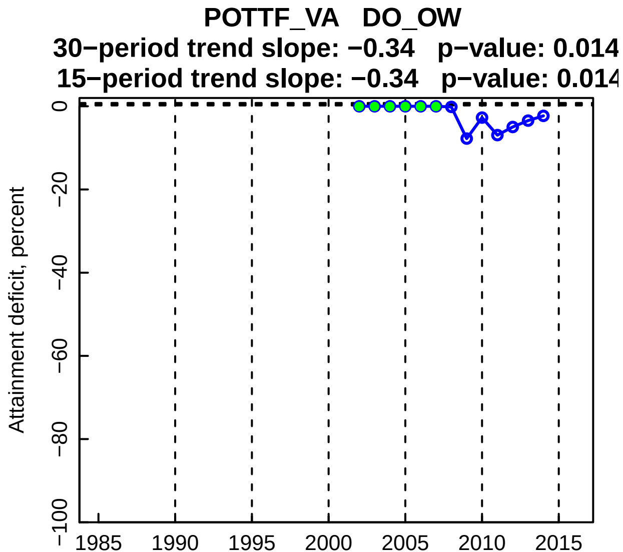
POTTF_VA DO_OW
30−period trend slope: −0.34 p−value: 0.014
15−period trend slope: −0.34 p−value: 0.014
Attainment deficit, percent
0
−20
−40
−60
−80
−100
1985
1990
1995
2000
2005
2010
2015

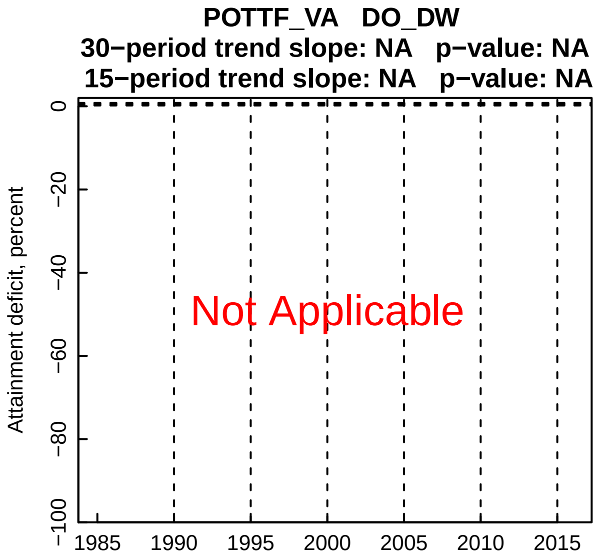
POTTF_VA DO_DW
30−period trend slope: NA p−value: NA
15−period trend slope: NA p−value: NA
Attainment deficit, percent
Not Applicable
0
−20
−40
−60
−80
−100
1985
1990
1995
2000
2005
2010
2015

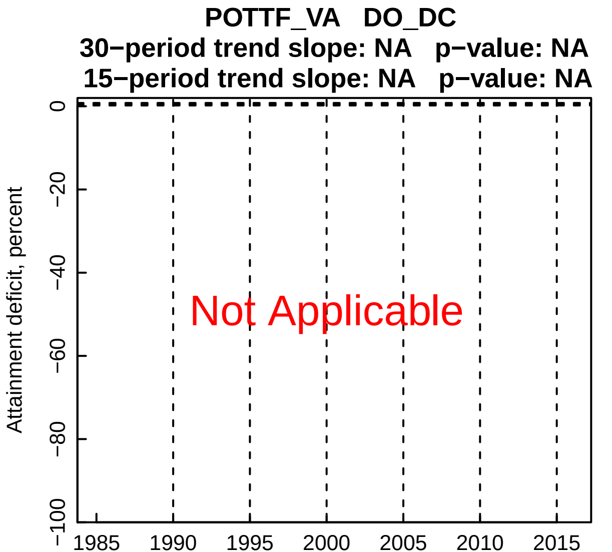
POTTF_VA DO_DC
30−period trend slope: NA p−value: NA
15−period trend slope: NA p−value: NA
Attainment deficit, percent
Not Applicable
0
−20
−40
−60
−80
−100
1985
1990
1995
2000
2005
2010
2015

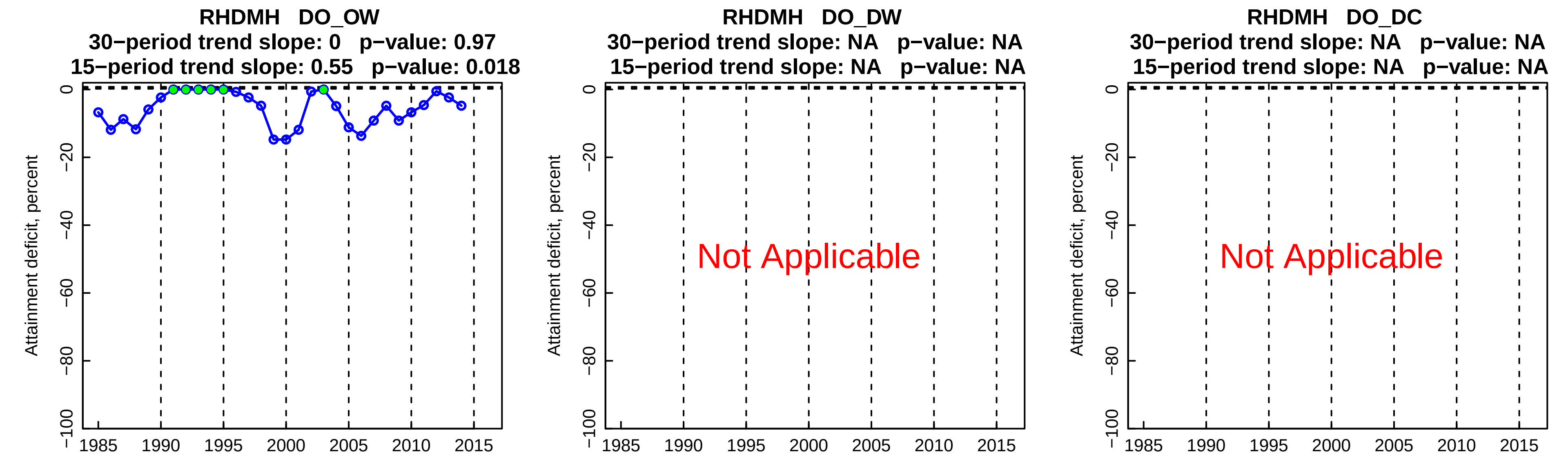
RHDMH DO_OW
30−period trend slope: 0 p−value: 0.97
15−period trend slope: 0.55 p−value: 0.018
Attainment deficit, percent
RHDMH DO_DW
30−period trend slope: NA p−value: NA
15−period trend slope: NA p−value: NA
Not Applicable
RHDMH DO_DC
30−period trend slope: NA p−value: NA
15−period trend slope: NA p−value: NA
Not Applicable

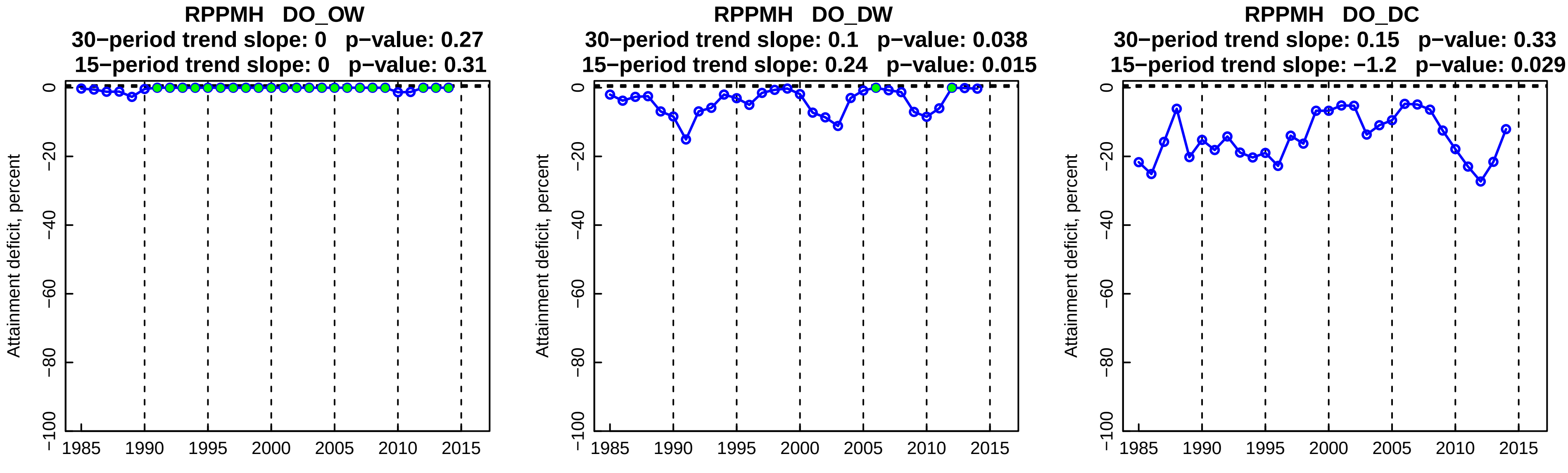
RPPMH DO_OW
30−period trend slope: 0 p−value: 0.27
15−period trend slope: 0 p−value: 0.31
Attainment deficit, percent
0
−20
−40
−60
−80
−100
1985
1990
1995
2000
2005
2010
2015
RPPMH DO_DW
30−period trend slope: 0.1 p−value: 0.038
15−period trend slope: 0.24 p−value: 0.015
RPPMH DO_DC
30−period trend slope: 0.15 p−value: 0.33
15−period trend slope: −1.2 p−value: 0.029

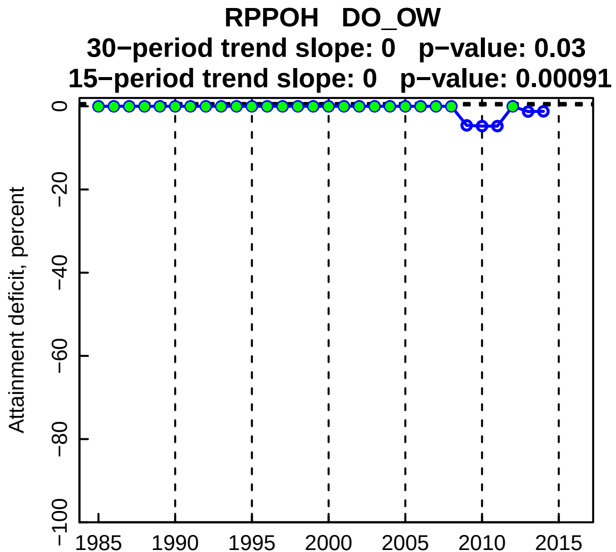
RPPOH DO_OW
30−period trend slope: 0 p−value: 0.03
15−period trend slope: 0 p−value: 0.00091
Attainment deficit, percent
0
−20
−40
−60
−80
−100
1985
1990
1995
2000
2005
2010
2015

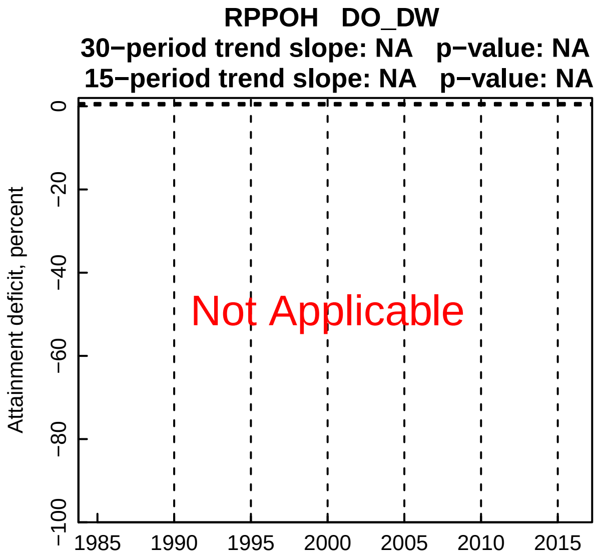
RPPOH DO_DW
30−period trend slope: NA p−value: NA
15−period trend slope: NA p−value: NA
Attainment deficit, percent
Not Applicable
0
−20
−40
−60
−80
−100
1985
1990
1995
2000
2005
2010
2015

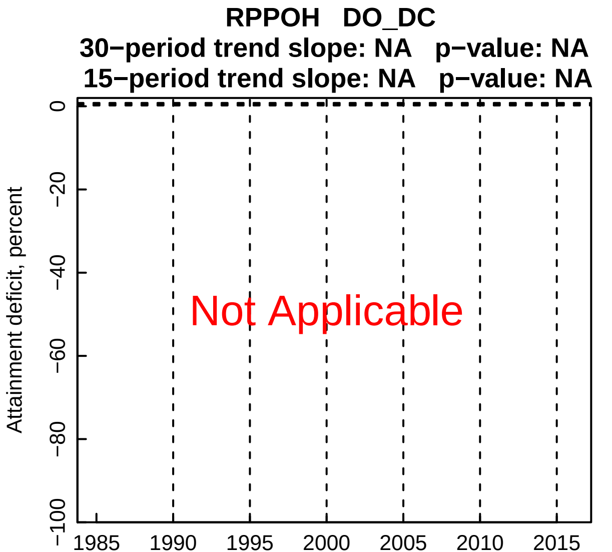
RPPOH DO_DC
30−period trend slope: NA p−value: NA
15−period trend slope: NA p−value: NA
Attainment deficit, percent
Not Applicable
0
−20
−40
−60
−80
−100
1985
1990
1995
2000
2005
2010
2015

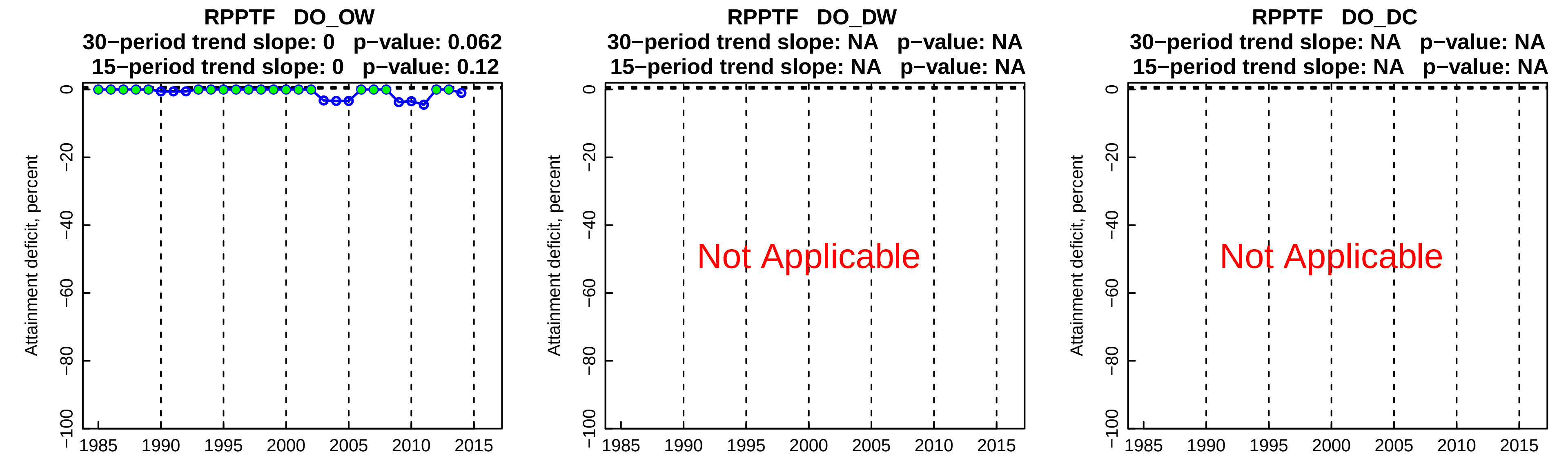
RPPTF DO_OW
30−period trend slope: 0 p−value: 0.062
15−period trend slope: 0 p−value: 0.12
Attainment deficit, percent
0
−20
−40
−60
−80
−100
1985
1990
1995
2000
2005
2010
2015
RPPTF DO_DW
30−period trend slope: NA p−value: NA
15−period trend slope: NA p−value: NA
Not Applicable
RPPTF DO_DC
30−period trend slope: NA p−value: NA
15−period trend slope: NA p−value: NA
Not Applicable

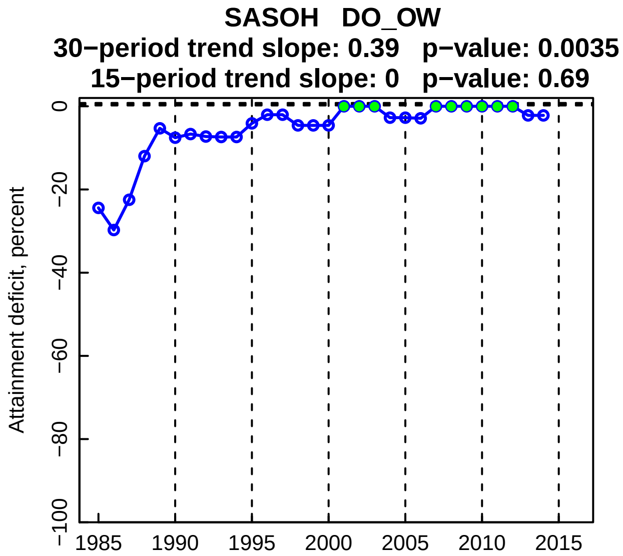

SASOH DO_OW
30−period trend slope: 0.39 p−value: 0.0035
15−period trend slope: 0 p−value: 0.69
Attainment deficit, percent
0
−20
−40
−60
−80
−100
1985
1990
1995
2000
2005
2010
2015

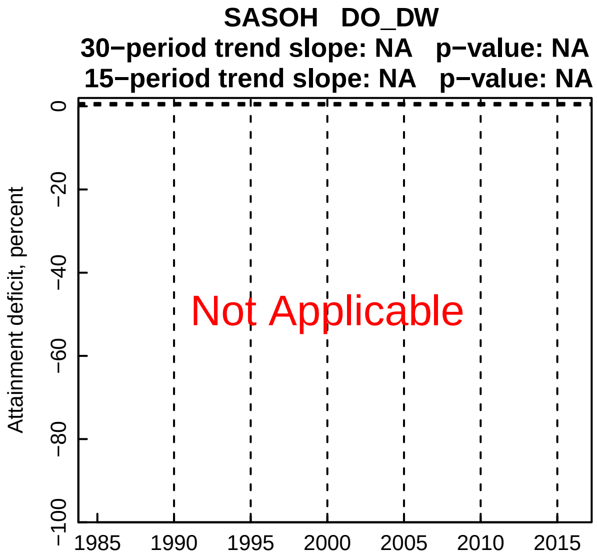

SASOH DO_DW
30−period trend slope: NA p−value: NA
15−period trend slope: NA p−value: NA
Attainment deficit, percent
Not Applicable
0
−20
−40
−60
−80
−100
1985
1990
1995
2000
2005
2010
2015

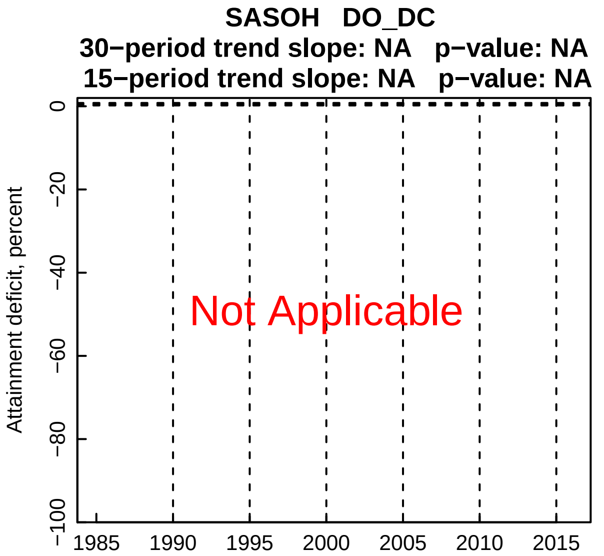

SASOH DO_DC
30−period trend slope: NA p−value: NA
15−period trend slope: NA p−value: NA
Attainment deficit, percent
Not Applicable
0
−20
−40
−60
−80
−100
1985
1990
1995
2000
2005
2010
2015

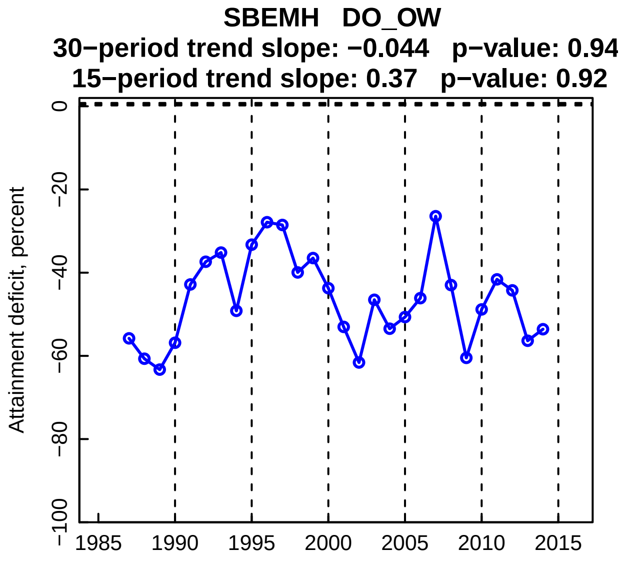

SBEMH DO_OW
30−period trend slope: −0.044 p−value: 0.94
15−period trend slope: 0.37 p−value: 0.92
Attainment deficit, percent
0
−20
−40
−60
−80
−100
1985
1990
1995
2000
2005
2010
2015

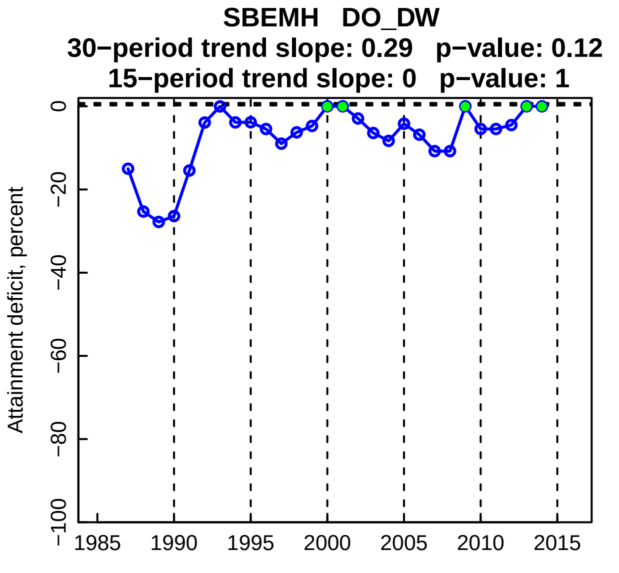

SBEMH DO_DW
30−period trend slope: 0.29 p−value: 0.12
15−period trend slope: 0 p−value: 1
Attainment deficit, percent
0
−20
−40
−60
−80
−100
1985
1990
1995
2000
2005
2010
2015

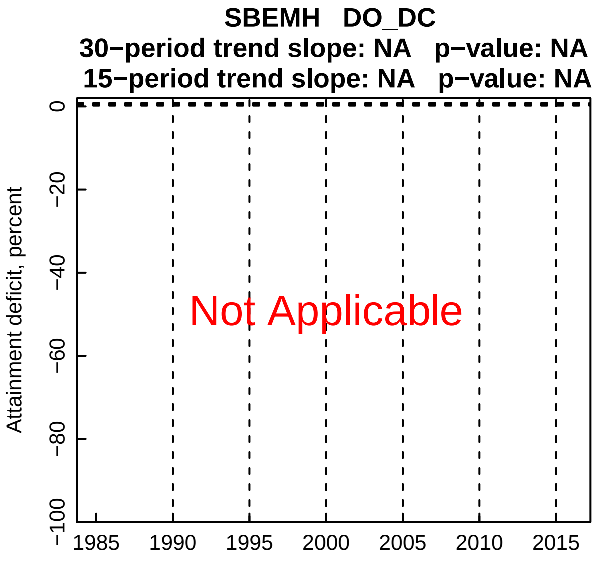

SBEMH DO_DC
30−period trend slope: NA p−value: NA
15−period trend slope: NA p−value: NA
Attainment deficit, percent
Not Applicable
0
−20
−40
−60
−80
−100
1985
1990
1995
2000
2005
2010
2015

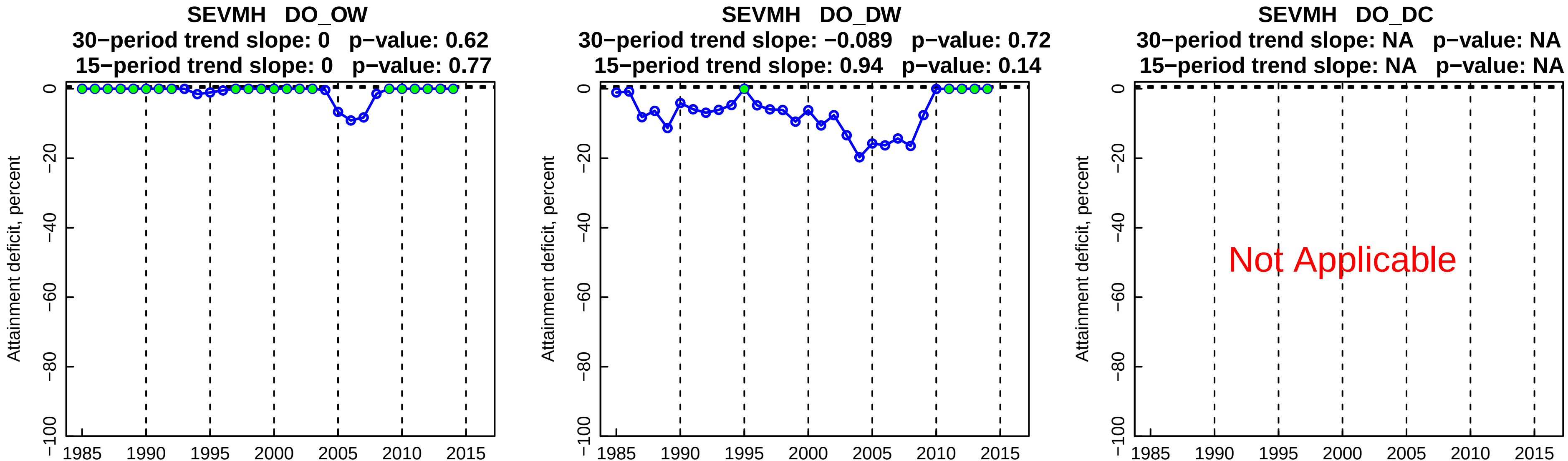

SEVMH DO_OW
30−period trend slope: 0 p−value: 0.62
15−period trend slope: 0 p−value: 0.77
Attainment deficit, percent
0
−20
−40
−60
−80
−100
1985
1990
1995
2000
2005
2010
2015
SEVMH DO_DW
30−period trend slope: −0.089 p−value: 0.72
15−period trend slope: 0.94 p−value: 0.14
Attainment deficit, percent
SEVMH DO_DC
30−period trend slope: NA p−value: NA
15−period trend slope: NA p−value: NA
Attainment deficit, percent
Not Applicable

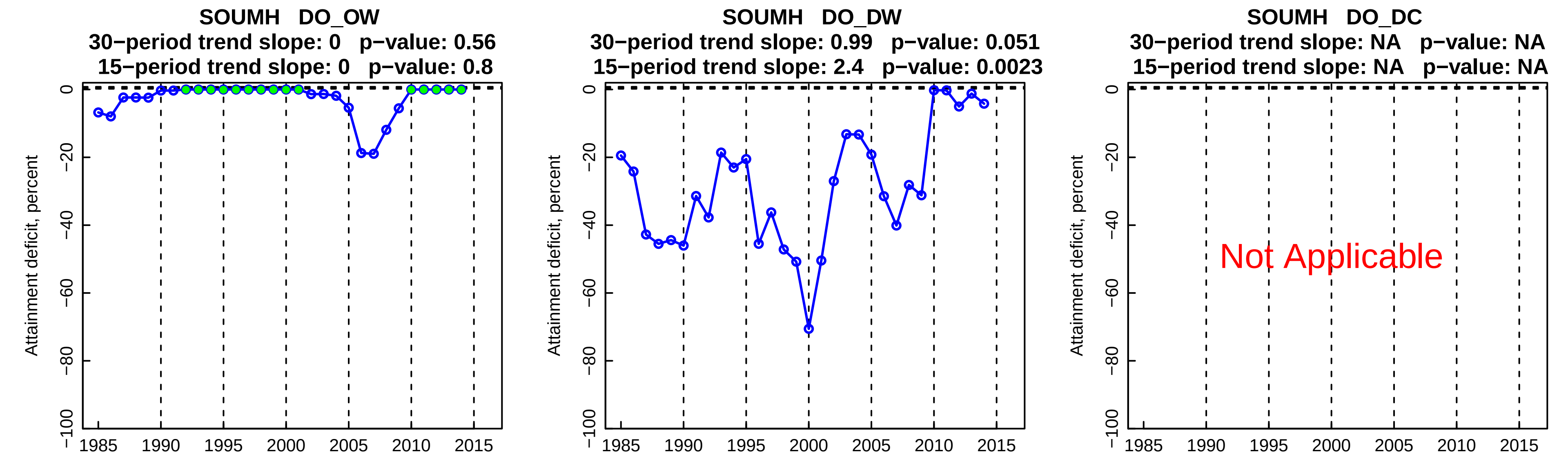
SOUMH DO_OW
30−period trend slope: 0 p−value: 0.56
15−period trend slope: 0 p−value: 0.8
Attainment deficit, percent
0
−20
−40
−60
−80
−100
1985
1990
1995
2000
2005
2010
2015
SOUMH DO_DW
30−period trend slope: 0.99 p−value: 0.051
15−period trend slope: 2.4 p−value: 0.0023
SOUMH DO_DC
30−period trend slope: NA p−value: NA
15−period trend slope: NA p−value: NA
Not Applicable

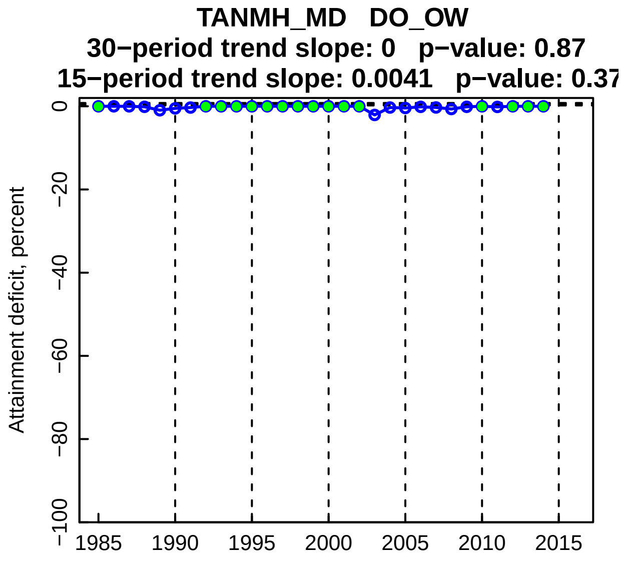

TANMH_MD DO_OW
30−period trend slope: 0 p−value: 0.87
15−period trend slope: 0.0041 p−value: 0.37
Attainment deficit, percent
0
−20
−40
−60
−80
−100
1985
1990
1995
2000
2005
2010
2015

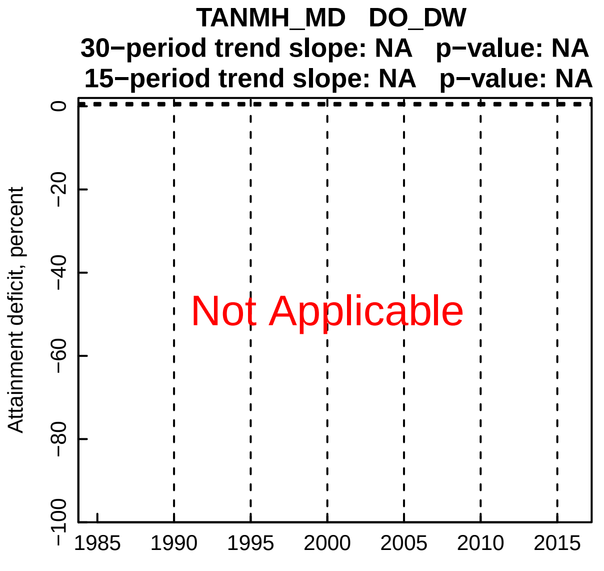

TANMH_MD DO_DW
30−period trend slope: NA p−value: NA
15−period trend slope: NA p−value: NA
Attainment deficit, percent
Not Applicable
0
−20
−40
−60
−80
−100
1985
1990
1995
2000
2005
2010
2015

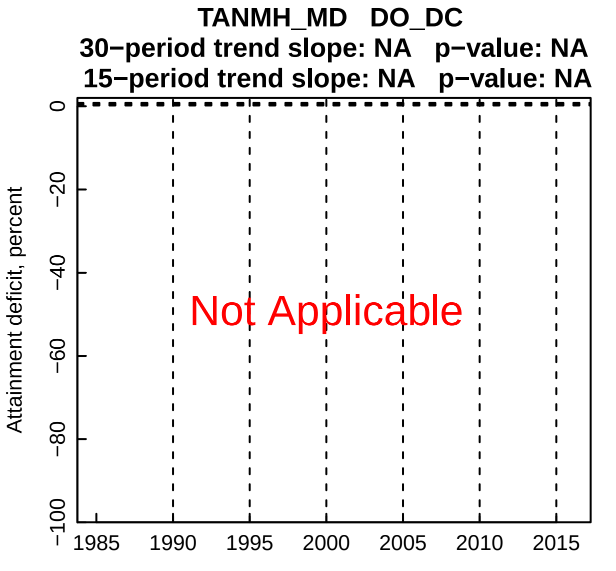

TANMH_MD DO_DC
30−period trend slope: NA p−value: NA
15−period trend slope: NA p−value: NA
Attainment deficit, percent
Not Applicable
0
−20
−40
−60
−80
−100
1985
1990
1995
2000
2005
2010
2015

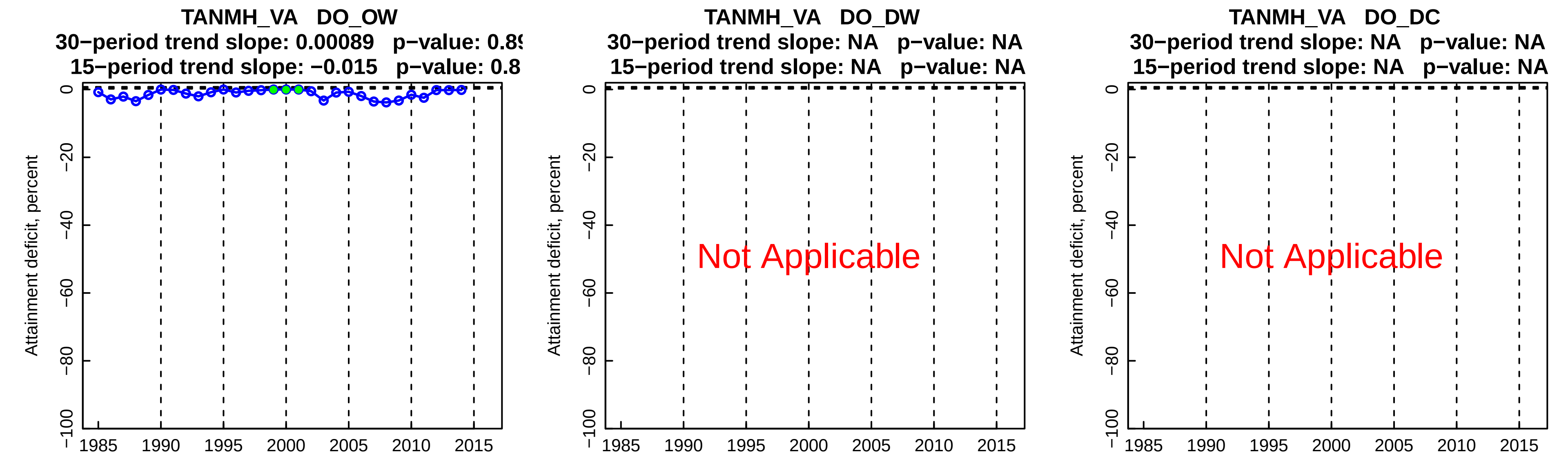

TANMH_VA DO_OW
30−period trend slope: 0.00089 p−value: 0.8
15−period trend slope: −0.015 p−value: 0.8
Attainment deficit, percent
0
−20
−40
−60
−80
−100
1985 1990 1995 2000 2005 2010 2015
TANMH_VA DO_DW
30−period trend slope: NA p−value: NA
15−period trend slope: NA p−value: NA
Not Applicable
TANMH_VA DO_DC
30−period trend slope: NA p−value: NA
15−period trend slope: NA p−value: NA
Not Applicable

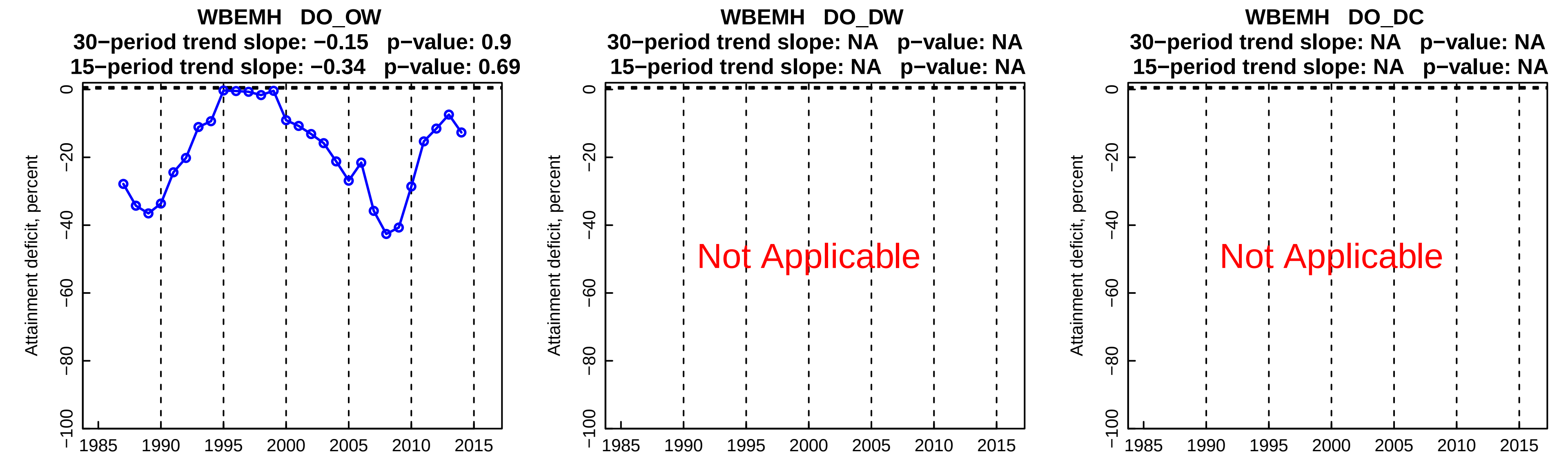
WBEMH DO_OW
30−period trend slope: −0.15 p−value: 0.9
15−period trend slope: −0.34 p−value: 0.69
Attainment deficit, percent
WBEMH DO_DW
30−period trend slope: NA p−value: NA
15−period trend slope: NA p−value: NA
Not Applicable
WBEMH DO_DC
30−period trend slope: NA p−value: NA
15−period trend slope: NA p−value: NA
Not Applicable

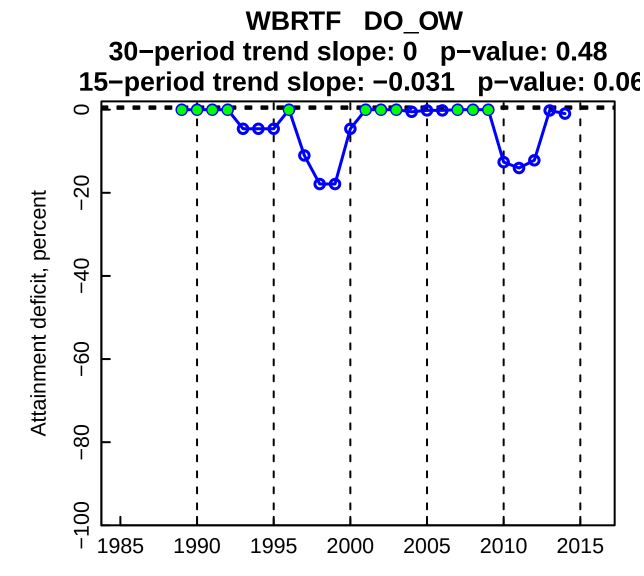
WBRTF DO_OW
30−period trend slope: 0 p−value: 0.48
15−period trend slope: −0.031 p−value: 0.06
Attainment deficit, percent
0
−20
−40
−60
−80
−100
1985
1990
1995
2000
2005
2010
2015

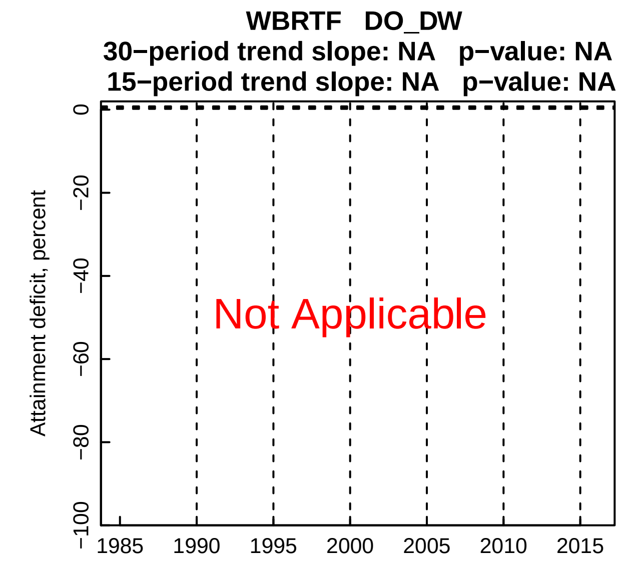
WBRTF DO_DW
30−period trend slope: NA p−value: NA
15−period trend slope: NA p−value: NA
Attainment deficit, percent
Not Applicable
0
−20
−40
−60
−80
−100
1985
1990
1995
2000
2005
2010
2015

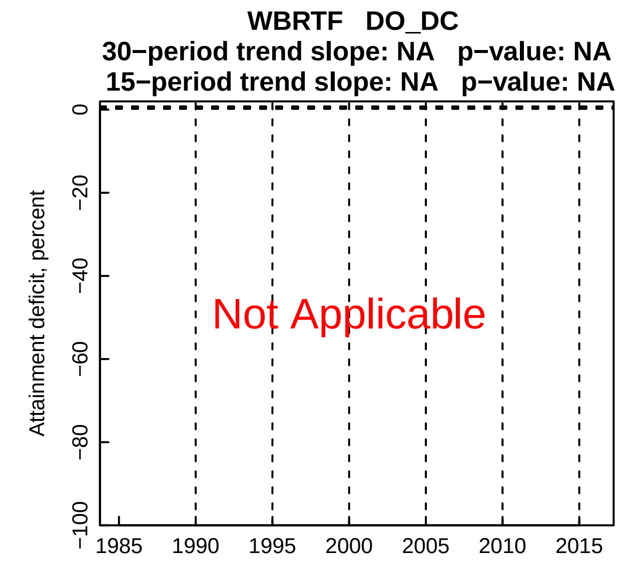
WBRTF DO_DC
30−period trend slope: NA p−value: NA
15−period trend slope: NA p−value: NA
Attainment deficit, percent
Not Applicable
0
−20
−40
−60
−80
−100
1985
1990
1995
2000
2005
2010
2015

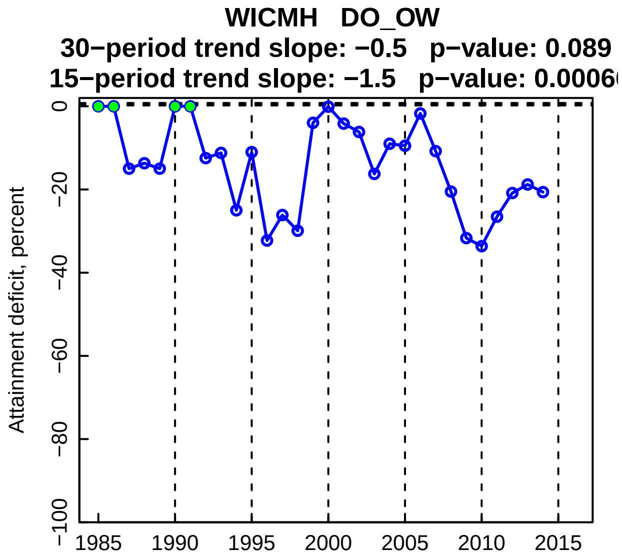
WICMH DO_OW
30−period trend slope: −0.5 p−value: 0.089
15−period trend slope: −1.5 p−value: 0.0006
Attainment deficit, percent
0
−20
−40
−60
−80
−100
1985 1990 1995 2000 2005 2010 2015

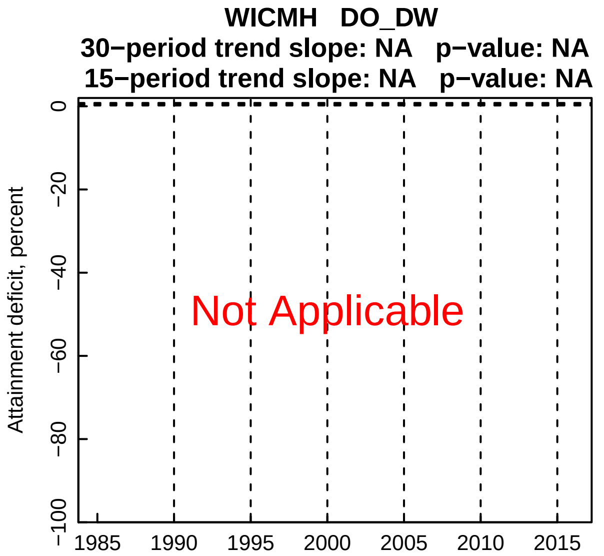
WICMH DO_DW
30−period trend slope: NA p−value: NA
15−period trend slope: NA p−value: NA
Attainment deficit, percent
0
−20
−40
−60
−80
−100
1985 1990 1995 2000 2005 2010 2015
Not Applicable

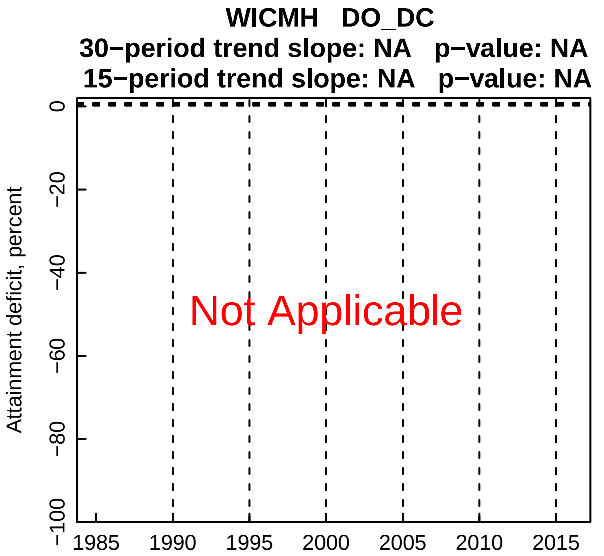
WICMH DO_DC
30−period trend slope: NA p−value: NA
15−period trend slope: NA p−value: NA
Attainment deficit, percent
0
−20
−40
−60
−80
−100
1985 1990 1995 2000 2005 2010 2015
Not Applicable

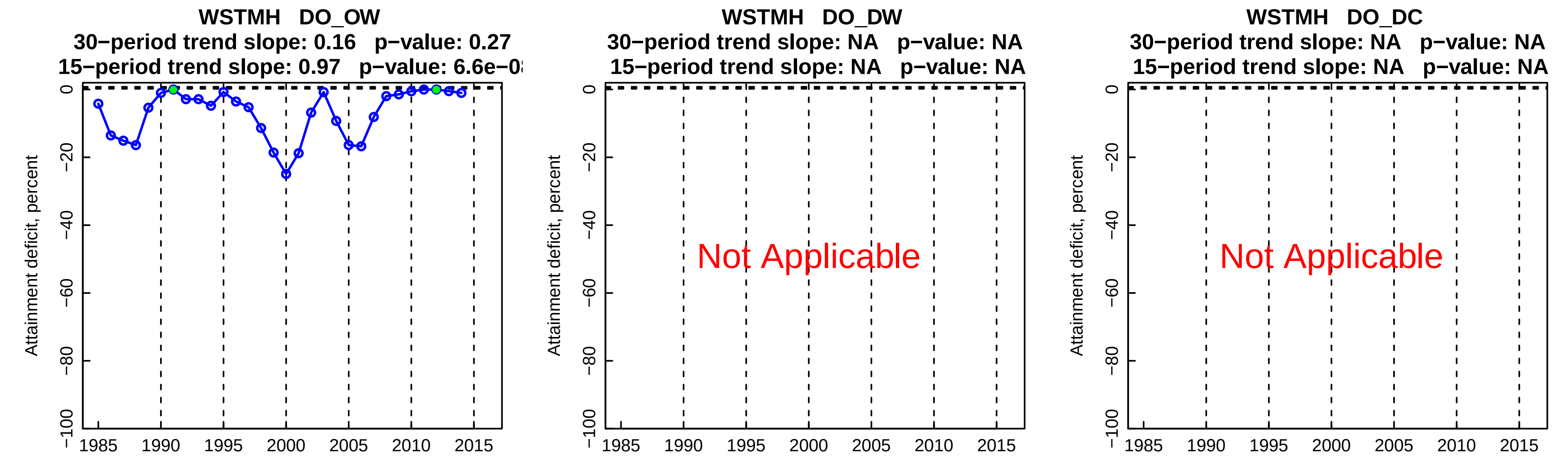

WSTMH DO_OW
30−period trend slope: 0.16 p−value: 0.27
15−period trend slope: 0.97 p−value: 6.6e−0
Attainment deficit, percent
0
−20
−40
−60
−80
−100
1985
1990
1995
2000
2005
2010
2015
WSTMH DO_DW
30−period trend slope: NA p−value: NA
15−period trend slope: NA p−value: NA
Not Applicable
WSTMH DO_DC
30−period trend slope: NA p−value: NA
15−period trend slope: NA p−value: NA
Not Applicable

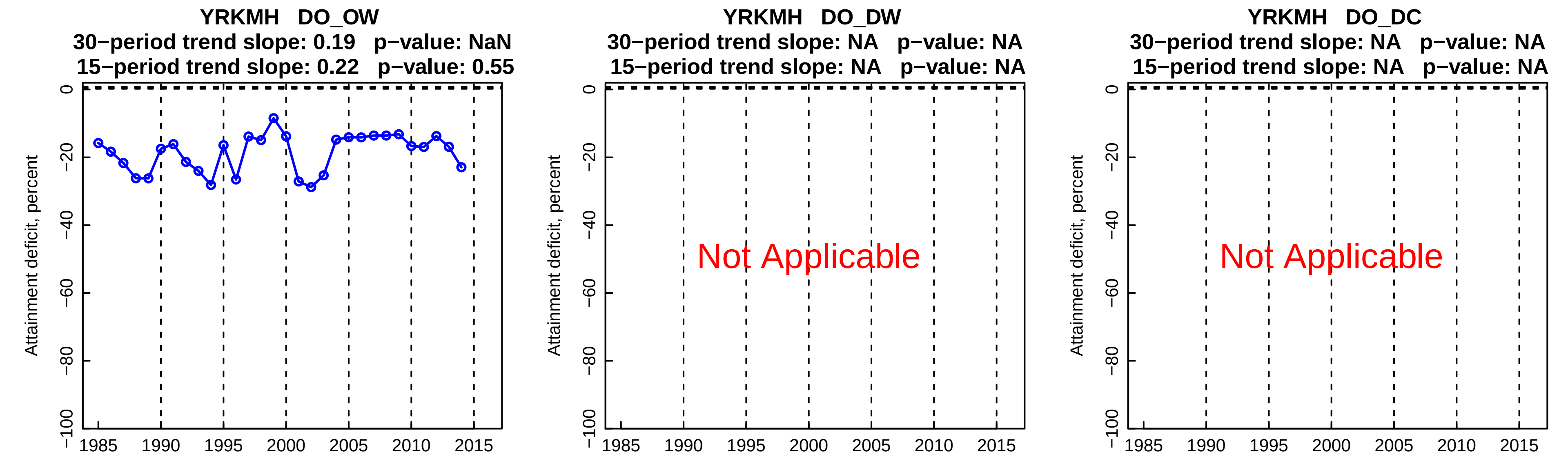

YRKMH DO_OW
30−period trend slope: 0.19 p−value: NaN
15−period trend slope: 0.22 p−value: 0.55
Attainment deficit, percent
0
−20
−40
−60
−80
−100
1985
1990
1995
2000
2005
2010
2015
YRKMH DO_DW
30−period trend slope: NA p−value: NA
15−period trend slope: NA p−value: NA
Not Applicable
YRKMH DO_DC
30−period trend slope: NA p−value: NA
15−period trend slope: NA p−value: NA
Not Applicable

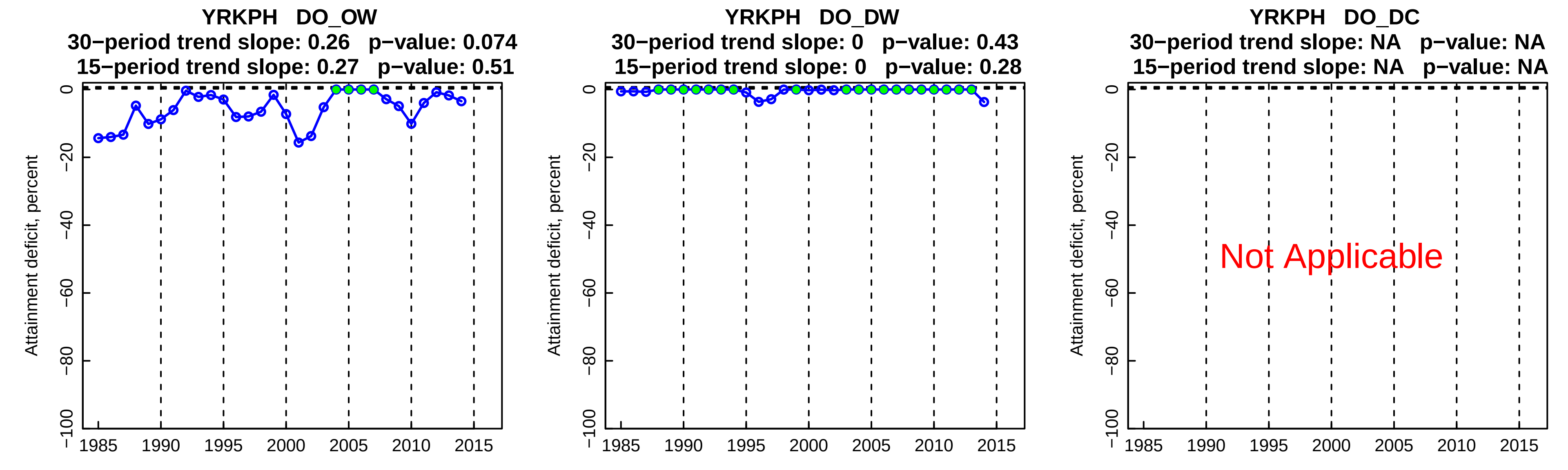
YRKPH DO_OW
30−period trend slope: 0.26 p−value: 0.074
15−period trend slope: 0.27 p−value: 0.51
YRKPH DO_DW
30−period trend slope: 0 p−value: 0.43
15−period trend slope: 0 p−value: 0.28
YRKPH DO_DC
30−period trend slope: NA p−value: NA
15−period trend slope: NA p−value: NA
Not Applicable
Attainment deficit, percent
0
−20
−40
−60
−80
−100
1985
1990
1995
2000
2005
2010
2015
